# Neurobiological Mechanisms of Nicotine Reward and Aversion

**DOI:** 10.1124/pharmrev.121.000299

**Published:** 2022-01

**Authors:** Lauren Wills, Jessica L. Ables, Kevin M. Braunscheidel, Stephanie P.B. Caligiuri, Karim S. Elayouby, Clementine Fillinger, Masago Ishikawa, Janna K. Moen, Paul J. Kenny

**Affiliations:** Nash Family Department of Neuroscience, Icahn School of Medicine at Mount Sinai, One Gustave L. Levy Place, New York, New York

## Abstract

Neuronal nicotinic acetylcholine receptors (nAChRs) regulate the rewarding actions of nicotine contained in tobacco that establish and maintain the smoking habit. nAChRs also regulate the aversive properties of nicotine, sensitivity to which decreases tobacco use and protects against tobacco use disorder. These opposing behavioral actions of nicotine reflect nAChR expression in brain reward and aversion circuits. nAChRs containing *α*4 and *β*2 subunits are responsible for the high-affinity nicotine binding sites in the brain and are densely expressed by reward-relevant neurons, most notably dopaminergic, GABAergic, and glutamatergic neurons in the ventral tegmental area. High-affinity nAChRs can incorporate additional subunits, including *β*3, *α*6, or *α*5 subunits, with the resulting nAChR subtypes playing discrete and dissociable roles in the stimulatory actions of nicotine on brain dopamine transmission. nAChRs in brain dopamine circuits also participate in aversive reactions to nicotine and the negative affective state experienced during nicotine withdrawal. nAChRs containing *α*3 and *β*4 subunits are responsible for the low-affinity nicotine binding sites in the brain and are enriched in brain sites involved in aversion, including the medial habenula, interpeduncular nucleus, and nucleus of the solitary tract, brain sites in which *α*5 nAChR subunits are also expressed. These aversion-related brain sites regulate nicotine avoidance behaviors, and genetic variation that modifies the function of nAChRs in these sites increases vulnerability to tobacco dependence and smoking-related diseases. Here, we review the molecular, cellular, and circuit-level mechanisms through which nicotine elicits reward and aversion and the adaptations in these processes that drive the development of nicotine dependence.

**Significance Statement:**

Tobacco use disorder in the form of habitual cigarette smoking or regular use of other tobacco-related products is a major cause of death and disease worldwide. This article reviews the actions of nicotine in the brain that contribute to tobacco use disorder.

## Graphical abstract

**Figure:**
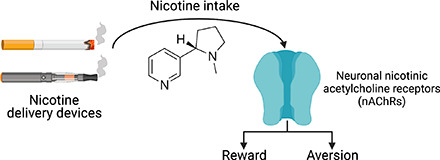

## I. Introduction

### A. Motivational Properties of Nicotine

Tobacco smoking results in more than 5 million deaths each year worldwide (WHO, 2008), and it is predicted that approximately 0.6 billion current smokers will die from smoking-related illnesses (Ezzati and Lopez, 2003; Doll et al., 2004; Coe et al., 2005; Mathers and Loncar, 2006). Even in nonsmokers tobacco can be deadly, with over 880,000 people worldwide estimated to die annually year from diseases related to secondhand smoke exposure (Oberg et al., 2011). According to the Centers for Disease Control and Prevention, an estimated 14% of adults in the United States (∼34.1 million people) were current cigarette smokers in 2019 (Statistics, 2018). This level of tobacco use represents an all-time low and the culmination of a trend that has been apparent since the mid-1960s, when approximately 45% of adults in the United States were current smokers. In contrast to this progress in reducing cigarette smoking in adults, use of noncigarette tobacco products, most notably electronic delivery devices, has increased dramatically over the past 10 years, particularly in school-age children. From 2011 to 2019, use of electronic cigarettes (e-cigarettes) among children increased >1500%, with almost 3 million children initiating e-cigarette use (7900 per day) in 2019 alone. Strikingly, ∼30% of high school students report use of a tobacco product. These young e-cigarette users show increased propensity to use conventional cigarettes and other combustible tobacco products relative to children who do not use e-cigarettes (Leventhal et al., 2015). As might be expected, habitual tobacco use is associated with diseases of the airways, including lung, larynx, and mouth cancers; chronic obstructive pulmonary disease; and asthma (Collaborators, 2017). Cigarette smoking is responsible for ∼90% of all lung cancers in the United States (Mokdad et al., 2004), with more people dying from this smoking-related disease than any other type of cancer. Tobacco smoking and e-cigarette use are also a major cause of nonrespiratory system diseases, including type 2 diabetes and cardiovascular disease (Rostron et al., 2014; Collaborators, 2017; Hedman et al., 2018; Xie et al., 2020). Smokers who quit before the onset of tobacco-related illness can largely avoid the increased mortality risk (Doll et al., 1994; Peto et al., 2000). Despite awareness of the dangers of smoking, approximately 80% of current smokers attempting to quit will relapse within the first month of abstinence (Benowitz, 2009). Current smoking cessation medications have limited utility. In smokers attempting to quit, ∼23% treated with Chantix (varenicline) and ∼16% treated with Zyban (bupropion) remain abstinent after 1 year compared with ∼9% of those treated with placebo (Knight et al., 2009). Pharmacotherapy is therefore an effective strategy to aid smoking cessation, but there remains considerable risk of relapse even when using the most effective medications currently available. The development of more efficacious smoking-cessation therapeutics would prevent the premature death of hundreds of thousands of people each year and is perhaps the most cost-saving intervention possible within a modern healthcare system (Knight et al., 2009). Leveraging our growing understanding of the basic neurobiological mechanisms of tobacco use disorder will likely lead to more effective smoking-cessation therapeutics.

Nicotine is the major rewarding component responsible for the reinforcing properties of cigarette smoke, which drive the development of tobacco use disorder (Stolerman and Jarvis, 1995). Nicotine intake produces a subjectively pleasant experience (reward), the obtaining of which increases the likelihood that smoking behavior will occur again (reinforcement) (Fowler and Kenny, 2011). Nicotine has both positive and negative reinforcing properties, meaning that the drug enhances the activity of brain reward circuits (positive reinforcement) while attenuating the activity of brain aversion circuits during withdrawal (negative reinforcement) (Kenny and Markou, 2001). Consistent with a key role in tobacco use disorder, nicotine is volitionally self-administered via intravenous infusions by humans (Harvey et al., 2004), nonhuman primates (Goldberg and Spealman, 1982), dogs (Risner and Goldberg, 1983), rats (Corrigall and Coen, 1989; DeNoble and Mele, 2006), and mice (Fowler and Kenny, 2011). Nicotine self-administration under fixed-ratio schedules of reinforcement produces an inverted U-shaped dose-response curve similar to other reinforcing drugs, such as opioids and cocaine. The shape of the nicotine dose-response curve reflects competing positive and negative effects of nicotine at different doses. Increased responding for nicotine over the ascending portion of the dose-response curve reflects the intensifying rewarding effects of nicotine as the amount of drug per infusion increases. Decreased responding over the descending portion of the curve likely reflects increasing aversive properties of nicotine that motivate avoidance behaviors. Consistent with the notion that nicotine elicits both reinforcing and punishing effects is the observation that nonhuman primates volitionally self-administer the same doses of nicotine that they will work to avoid when they are delivered nonvolitionally (Goldberg and Spealman 1982, 1983; Spealman and Goldberg, 1982). Factors other than aversion also contribute to the descending arm of the dose-response curve seen in animals responding under fixed-ratio schedules. For example, more rapid “satiation” when higher doses are available, which means that lower rates of responding can be sustained yet still achieve the same pharmacological actions as lower drug doses, can contribute to the descending arm of the curve (Fowler and Kenny, 2011). Similarly, disruption of behavioral performance also contributes to decreased responding when higher drug doses are self-administered (Fowler and Kenny, 2011). Obtaining the rewarding effects of nicotine while avoiding its aversive and performance-disrupting effects is thought to play a major role in determining patterns and amounts of tobacco intake in smokers (Fowler and Kenny, 2014). Moreover, nicotine-induced adaptive responses in the brain systems that regulate nicotine reward and aversion likely regulate the establishment and maintenance of the tobacco habit with genetic variation that influences these processes, rendering individuals more or less sensitive to the abuse liability of tobacco products (Jensen et al., 2015). Neuronal nicotinic acetylcholine receptors (nAChRs) are the major substrates in the brain for the rewarding and aversive actions of nicotine. The precise molecular, cellular, and circuit-level mechanisms through which different nAChR subtypes regulate these properties of nicotine are not yet fully characterized, but important new insights into these processes have emerged in recent years. Here, we summarize some of the most recent findings on the mechanisms of nicotine reward and aversion and the role for nAChR subtypes in these processes.

### B. Structural Architecture of nAChRs

**Fig. 1:**
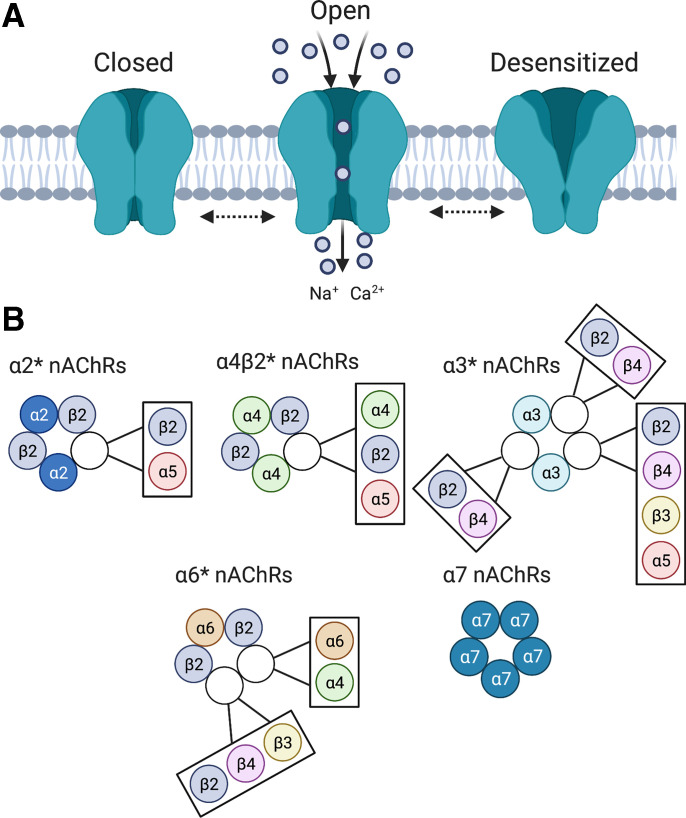
Structural organization nicotinic acetylcholine receptors. (A) nAChRs are pentameric ligand-gated cationic channels. nAChR agonists, such as acetylcholine and nicotine, stabilize the receptor in the active confirmation associated with an open transmembrane pore permeable to calcium sodium (Na^+^), potassium (K^+^), and (Ca^2+^) ions. Prolonged stimulation of the receptor by agonists, such as nicotine, can drive the receptor into an inactive “desensitized” state. (B) Major stoichiometries of homopentameric or heteropentameric nAChRs expressed in the mammalian brain. The open white circles indicate that one of the adjacent subunits contained within the adjacent box are often incorporated into that nAChR subtype.

Neuronal nAChRs are comprised of five distinct membrane-spanning subunits (*α* and *β*) that combine to form a functionally mature pentameric receptor complex (Hucho and Changeux, 1973; Deneris et al., 1991; Sargent, 1993; Albuquerque et al., 1995; Lena and Changeux, 1998). The mature receptor pentamer functions as an allosteric complex that can assume inactive, active, or desensitized confirmational states (Changeux and Taly, 2008), with the cognate agonist acetylcholine or other agonists, such as nicotine, stabilizing the active confirmation associated with an open inner transmembrane cationic channel (Fig. 1). Nicotine and other exogenous agonists can also drive nAChRs into a desensitized state (Changeux, 1979; Changeux et al., 1984) with the propensity to enter and exit desensitization related to the subunit composition of the receptor complex and influenced by various post-translational modifications to this complex. The neuronal *α* subunit exists in nine isoforms (*α*2–*α*10), whereas the neuronal *β* subunit exists in three isoforms (*β*2–*β*4) (Elgoyhen et al., 1994, 2001; Le Novere et al., 2002). Assembly of nAChR subunits into a mature receptor is a tightly regulated process that requires appropriate subunit interactions, with only certain subunit stoichiometries able to generate functional nAChRs. The molecular mechanisms that control assembly of nAChR subunits into mature receptor complexes are poorly understood (Gotti et al., 2009), but modern genome-wide screening approaches are beginning to reveal the cellular components and processes involved (Gu et al., 2019). The major stoichiometries of nAChRs found in the mammalian brain are summarized in Fig. 1. Briefly, homomeric nAChRs are comprised of five *α*7, *α*8, or *α*9 subunits, whereas heteromeric nAChRs contain both *α* and *β* subunits. Heteromeric nAChRs contain agonist binding sites at the interface between an *α* and *β* subunit (Changeux, 1979; Changeux et al., 1984; Gotti et al., 2007). Nicotinic receptors containing *α*4 and *β*2 subunits are denoted as *α*4*β*2* nAChRs, with the asterisk signifying that the nAChR contains other unidentified subunits. *α*4*β*2* nAChRs are the most abundant subtype in the mammalian central nervous system (CNS) and account for the high-affinity nicotine binding sites in the brain (Flores et al., 1992). Nicotinic receptors containing *α*3 and *β*4 subunits (*α*3*β*4* nAChRs) are abundantly expressed by neurons in the autonomic nervous system and are sometimes referred to as the “ganglionic” nAChRs (Kemp and Morley, 1986). *α*5 subunits do not contain an agonist binding domain and thus do not form functional homomeric channels or heteromeric channels when coexpressed with *β*2 or *β*4 nAChR subunits and instead serve as “accessories” that modify the pharmacology of the receptor complexes into which they incorporate (Ramirez-Latorre et al., 1996; Gotti et al., 2009). *β*3 also functions as an accessory subunit (Gotti et al., 2009) and plays a particularly important role in facilitating the assembly and function of *α*6* nAChRs. The role of nAChR subtypes comprised of these different subunits in regulating the behavioral actions of nicotine is reviewed below.

## II. Dopamine Mechanisms of Nicotine Reward

### A. nAChRs in Brain Dopamine Systems

nAChRs in the CNS are located primarily on presynaptic terminals (Wonnacott, 1997) but can also be found at somatodendritic and postsynaptic locations (Clarke et al. 1986; Sargent, 1993). Atypically, nAChRs are also found on the axons of medial habenula (mHb) neurons that comprise the fasciculus retroflexus tract (Clarke et al., 1986; Mulle et al., 1991; Grady et al., 2009; Passlick et al., 2018). The major function of nAChRs in the CNS is the modulation of neurotransmitter release. Accordingly, nicotine stimulates the release of many transmitters in addiction-relevant regions of the brain and enhances neurotransmission in ventral tegmental area (VTA) and nucleus accumbens (NAc), which have been heavily implicated in nicotine reinforcement processes (Schwartz et al., 1984; Carboni et al., 2000b; Mansvelder and McGehee, 2000; Fu et al., 2003). nAChRs are expressed by midbrain dopamine neurons (Clarke and Pert, 1985), and what it has in common with other major drugs of abuse nicotine is that is enhances dopamine release in the NAc of rodents and human smokers (Imperato et al., 1986; Di Chiara and Imperato, 1988; Marshall et al., 1997; Johnson et al., 2000; Brody et al., 2004, 2006, 2009). This action is thought to reflect the ability of nicotine to stimulate VTA dopamine neurons (Pidoplichko et al., 1997) and increase their burst patterns of firing (Grenhoff et al., 1986; De Biasi and Dani, 2011). The stimulatory actions of nicotine on mesoaccumbens dopamine transmission were thought to exclusively reflect its actions at nAChRs located in the VTA (Nisell et al., 1994a,b). However, recent findings have highlighted the contributions of nAChRs expressed on the terminals of dopamine neurons in the striatum (Schwartz et al., 1984; Rice and Cragg, 2004; Cachope et al., 2012; Threlfell et al., 2012). As reviewed in detail below, the majority of studies investigating the mechanisms of nicotine reward have focused on the role of mesoaccumbens dopamine neurons in this process. However, it is important to note that nicotine elicits rewarding effects through actions at nAChRs located outside the mesocorticolimbic dopamine system. Indeed, nicotine is self-administered directly into the central linear nucleus and supramammillary nucleus by rats (Ikemoto et al., 2006), yet the mechanisms underlying the reinforcing actions in these sites and the link between these sites and the stimulatory effects of nicotine on VTA dopamine neurons have not been explored. The rewarding effects of nicotine are also blunted in genetically modified mice in which genes encoding *μ* opioid receptors, opioid peptides, or other related neuropeptide genes have been null mutated, hinting at dopamine-independent mechanisms of nicotine reward (Berrendero et al., 2002, 2005; Walters et al., 2005; Galeote et al., 2009; Sakoori and Murphy, 2009; Trigo et al., 2009; Neugebauer et al., 2011). Nevertheless, remarkably little is known about the role of nAChRs in dopamine-independent mechanisms of nicotine reward.

Nicotine enhances dopamine transmission more robustly in the shell region of the NAc compared with the NAc core region (Nisell et al., 1997; Lecca et al., 2006), with the NAc shell thought to play an important role in nicotine reward (Iyaniwura et al., 2001; Sellings et al., 2008). Dopamine receptor antagonists block the lowering effects of nicotine on intracranial self-stimulation (ICSS) thresholds (Huston-Lyons et al., 1993; Ivanova and Greenshaw, 1997), which reflects the direct stimulatory effects of nicotine on brain reward systems (Wise et al., 1992). Lesioning of midbrain dopamine neurons using the toxin 6-hydroxydopamine abolished the locomotor-stimulating properties of nicotine (Clarke et al., 1988) and reduced intravenous nicotine self-administration in rats (Corrigall et al., 1992; Singer and Wallace, 1984). Blockade of dopamine receptors also reduced nicotine self-administration in rats (Corrigall and Coen, 1991; Corrigall et al., 1992) and attenuated nicotine-induced conditioned place preference (CPP) (Acquas et al., 1989; Spina et al., 2006). These findings suggest that dopamine transmission plays a key role in the motivational properties of nicotine. Notably, however, dopamine receptor and nAChR antagonists increased tobacco consumption in human cigarette smokers (Nemeth-Coslett et al., 1986; Dawe et al., 1995), which is opposite to the effects of such manipulations on nicotine self-administration in rats (Watkins et al., 1999). The reason for this discrepancy is unclear but likely reflects the fact that rodents that self-administer nicotine for a few weeks are less dependent on nicotine than smokers who have been exposed to nicotine for many months or years. Whatever the underlying mechanisms, these findings support an important role for midbrain dopamine neurons, and the nAChRs that regulate mesoaccumbens dopamine transmission, in regulating the motivational properties of nicotine that drive the tobacco habit in human smokers (Corrigall et al., 1992; Fu et al., 2000; Grillner and Svensson, 2000; Tapper et al., 2004; David et al., 2006; Ikemoto et al., 2006).

### B. β2* nAChR Subtypes and Nicotine Reward

**Fig. 2:**
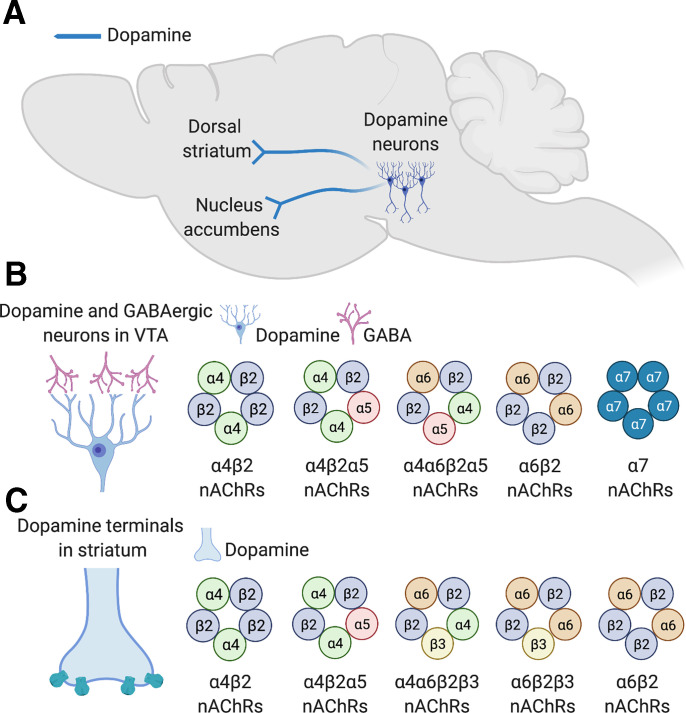
nAChR subtypes in brain dopamine systems. (A) Ventral midbrain dopaminergic neurons that project to the dorsal striatum or nucleus accumbens are stimulated by nicotine, resulting in increased dopamine transmission in the striatum. (B) Major stoichiometries of nAChRs predicted to be expressed by dopaminergic and GABAergic neurons in the ventral tegmental area. (C) Major stoichiometries of nAChRs predicted to be expressed by on the terminals of dopaminergic in the dorsal striatum and nucleus accumbens.

*α*4*β*2* nAChRs are the most abundant subtype in the mammalian CNS (Flores et al., 1992). *α*4*β*2 nAChRs occur in two discrete stoichiometries: (*α*4*β*2)_2_*β*2 or (*α*4*β*2)_2_*α*4 subtypes (Nelson et al., 2003; Gotti et al., 2009). (*α*4*β*2)_2_*β*2 nAChRs are far more sensitive to agonist-induced activation (EC_50_ ∼1 mM for acetylcholine) than the (*α*4*β*2)_2_*α*4 subtype (EC_50_ ∼100 mM) (Gotti et al., 2009). However, (*α*4*β*2)_2_*β*2 nAChRs are also far more sensitive to agonist-induced desensitization than (*α*4*β*2)_2_*α*4 nAChRs (Gotti et al., 2009). In addition to (*α*4*β*2)_2_(*α*4) and (*α*4*β*2)_2_(*β*2) nAChRs, several other *β*2* nAChR stoichiometries exist in the brain and are involved in regulating behavioral responses to nicotine. For example, *β*2* nAChRs can incorporate *α*2 or *α*6 subunits in some regions of the brain (Zoli et al., 2002; Salminen et al., 2004; Grady et al., 2009; Gotti et al., 2010). Likewise, *β*4* nAChRs can also incorporate *α*2 and *α*6 subunits (Zoli et al., 2002; Salminen et al., 2004; Gotti et al., 2009; Grady et al., 2009; Azam et al., 2010; Dash and Li, 2014). Using polymerase chain reactions (PCRs) to assess nAChR subunit expression in animals after unilateral lesion of VTA dopamine neurons, it was found that mRNA transcripts for *α*2, *α*3, *α*5, *α*6, *α*7 and *β*4 subunits were downregulated in the lesioned hemisphere compared with the intact side (Charpantier et al., 1998). By contrast, mRNA for *α*4, *β*2 and *β*3 subunits was detected after the lesion of dopamine neurons (Charpantier et al., 1998). This suggests that *α*2, *α*3, *α*5, *α*6, *α*7, and *β*4 nAChR subunits are expressed by VTA dopamine neurons that project to the NAc, whereas *α*4, *β*2, and *β*3 subunits are expressed by nondopamine cells in the VTA. Using PCR and single-cell electrophysiological recordings, almost 100% of dopamine and nondopamine neurons in the VTA were shown to express mRNA for the *α*4 subunit (Klink et al., 2001), whereas ∼90% of dopamine neurons and 20% of GABA neurons expressed *β*3 nAChR subunits (Klink et al., 2001). Further, 70%–75% of VTA dopamine neurons expressed *α*5 and *α*6 subunit mRNAs, but a much lower proportion of GABAergic cells (10%–20%) expressed these subunit transcripts (Klink et al., 2001). A similar proportion of VTA dopamine and GABA neurons (∼40%) expressed *α*7 nAChR subunit transcripts (Klink et al., 2001). *β*4 mRNA was detected only at low concentrations in VTA cells. Based on these and related findings, it was proposed that three major subtypes of nAChRs are expressed by VTA dopamine neurons: (*α*4*β*2)(*α*6*β*2)(*α*5), (*α*4*β*2)_2_(*α*5), and *α*7 (Fig. 2). On VTA GABAergic neurons, it was proposed that two nAChR subtypes predominate: (*α*4*β*2)_2_(*α*4) and *α*7 nAChRs (Fig. 2). Notably, *β*3* nAChRs are not thought to exist in the VTA despite the high concentrations of *β*3 mRNA expressed by dopamine neurons (Klink et al., 2001). This is because *β*3 subunits are transported to the terminal regions to which VTA neurons project (Forsayeth and Kobrin, 1997), with high *β*3 protein concentrations detected in striatum but not VTA (Forsayeth and Kobrin, 1997; Reuben et al., 2000; Salminen et al., 2004). Hence, *β*3 nAChR subunits are likely to be incorporated exclusively into the nAChRs on the terminals of VTA neurons in the striatum and elsewhere in the brain, where they regulate the stimulatory effects of nicotine on dopamine transmission (Klink et al., 2001). Immunoprecipitation, ligand-binding, genetic deletion, and targeted lesion studies support the existence of at least four species of nAChRs on the terminals of VTA dopamine neurons in striatum: (*α*4*β*2)_2_(*α*4), (*α*4*β*2)2(*α*5), (*α*4*β*2)(*α*6*β*2)*β*3, (*α*6*β*2)_2_*β*2, and (*α*6*β*2)_2_*β*3 (Zoli et al., 2002; Salminen et al., 2004) (Fig. 2). In addition to dopamine neurons, GABA neurons in the VTA also express *α*6*β*2* nAChRs (Yang et al., 2011). There is evidence that *α*6 can occasionally assemble into *β*4* nAChRs, with *α*6*β*4* nAChRs involved in regulating norepinephrine release in the hippocampus (Azam et al., 2010) (Fig. 2). However, *α*6*β*4* are thought to be minimally involved in regulating mesoaccumbens dopamine transmission (Azam et al., 2010). It has been speculated that each dopamine neuron in the VTA expresses only one particular nAChR subtype, meaning that these cells can be functionally categorized based on their nAChR expression patterns, with the particular nAChR subtype expressed by each class of dopamine neuron determining their responses to nicotine (Yang et al., 2009).

**Fig. 3:**
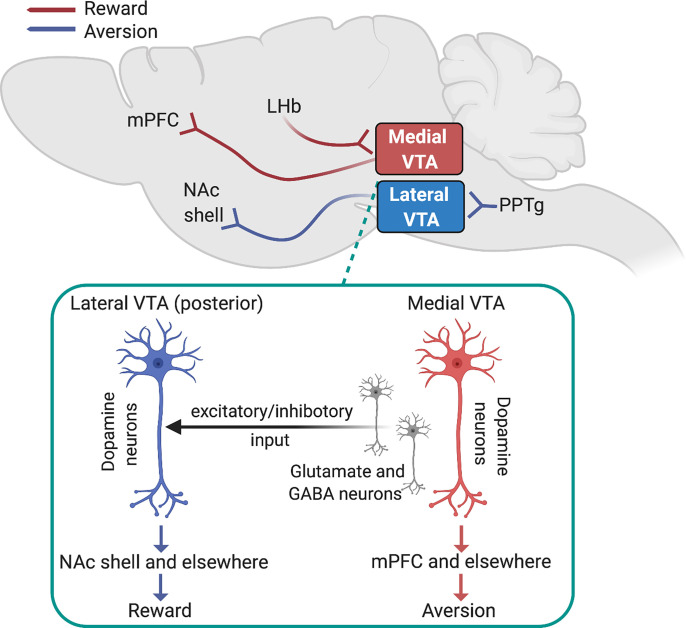
Dopamine mechanisms of nicotine reward and aversion. Dopamine neurons located in the medial VTA that receive input from the LHb and project to the mPFC regulate aversion-related behaviors. Dopamine neurons located in the lateral VTA that receive input from the PPTg and project to the medial portion of the NAc shell regulate reward-related behaviors. Shown in the insert is the putative nAChR-regulated excitatory and inhibitory input from the medial VTA to the lateral VTA, particularly in posterior (caudal) VTA, that may regulate reward-related responses to nicotine.

*α*4*β*2* nAChRs are the major subtype responsible for the stimulatory effects of nicotine on midbrain dopamine neurons (Subramaniyan and Dani, 2015; Thiruchselvam et al., 2017). Nicotine-induced burst firing of midbrain dopamine neurons is abolished in mice lacking the *β*2 nAChR subunit (Mameli-Engvall et al., 2006), resulting in attenuated striatal dopamine release in response to nicotine treatment (Picciotto et al., 1998). Accordingly, *β*2 subunit knockout mice are almost entirely insensitive to the reinforcing properties of nicotine (Picciotto et al., 1998), with this effect attributed to the diminished stimulatory actions of nicotine on VTA dopamine neurons (Besson et al., 2006; Pons et al., 2008; Orejarena et al., 2012; Grieder et al., 2019; Bagdas et al., 2019). Nicotine stimulates VTA dopamine neurons via *β*2* nAChRs located directly on these cells (Durand-de Cuttoli et al., 2018). Nicotine also stimulates populations of *β*2* and *α*7 nAChRs on the terminals of glutamatergic inputs to VTA dopamine neurons, resulting in prolonged increases in excitatory transmission in the VTA (Yan et al., 2018; Mansvelder and McGehee, 2000). In parallel, nicotine activates then rapidly desensitizes *β*2* nAChRs expressed by VTA GABAergic neurons (Mansvelder et al., 2002; Yang et al., 2011). These findings suggest that the net action of nicotine is an increase in the ratio of excitatory to inhibitory transmission onto VTA dopamine neurons (Durandde Cuttoli et al., 2018), with *β*2* nAChRs critical for these actions. Indeed, virus-mediated re-expression of the *Chrnb2* gene, which encodes the *β*2 nAChR subunit, in the VTA of *β*2 nAChR subunit knockout mice restored the ability of nicotine to evoke burst firing of dopamine neurons in these animals (Maskos et al., 2005; Mameli-Engvall et al., 2006; Naude et al., 2016). *Chrnb2* re-expression in the VTA also restored the reinforcing and locomotor-stimulating properties of nicotine in *β*2 knockout mice (Maskos et al., 2005). However, a more recent study showed that virus-mediated *Chrnb2* re-expression in the midbrain dopamine system of *β*2 knockout mice did not reinstate their sensitivity to nicotine in a CPP procedure but did reinstate sensitivity to the locomotor-stimulating effects of nicotine (Avale et al., 2008; Mineur et al., 2009). These findings could reflect different components of the midbrain dopamine system being involved in regulating discrete behavioral responses to nicotine, with these components differentially targeted across studies. Alternatively, it is possible that discrete populations of cells within the VTA regulate behavioral responses to nicotine, with the virus vectors or other factors used across different studies differentially impacting these cell populations. Consistent with both possibilities, nicotine is self-administered only into posterior but not anterior regions of VTA by rats (Ikemoto et al., 2006), and nicotine modulates the balance between excitatory and inhibitory drive onto VTA dopamine neurons in a manner that depends on their precise location within the VTA (Yan et al., 2019). Furthermore, *Chrnb2* re-expression concurrently in both VTA dopamine and GABAergic neurons was required to rescue the sensitivity of *β*2 subunit knockout mice to the actions of nicotine in CPP and self-administration procedures (Tolu et al., 2013; Grieder et al., 2019). Hence, reward-related behavioral responses to nicotine are likely parsed into discrete anatomic regions of the VTA, and within these regions many different classes of neurons are likely to participate. This concept of spatial and cellular segregation of nicotine reward within the VTA is considered in more detail below.

Systemic or intra-VTA administration of the *β*2* nAChR antagonist dihydro-*β*-erythroidine (DH*β*E) decreased nicotine self-administration in rats (Corrigall and Coen, 1989; Watkins et al., 1999). In addition, DH*β*E reduced the stimulatory effects of nicotine on brain reward systems, as shown by attenuated nicotine-induced lowering of ICSS thresholds in rats (Ivanova and Greenshaw, 1997; Harrison et al., 2002). As DH*β*E is relatively selective for *β*2* nAChRs compared with other classes of nAChRs (Harvey and Luetje, 1996; Harvey et al., 1996), these findings further implicate this nAChR subtype in the motivational properties of nicotine. The novel nAChR compound SSR591813, considered a partial agonist at *α*4*β*2* nAChRs, decreased nicotine self-administration in rats (Cohen et al., 2003). Similarly, the novel nAChR compound UCI-30002, a partial agonist at *α*4*β*2* nAChRs, also decreased nicotine self-administration in rats (Yoshimura et al., 2007), whereas 5-iodo-A-85380, a putative agonist at *β*2* nAChRs, was actively self-administered by rats (Liu et al., 2003). Varenicline is a partial agonist at *α*4*β*2* nAChRs but a full agonist at *α*7 nAChRs (Coe et al., 2005; Mihalak et al., 2006; Lerman et al., 2007; Reus et al., 2007; Dwoskin et al., 2009) and can compete with nicotine for binding sites on *α*4*β*2* nAChRs in the VTA and elsewhere in the brain. In this manner, varenicline can attenuate the stimulatory action of nicotine on dopamine transmission (Coe et al., 2005; Reperant et al., 2010). Varenicline dose-dependently decreased nicotine self-administration in rats (Rollema et al., 2007; O'Connor et al., 2010), with this action thought to directly reflect the ability of varenicline to attenuate the stimulatory actions of nicotine on VTA dopamine neurons (Harmey et al., 2012). Cytisine and dianicline are both structurally related to varenicline and similarly act as partial agonists at *α*4*β*2* nAChRs (Coe et al., 2005). These compounds have shown clinical utility as smoking cessation medications in humans (Barlow and McLeod, 1969; Reavill et al., 1990; Etter, 2006; Rollema et al., 2010). Bupropion has been shown to antagonize *α*4*β*2* nAChRs (Alkondon and Albuquerque, 2005), whereas nicotine replacement therapy is thought to facilitate smoking cessation by stimulation *α*4*β*2* nAChRs in the midbrain (Harmey et al., 2012). Hence, currently available smoking cessation therapeutics have at least some actions at *α*4*β*2* nAChRs. Together, these findings support a major role for *α*4*β*2* nAChRs located in midbrain dopamine systems in regulating the reinforcing properties of nicotine.

Nicotine-induced upregulation of nAChR expression has been detected in the brains of rodents and human smokers (Marks et al., 1983; Breese et al., 1997; Staley et al., 2006; Nashmi et al., 2007). nAChR upregulation is thought to modify the subsequent actions of nicotine on brain reward systems and thereby contribute to the development of nicotine dependence (Esterlis et al., 2014, 2016). Indeed, *β*2* nAChRs expressed by VTA GABAergic neurons are highly sensitive to nicotine-induced upregulation, with this effect correlated with increased sensitivity to the rewarding properties of the drug (Nashmi et al., 2007; Ngolab et al., 2015). Positron emission tomographic imaging of the brains of smokers using a radiotracer to measure densities of *α*4*β*2* nAChRs has shown that greater levels of nAChR upregulation are associated with greater difficulty in achieving and maintaining abstinence from tobacco use (Brody et al., 2014). The fact that *β*2* nAChRs expressed by VTA GABAergic neurons are so sensitive to nicotine-induced upregulation suggests that adaptive responses in VTA GABAergic transmission may play a particularly important role in the development of nicotine dependence. Intriguingly, farnesol, which is often added to nicotine contained in electronic delivery systems to improve its flavor, can increase the activity of VTA dopamine neuron while decreasing the activity of local GABAergic neurons in a manner similar to nicotine (Avelar et al., 2019). Menthol, which is also incorporated into cigarettes to modify their flavor (Ai et al., 2016), also modifies the function of *β*2* nAChRs in the VTA to enhance the rewarding effects of nicotine (Henderson et al., 2017). This suggests that flavorants added to cigarettes and electronic smoking devices may facilitate nicotine use not only by masking the noxious bitter taste of nicotine but by directly modifying the receptor and cellular substrates in the VTA upon which nicotine acts.

### C. α4* nAChR Subtypes and Nicotine Reward

The most common stoichiometry of *β*2* heteromeric nAChRs is thought to incorporate two *α*4 and three *β*2 subunits, which is denoted as (*α*4*β*2)_2_(*β*2), and contains orthosteric binding sites for acetylcholine, nicotine, and other agonists at the two *α*4/*β*2 interfaces (Zwart and Vijverberg, 1998; Albuquerque et al., 2009). In a less common stoichiometry denoted as (*α*4*β*2)_2_(*α*4), one of the *β*2 subunits is substituted with an *α*4 subunit (Zwart and Vijverberg, 1998; Mazzaferro et al., 2011). This incorporates a third “unorthodox” agonist binding site at the *α*4/*α*4 interface (Mazzaferro et al., 2011). As noted above, the (*α*4*β*2)_2_(*α*4) nAChR stoichiometry has lower affinity for nicotine than the (*α*4*β*2)_2_(*β*2) subtype (Moroni et al., 2006; Campling et al., 2013). However, the unorthodox binding site in the (*α*4*β*2)_2_(*α*4) nAChR stoichiometry results in 3- to 4-fold greater levels of receptor activation by acetylcholine or nicotine when compared with the (*α*4*β*2)_2_(*β*2) stoichiometry (Moroni et al., 2006; Timmermann et al., 2012; Wang et al., 2015; Jain et al., 2016). Hence, the (*α*4*β*2)_2_(*β*2) and (*α*4*β*2)_2_(*α*4) subtypes can be considered “high-affinity” and “high-efficacy” nAChRs, respectively. Both of these nAChR stoichiometries are thought to be expressed by neurons in the adult mammalian brain (Marks et al., 2007; Gotti et al., 2008), and pharmacological agents have been identified or developed that can discriminate between them (Moroni et al., 2006). The smoking-cessation therapeutics varenicline and cytisine are partial agonists at (*α*4*β*2)_2_(*α*4) nAChRs but are inactive at (*α*4*β*2)_2_(*β*2) nAChRs (Moroni et al., 2006; Campling et al., 2013). In addition, the (*α*4*β*2)_2_(*α*4) nAChR positive allosteric modulator NS9283 (Mazzaferro et al., 2019) decreased nicotine self-administration in rats (Maurer et al., 2017). NS9283, cytisine, and varenicline also decreased ethanol intake in rats (Steensland et al., 2007; Bell et al., 2009; Sotomayor-Zarate et al., 2013; Wang et al., 2020). Hence, (*α*4*β*2)_2_(*α*4) nAChRs likely play an important role in the reinforcing properties of nicotine and other drugs of abuse, and compounds that modulate this nAChR stoichiometry may serve as novel therapeutics for substance use disorders (see Fig. 2).

Similar to *β*2 nAChR subunit knockout mice (Walters et al., 2006), deletion of *α*4 nAChR subunits in the ventral midbrain blocked nicotine-induced CPP in mice (Peng et al., 2017). Conditional deletion of *α*4 nAChR subunits from dopamine neurons in mice similarly blocked nicotine-induced CPP (McGranahan et al., 2011). Conversely, mutant mice expressing a hypersensitive *α*4 nAChR subunit are hyper-responsive to nicotine reward, as reflected by the establishment of CPP for very low doses of nicotine that have no detectable effects in wild-type mice (Tapper et al., 2004). The hypersensitive *α*4 mutant mice also showed increased sensitivity to the stimulatory effects of nicotine on midbrain dopamine neurons (Tapper et al., 2004). Using a behavioral procedure in which nicotine is self-administered via the tail vein during a single session, it has been shown that *β*2 and *α*4 subunit knockout mice but not *α*7 knockout mice consume markedly less nicotine than their wild-type counterparts (Pons et al., 2008). Lentivirus-mediated re-expression of the *β*2 or *α*4 subunit genes in the VTA but not substantia nigra of the respective knockout mice “rescued” their nicotine intake in this acute self-administration procedure (Pons et al., 2008). However, *α*4 nAChR subunit knockout mice did not show any difference in nicotine intake using a more traditional chronic intravenous (jugular catheter) self-administration procedure (Cahir et al., 2011). However, they did show attenuated locomotor suppression in response to injection of a relatively high dose of nicotine (Cahir et al., 2011), a behavioral response thought to reflect the aversive actions of nicotine (Morrison and Stephenson, 1972; Stolerman et al., 1973; Clarke and Kumar, 1983a,b; Hentall and Gollapudi, 1995; Salas et al., 2004a; Frahm et al., 2015; Antolin-Fontes et al., 2020). Hence, it is possible that *α*4* nAChRs play a more prominent role in nicotine aversion than nicotine reward at least under the testing conditions in these experiments (discussed in more detail below) (see Fig. 2).

### D. α6* nAChR Subtypes and Nicotine Reward

Much interest has centered on the potential involvement of *α*6* nAChR subunits in nicotine reward processes. This interest has arisen in large part because of the high concentrations and restricted patterns of expression of mRNA transcripts for *α*6 subunits within the VTA and other catecholaminergic nuclei of the brain (Le Novere et al., 1996; Quik et al., 2000; Azam et al., 2002; Champtiaux et al., 2002; Gotti et al., 2006a). Non-*α*4(*α*6*β*2)_2_* nAChRs are abundantly expressed by mesoaccumbens dopamine neurons, whereas (*α*6*β*2)(*α*4*β*2)* nAChRs are expressed by nigrostriatal dopamine neurons (Gotti et al., 2010). The stimulatory effects of nicotine on VTA dopamine neurons were attenuated *α*6 nAChR subunit knockout mice (Liu et al., 2012). *α*6 nAChR subunits were also upregulated in rats by chronic intravenous nicotine self-administration (Parker et al., 2004) in both dopaminergic and GABAergic neurons in the VTA (Akers et al., 2020). The magnitude by which (*α*6*β*2)(*α*4*β*2)* nAChRs were upregulated in the VTA of mice corresponded to their sensitivity to the rewarding effects of nicotine (Akers et al., 2020). However, it was previously reported that *α*6* nAChRs were downregulated by chronic nonvolitional nicotine treatment through a process influenced by the presence or absence of *β*3 nAChR subunits (Lai et al., 2005; Mugnaini et al., 2006; Perry et al., 2007; Marks et al., 2014). Hence, the volitional nature of nicotine delivery and their precise subunit composition likely determine whether *α*6* nAChRs are upregulated or downregulated by nicotine. *α*6*β*2* nAChRs are activated by cytisine and varenicline far more efficiently than other *β*2* nAChRs (Salminen et al., 2004; Bordia et al., 2012), suggesting that they may contribute to the clinical utility of these compounds as smoking-cessation therapeutics. Furthermore, allelic variation in the *CHRNB3-CHRNA6* gene cluster on chromosome 8, which encodes the *β*3 and *α*6 nAChR subunits, respectively, increases vulnerability to tobacco dependence (Bierut et al., 2007; Thorgeirsson et al., 2010; Wen et al., 2016). Behavioral data have accumulated to support a role for *α*6* nAChRs in nicotine reinforcement, but their involvement is complex, and their precise contributions remain unclear. *α*6 nAChR subunit knockout mice did not drink a nicotine-containing solution in a two-bottle choice procedure (Bagdas et al., 2019). Similarly, *α*6 nAChR subunit knockout mice do not self-administer nicotine using an acute tail-vein procedure during a single session (Pons et al., 2008). Lentivirus-mediated re-expression of the *α*6 subunit in the VTA of the knockout mice re-established their sensitivity to nicotine reinforcement in this procedure (Pons et al., 2008). Transgenic mice expressing a gain-of-function *α*6 nAChR mutant subunit showed enhanced sensitivity to the stimulatory effects of nicotine on striatal dopamine transmission and increased locomotor stimulant responses to nicotine (Drenan et al., 2010), with these effects attributed to increased function of (*α*6*β*2)(*α*4*β*2)* nAChRs (Drenan et al., 2010; Engle et al., 2013). Pharmacological blockade of *α*6* nAChRs in the VTA or NAc of rodents abolished the stimulatory effects of nicotine on dopamine transmission in the striatum and decreased nicotine self-administration behavior (Brunzell et al., 2010; Gotti et al., 2010; Brunzell, 2012; Sanjakdar et al., 2015), with the *α*6*β*2* nAChR subtype hypothesized to play a prominent role in these effects (Whiteaker et al., 2000; Marks et al., 2014). Indeed, the (*α*4*β*2)(*α*6*β*2)*β*3 nAChR subtype has the highest sensitivity to nicotine of any native nAChR so far identified (Grady et al., 2007). In addition, the novel nAChR antagonist bPiDDB (*N*, *N*′-dodecane-1,12-diyl-*bis*-3-picolinium dibromide) dose-dependently decreased nicotine self-administration in rats and attenuated the locomotor-stimulating effects of acute and repeated nicotine treatment (Neugebauer et al., 2006). The related *α*6* antagonist (*N*, *N*-decane-1,10-diyl-*bis*-3-picolinium diiodide) bPiDI decreased intravenous nicotine self-administration in wild-type mice and in mice carrying a mutation in the *α*4 nAChR subunit that renders it insensitive to mecamylamine and other nAChR antagonists (Madsen et al., 2015). Considering that *α*4 nAChR subunit knockout mice intravenously self-administer nicotine at similar levels to wild-type mice (Cahir et al., 2011), it was proposed that *α*6*β*2* nAChRs are likely to be the critical subtype that regulates the reinforcing properties of nicotine (Madsen et al., 2015); for review, see (Brunzell 2012).

However, recent findings have raised questions about the degree to which *α*6* nAChRs contribute to the reinforcing properties of nicotine. *α*4* but not *α*6* nAChRs regulate nicotine-induced bursting of VTA dopamine neurons (Exley et al., 2011). The novel *α*6*β*2* nAChR agonist TC299423 induced only modest rewarding effects in wild-type, which were enhanced in *α*6 gain-of-function mutant mice (Wall et al., 2017). However, TC299423 had no-effects, no-intravenous-nicotine self-administration in rats (Wall et al., 2017). Using an intra-VTA self-administration procedure, it was shown that *α*6 nAChR subunit knockout mice will self-administer similar quantities of nicotine in the VTA as wild-type mice, whereas *α*4 subunit knockout mice self-administer far less nicotine (Exley et al., 2011). This suggests that *α*6* nAChRs located in the VTA are unlikely to regulate the reinforcing actions of nicotine. One explanation to reconcile these discrepant findings is that *α*4* nAChRs may dominate the local actions of nicotine in the VTA, whereas *α*6* nAChRs located on the terminals of dopamine neurons may regulate the local actions of nicotine in the striatum (Exley et al., 2011). Indeed, both *α*4* and *α*6* contribute to the stimulatory effects of nicotine on dopamine release in the striatum (Cui et al., 2003; Salminen et al., 2004, 2007; Grady et al., 2007, 2010), with *α*4*β*2* and (non-*α*4)(*α*6*β*2)_2_* nAChRs located on the terminals of dopamine neurons regulating the actions of nicotine in the striatum (Exley et al., 2008, 2011) (Fig. 2). If *α*6* nAChRs only regulate the actions of nicotine in the striatum and not in the VTA, then nicotine may modulate dopamine release through dissociable actions on dopamine neurons at somatodendritic and terminal brain regions (Reuben et al., 2000). This raises important questions about the function and behavioral significance of dopamine released by nicotine acting at somatodendritic versus terminal locations (discussed in more detail below).

### E. β3* nAChR Subtypes and Nicotine Reward

The *β*3 nAChR subunit gene is located in the same genomic locus as the *α*6 subunit gene, and both are thought to be cotranscribed (Moen et al., 2021). Moreover, the *β*3 subunit is known to facilitate the maturation and expression of *α*6* nAChRs (Gotti et al., 2006b, 2009; Drenan et al., 2008), and *β*3 subunit knockout mice demonstrate markedly reduced (∼75% lower) levels of *α*6* nAChRs in the striatum compared with wild-type mice (Gotti et al., 2005). Hence, the *β*3 nAChR subunit can be considered an accessory component of the *α*6* nAChRs that regulate striatal dopamine transmission (Fig. 2). The *β*3 nAChR subunit has received considerable attention as a possible component of the nAChR subtypes that regulate nicotine reward processes. The nAChR antagonist *α*-conotoxin MII partially inhibits nicotine-induced dopamine release from striatal synaptosomes (Kulak et al., 1997; Kaiser et al., 1998). *α*-Conotoxin MII binding in the striatum was shown to depend on the expression of *β*3* and *α*6* nAChRs (Champtiaux et al., 2002; Cui et al., 2003). This has led to the proposal that at least two populations of nAChRs regulate the stimulatory effects of nicotine on dopamine release in the striatum (Kulak et al., 1997) *α*-conotoxin MII–sensitive and –insensitive components. Subsequent studies suggested that the *α*-conotoxin MII–sensitive component of nicotine-evoked dopamine release in striatum requires *β*3* nAChRs that are partially dependent upon *α*4 subunits (Cui et al., 2003; Salminen et al., 2004), likely representing *α*6*β*3*β*2* and *α*4*α*6*β*3*β*2* nAChRs. By contrast, the *α*-conotoxin MII–insensitive component reflects the contributions of *α*4* nAChRs, likely representing *α*4*β*2* and *α*4*β*2*α*5* nAChRs (Salminen et al., 2004). This is consistent with previous work described above, suggesting that mesoaccumbens dopamine neurons express four species of nAChR subtypes on their terminals, two of which contain *β*3 subunits: *α*6*β*3*β*2* and *α*4*α*6*β*3*β*2* nAChRs (Zoli et al., 2002).

### F. α5* nAChR Subtypes and Nicotine Reward

Similar to *β*3, *α*5 nAChR subunits do not reliably form functional nAChRs containing agonist binding sites when coexpressed with *β* subunits (Ramirez-Latorre et al., 1996; Gerzanich et al., 1998; Kuryatov et al., 2008; Dash et al., 2012). Instead *α*5 nAChR subunits are thought to function as accessory subunits in mature nAChR complexes. In the adult mammalian brain, *α*5 subunits are thought to incorporate most efficiently into *α*4*β*2* nAChRs (Perry et al., 2007; Gotti et al., 2007; Kuryatov et al., 2008; Mao et al., 2008). Incorporation of an *α*5 subunit into *α*4*β*2* nAChRs yields the (*α*4*β*2)_2_*α*5 subtype that has the highest known permeability to calcium ions of any nAChR subtype (Ramirez-Latorre et al., 1996; Gotti et al., 2009); for recent review, see (Scholze and Huck, 2020). The presence of an *α*5 subunit can also alter receptor desensitization and upregulation dynamics in response to agonist exposure. In synaptosome and slice physiology preparations from mouse brain, up to 8-fold higher concentrations of nicotine and other agonists were required to desensitize *α*4*β*2* nAChRs that had incorporated an *α*5 subunit (Grady et al., 2012; Poorthuis et al., 2013; Wageman et al., 2014). Allelic variation in *CHRNA5*, the gene that encodes the *α*5 nAChR subunit, is heavily associated with vulnerability to nicotine dependence (Berrettini et al., 2008; Bierut et al., 2008; Hung et al., 2008; Thorgeirsson et al., 2008). In particular, the rs16969968 risk variant in *CHRNA5* increases risk of tobacco dependence gives rise to an amino-acid substitution (398D→398N) in the cytoplasmic domain in an amphipathic *α* helix just preceding the fourth transmembrane domain. This area of the subunit is known to influence channel permeability, particularly to Ca^2+^ ions (Wang et al., 1996, 1998; Gerzanich et al., 1998; Tapia et al., 2007; Kuryatov et al., 2008). This suggests that nAChRs that incorporate the mutant *α*5 nAChR have reduced function, which is consistent with experimental observations (Bierut et al., 2008). *α*5 nAChR subunits are expressed by midbrain dopamine neurons (Azam et al., 2002) and can incorporate into *α*4*β*2* (but not *α*6*) nAChRs to form a functional (*α*4*β*2)_2_*α*5 nAChR subtype in these cells. In the VTA, *α*5 subunits are thought to facilitate the maturation and expression of *α*4*β*2 nAChRs and enhance their function (Chatterjee et al., 2013). As might be expected, incorporation of an *α*5 subunit also increases the resistance of *α*4*β*2 nAChRs in the VTA to agonist-induced desensitization (Chatterjee et al., 2013) and renders *α*4*β*2*α*5* nAChRs in the ventral midbrain largely resistant to nicotine-induced upregulation (Mao et al., 2008).

The (*α*4*β*2)_2_*α*5 nAChR subtype is expressed on the terminals of dopamine neurons in the striatum (Salminen et al., 2004; Mao et al., 2008; Grady et al., 2010; Scholze and Huck, 2020) (Fig. 2), where it serves as an important regulator of the stimulatory effects of nicotine on dopamine transmission. (*α*4*β*2)_2_*α*5 nAChRs regulate dopamine release in the striatum in a manner that can be pharmacologically, functionally, and anatomically dissociated from dopamine release regulated by *α*6*β*2* and (*α*6*β*2)(*α*4*β*2)* nAChRs (Salminen et al., 2004; Grady et al., 2010; Exley et al., 2012). For example, (*α*4*β*2)_2_*α*5 nAChRs regulate the stimulatory effects of nicotine on dopamine release largely in dorsal striatum, whereas *α*6* nAChRs regulate dopamine release in the NAc (Exley et al., 2012). In fact, *α*5* and *α*6* nAChRs on dopamine neurons are thought to be nonoverlapping populations that independently regulate striatal dopamine release (Exley et al., 2012). The functional significance of these different mechanisms of dopamine release is not yet clear but likely reflects dissociable dopamine-related behaviors influenced by nicotine. The direct stimulatory effects of nicotine on VTA dopamine neurons are attenuated in *α*5 subunit knockout mice (Morel et al., 2014; Sciaccaluga et al., 2015) consistent with the attenuated striatal dopamine responses to nicotine in these mice. Virus-mediated re-expression of *α*5 subunits in VTA dopamine neurons of the *α*5 subunit knockout mice can “rescue” their responsiveness to nicotine (Morel et al., 2014; Sciaccaluga et al., 2015), further supporting an important role for (*α*4*β*2)_2_*α*5 nAChRs in the dopamine-enhancing actions of nicotine. In contrast to *α*4*β*2* nAChRs in the midbrain (Akers et al., 2020), (*α*4*β*2)_2_*α*5 nAChR’s system is resistant to nicotine-induced upregulation (Mao et al., 2008), suggesting that adaptive changes in their expression levels do not contribute to the development of nicotine dependence.

### G. α7* nAChR Subtypes and Nicotine Reward

In contrast to the heteromeric nAChRs, behavioral evidence linking *α*7 homomeric nAChRs to the rewarding and reinforcing actions of nicotine is relatively weak. *α*7 nAChRs are thought to contribute to the stimulatory effects of nicotine on excitatory glutamatergic inputs to VTA dopamine neurons (Girod et al., 2000; Mansvelder and McGehee, 2000). Nevertheless, the rewarding effects of nicotine were unaltered in *α*7 nAChR subunit knockout mice compared with wild-type mice in a CPP procedure (Walters et al., 2006). Similarly, the acquisition of nicotine self-administration was unaltered relative to wild-type mice in an acute tail-vein self-administration procedure (Pons et al., 2008). However, oral nicotine intake gradually extinguished over time in *α*7 knockout mice relative to wild-type mice using a two-bottle choice procedure (Levin et al., 2009). Female but not male *α*7 nAChR subunit knockout mice consumed less nicotine that their respective wild-type control groups in a two-bottle choice procedure (Bagdas et al., 2019). Intravenous nicotine self-administration was reduced by the putatively selective *α*7 nAChR antagonist methyllycaconitine (MLA) (Markou and Paterson, 2001). Complicating this finding is the fact that MLA can antagonize non-*α*7 nAChR subtypes (Bryant et al., 2002) and retains nicotine-related behavioral effects in *α*7 subunit knockout mice (Salas et al., 2007). Hence, caution should be exercised when attributing behavioral or physiologic effects of MLA to an action exclusively at *α*7 nAChRs.

### H. Nicotine Modifies Impulse-Dependent and -Independent Accumbal Dopamine Release

The studies exported above often employed in vivo microdialysis or ex vivo brain slice superfusion techniques to investigate the actions of nicotine on mesoaccumbens dopamine transmission (Di Chiara, 2000). Drawbacks of such approaches include the very long sampling times (order of minutes) over large portions of the striatum and often reflect nonsynaptic “spillover” of dopamine that escapes rapid reuptake or breakdown (Parsons and Justice, 1992; Zhou et al., 2001). More complex actions of nicotine on accumbal dopamine transmission have been revealed using more modern approaches that can capture rapid “synaptic” dopamine transmission in the accumbens. Using fast-scan cyclic voltammetry to monitor electrically evoked dopamine release, it was shown that nicotine inhibits action potential-dependent dopamine release in the NAc (Zhou et al., 2001). The nAChR antagonists mecamylamine and DH*β*E mimicked this action of nicotine (Zhou et al., 2001). These data suggest that nicotine acted by desensitizing *β*2* nAChRs located on the terminals of dopamine neurons in the accumbens and hint at complex “multimodal” actions of nAChRs on accumbal dopamine transmission rather than the uniform nicotine-induced increases in dopamine release suggested by previous studies. The inhibitory effect of nicotine on dopamine release measured by voltammetry depends on the baseline activity of dopamine neurons. When dopamine release in the accumbens was stimulated using single electrical pulses to recapitulate tonic-like firing patterns (usually 2–5 Hz), nicotine decreased dopamine release in a manner consistent with the desensitization of presynaptically located *β*2* nAChRs (Rice and Cragg, 2004; Zhang and Sulzer, 2004). However, when dopamine release was stimulated using multiple pulses in a manner that recapitulates burst-like firing patterns (usually 15–100 Hz) thought to occur when rewarding or reward-predictive stimuli are encountered (Schultz, 1986), nicotine instead increased dopamine release (Rice and Cragg, 2004; Zhang and Sulzer, 2004). Based on these findings, it was proposed that nicotine acts as a “high-pass filter” that enhances the contrast between tonic and phasic patterns of dopamine neuron activity, with this action potentially contributing to the reward-enhancing properties of the drug (Rice and Cragg, 2004). Nicotine appeared to act in this manner by blocking short-term inhibitory plasticity in dopamine neurons (Rice and Cragg, 2004), which serves to limit dopamine release during periods of high activity (Cragg, 2003), and instead enhancing short-term calcium-dependent excitatory plasticity (Zhang and Sulzer, 2004).

The population of *β*2* nAChRs in the accumbens that is desensitized by nicotine to enhance activity-dependent dopamine release was shown to contain *α*4, *α*6, and *β*3 subunits (Exley and Cragg, 2008; Exley et al., 2011, 2012). This likely reflects the high-affinity (*α*4*β*2)(*α*6*β*2)*β*3 nAChR subtype. By contrast, *α*4(non-*α*6), *α*6(non-*β*3), and *α*6(non-*α*4) nAChRs play minimal roles in this action of nicotine (Exley et al., 2012). Interestingly, *α*5 subunit knockout mice did not show a desensitization-like enhancement of activity-dependent dopamine release in the accumbens, suggesting that they are not those nAChRs desensitized by nicotine to promote impulse-dependent dopamine release (Exley et al., 2012). However, the knockout mice showed greater sensitivity to the enhancing effects of the *α*3/*α*6* nAChR antagonist *α*-conotoxin MII on activity-dependent dopamine release in the dorsal striatum (Exley et al., 2012), suggesting that *α*5* nAChR deficiency resulted in upregulation in the expression and function of (*α*4*β*2)(*α*6*β*2)*β*3 nAChRs in dorsal striatum, likely to compensate for deficits in the function of (*α*4*β*2)_2_*α*5* nAChRs thought to specialize in regulating dopamine transmission in dorsal striatum (Champtiaux et al., 2002, 2003; Marubio et al., 2003; Salminen et al., 2004; Grady et al., 2010; Exley et al., 2011, 2012) (Fig. 2). The mechanisms by which desensitization of (*α*4*β*2)(*α*6*β*2)*β*3 nAChRs can alleviate short-term inhibitory and promote short-term excitatory plasticity in dopamine neurons to facilitate activity-dependent dopamine release are unclear but appear to involve dopamine D1 receptor signaling (Goutier et al., 2016). It is important to note that squirrel monkeys treated chronically with nicotine (3 weeks of drinking a nicotine-containing solution) showed attenuated accumbal dopamine release in response to low (tonic) or high (phasic) frequency electrical stimulation and had abolished sensitivity the effects of *α*-conotoxin MII on dopamine release (Perez et al., 2012). This raises questions about the nature of nicotine-induced adaptive responses in *α*6*β*2* nAChR function in the accumbens of human smokers and whether phasic dopamine release is increased or decreased by chronic nicotine exposure in smokers.

The fact that nAChRs located on dopamine terminals exert such a robust inhibitory influence on activity-dependent dopamine release suggests that endogenous acetylcholine derived from striatal cholinergic neurons controls the degree to which dopamine transmission is increased in the striatum when these cells fire in reward-relevant burst patterns of activity (Zhou et al., 2001; Brimblecombe et al., 2018). Indeed, optical stimulation of cholinergic interneurons in the striatum was shown to increase dopamine release in an impulse-independent manner through a direct action of acetylcholine at *β*2* nAChRs located on the terminals of dopamine neurons (Cachope et al., 2012; Threlfell et al., 2012). Hence, nAChR-mediated cholinergic transmission in the striatum is likely to facilitate dopamine release when midbrain dopamine neurons engage in tonic firing patterns but inhibit dopamine release when these cells engage in burst firing patterns (Cachope et al., 2012; Threlfell et al., 2012). By desensitizing nAChRs in the striatum, nicotine may facilitate reward-related burst firing in the accumbens to enhance brain reward function. Conversely, stimulation of nAChRs in accumbens may enhance dopamine release even when dopamine neurons are tonically firing, which may also contribute to reward-related behaviors (Cover et al., 2019). Precisely how these different sources of nAChR-related dopamine release interact and their relevance to discrete behavioral responses to nicotine are currently unclear.

## III. Dopamine Mechanisms of Nicotine Aversion

In addition to its rewarding effects, nicotine also has aversive effects that motivate avoidance behaviors in humans, nonhuman primates, rats, and mice (Shoaib et al., 1997; Spealman, 1983; Sartor et al., 2010; Fowler et al., 2011). The same doses of nicotine that laboratory animals will work to obtain in self-administration experiments also have punishing properties that animals will work to avoid (Spealman, 1983). Whether nicotine is rewarding or aversive depends on whether the drug is consumed volitionally or delivered nonvolitionally, respectively (Spealman and Goldberg, 1978; Goldberg et al., 1981; Goldberg and Spealman, 1982; Spealman, 1983). Individuals who are less sensitive to the aversive effects of nicotine are more likely to be heavy smokers (Jensen et al., 2015). Similarly, those who suffer from psychiatric disorders associated with heavy smoking are often less sensitive to nicotine aversion (Williams et al., 2013), which likely contributes to their high levels of tobacco smoking. Sensitivity to nicotine aversion is thought to influence the likelihood of transitioning from occasional to regular tobacco use (Sartor et al., 2010; Fowler and Kenny, 2014). Interestingly, menthol and other additives contained in cigarettes and non-nicotine components of tobacco smoke can attenuate the aversive effects of nicotine (D’Silva et al., 2018; Harris et al., 2019). Hence, a better understanding of the neurobiological mechanisms of nicotine aversion may reveal new insights into genetic and environmental factors that influence vulnerability to tobacco dependence and the brain systems that undergo nicotine-induced adaptions to establish and maintain the tobacco smoking habit. As described above, the stimulatory effects of nicotine on midbrain dopamine neurons contribute to the rewarding properties of the drug that motivate tobacco smoking. However, dopamine transmission also regulates avoidance behaviors (Acquas et al., 1989; Shippenberg et al., 1993; Bromberg-Martin et al., 2010). This raises the possibility that midbrain dopamine neurons contribute to the aversive actions of nicotine. Consistent with this possibility, allelic variation in the genes that encode the dopamine D4 and D2 receptors has been shown to influence aversion-related responses to nicotine delivered by nasal spray in humans (Perkins et al., 2008). Lesioning the pedunculopontine tegmental nucleus (PPTg), which provides a major cholinergic projection to the VTA (Good and Lupica, 2009), blocked the rewarding effects of nicotine injected directly into the VTA and enhanced its aversive effects (Laviolette et al., 2002) (see Fig. 2). Infusion of a *β*2* nAChR antagonist or a mixed *α*7/*α*6* nAChR antagonist into the VTA blocked the aversive effects of nicotine in a place conditioning procedure (Laviolette and van der Kooy, 2003b). These findings suggest that endogenous cholinergic transmission in the VTA derived from PPTg inputs and acting through locally expressed *β*2* nAChRs regulates the rewarding and aversive properties of nicotine. Infusion of an NMDA glutamate receptor antagonist into the VTA similarly blocked the aversive effects of nicotine in a place conditioning procedure (Laviolette and van der Kooy, 2003b). Antagonism of NMDA receptors also rendered previously rewarding doses of nicotine aversive in rats, as measured using an ICSS procedure (Kenny et al., 2009). Nicotine-enhanced NMDA receptor-mediated glutamatergic transmission in the VTA contributes to the stimulatory effects of nicotine on accumbal dopamine release, with the role for NMDA receptor-mediated transmission in nicotine-enhanced dopamine release particularly important at higher doses of nicotine known to have aversive behavioral effects (Schilstrom et al., 1998; Fu et al., 2000). Hence, glutamatergic transmission in the VTA regulates components of dopamine transmission involved in both the rewarding and aversive effects of nicotine.

Focal lesion of dopamine inputs to the NAc shell accomplished by local infusion of the toxin 6-hydroxydopamine decreased the CPP response to intravenous nicotine injections in rats (Sellings et al., 2008). Conversely, lesion of dopamine inputs to the NAc core enhanced nicotine-induced CPP in rats (Sellings et al., 2008). NAc core dopamine lesions also attenuated conditioned taste avoidance triggered by intravenous nicotine infusions (Sellings et al., 2008). Systemic or intra-NAc injections of the mixed dopamine D1 and D2 receptor antagonist *α*-flupenthixol blocked only the aversive effects of nicotine in a place conditioning procedure (Laviolette and van der Kooy, 2003a). Similarly, blockade of D2 dopamine receptors in the shell region of the NAc or D1 dopamine receptors in the NAc core abolished the aversive properties of nicotine injected directly into the VTA (Laviolette et al., 2008; Grieder et al., 2012). These findings provide compelling evidence that the rewarding and aversive effects of nicotine are encoded by VTA-derived dopamine transmission in the accumbens, with the NAc core playing a prominent role in nicotine aversion. Notably, the aversive effects of nicotine were abolished in *α*5 nAChR subunit knockout mice in a manner that phenocopies the effects of dopamine receptor antagonists (Grieder et al., 2017). This suggests that the component of nicotine-enhanced dopamine transmission in the striatum mediated by (*α*4*β*2)_2_*α*5 nAChRs may signal the aversive but not the rewarding effects of nicotine.

## IV. Cellular Mechanisms in VTA of Nicotine Reward and Aversion

### A. Balance between nAChR Signaling in VTA Dopaminergic and GABAergic Neurons

Recent findings have shed important light on the cellular mechanisms in the VTA that regulate nicotine aversion. Using mice with floxed alleles of the *Chrna4* gene, it was shown that Cre recombinase-mediated conditional deletion of *α*4 nAChR subunits in the ventral midbrain increased nicotine self-administration (oral intake) only when high-concentration nicotine solutions were available (Peng et al., 2017). This pattern of nicotine self-administration behavior is thought to occur when the punishing properties of higher nicotine doses are attenuated, which disinhibits self-administration of aversive doses that would otherwise suppress intake (Fowler et al., 2011). *α*4 nAChR subunits were knocked down in both dopamine and GABAergic neurons in the VTA of mice in this study (Peng et al., 2017), raising the possibility that at least some fraction of those VTA dopamine neurons that express *α*4* nAChR regulate the aversive reactions to nicotine. As noted above, virus-mediated re-expression of the *Chrnb2* gene in VTA GABAergic neurons rescued nicotine-induced CPP in *β*2 subunit knockout mice (Grieder et al., 2019). This study also reported that virus-mediated re-expression of *Chrnb2* only in VTA dopamine neurons failed to rescue nicotine reward in *β*2 subunit knockout mice but instead rendered the knockout mice sensitive to nicotine aversion (Grieder et al., 2019). Virus-mediated re-expression of *Chrnb2* simultaneously in VTA dopamine and GABA neurons but not in either cell type alone was necessary to “rescue” nicotine-induced burst firing of VTA dopamine neurons and reconstitute sensitivity to the reinforcing properties of nicotine in *β*2 subunit knockout mice, as measured using an intra-VTA self-administration procedure (Tolu et al., 2013). However, when *Chrnb2* was re-expressed only in VTA GABA neurons in *β*2 subunit knockout mice, these cells were persistently activated by nicotine, and only aversive behavioral responses to nicotine were detected (Tolu et al., 2013). This is consistent with the observation that VTA GABA neurons are more robustly activated by aversive than rewarding doses of nicotine (Dehkordi et al., 2018). These findings support a complex regulatory mechanism whereby concerted actions by nicotine on *β*2* nAChRs expressed by both VTA dopamine and GABA neurons are required to trigger reward-relevant burst firing of dopamine neurons, with this action necessary to experience the reinforcing properties of nicotine that support self-administration behavior. By contrast, nicotine acting on VTA dopamine or GABA neurons alone can promote aversion (Tolu et al., 2013; Grieder et al., 2019), likely by blocking the ability of dopamine neurons to engage in burst firing.

### B. Anterior-Posterior Domains of the VTA

The fact that VTA dopamine neurons regulate both the rewarding and aversive properties of nicotine reflects the remarkable functional heterogeneity of these cells, with discrete populations likely specializing in positive or negative reinforcement processes (Lammel et al., 2011, 2012, 2014). Dopamine neurons located in the posterior VTA (pVTA) but not the anterior VTA (aVTA) regulate the rewarding properties of nicotine that support self-administration behavior (Ikemoto et al., 2006) (Fig. 3). Similarly, ethanol, cocaine, opioids, and cholinergic agonists are all volitionally self-administered into the pVTA but are not reliably self-administered into the aVTA (Rodd-Henricks et al., 2000; Ikemoto and Wise, 2002; Zangen et al., 2002; David et al., 2004; Rodd et al., 2005). The pVTA but not aVTA also regulates the locomotor-stimulating properties of these drugs (Ikemoto et al., 2003; Sanchez-Catalan et al., 2009). aVTA and pVTA dopamine neurons are distinguished by distinct morphologic features (Zhao-Shea et al., 2011) and project to different regions of the striatum (Ikemoto et al., 2006). Specifically, pVTA dopamine neurons project predominantly to medial NAc shell and medial olfactory tubercle, whereas aVTA neurons instead project to the accumbens core, lateral tubercle, and dorsal striatum (Ikemoto et al., 2006). Dopamine neurons in the pVTA show greater responsiveness to nicotine than those in the aVTA, as measured by nicotine-induced cFos immunoreactivity (Zhao-Shea et al., 2011), consistent with the fact that pVTA contains denser concentrations of nAChRs than aVTA (Zhao-Shea et al., 2011). Moreover, pVTA dopamine neurons are activated by rewarding doses of nicotine, whereas aVTA dopamine neurons are activated only by high concentrations of nicotine that have aversive properties (Fonck et al., 2005; ZhaoShea et al., 2011). Within the pVTA, *α*4* and *α*6* nAChR subtypes are thought to regulate the rewarding and reinforcing properties of nicotine (ZhaoShea et al., 2011; Exley et al., 2012; Liu et al., 2012; Engle et al., 2013).

**Fig. 4:**
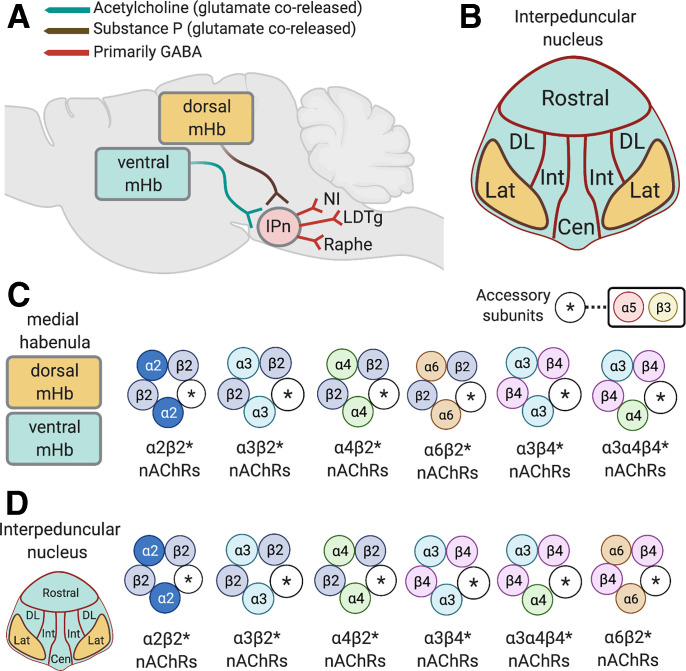
Organization and nAChR subtypes of the habenula-interpeduncular nucleus circuit. (A) Substance P–expressing neurons in in the dorsal region of the mHb and cholinergic neurons in the ventral mHb corelease glutamate and project to the IPn. The IPn send predominately GABAergic projections to the raphe nuclei, LDTg, and the nucleus incertus (NI). (B) Graphical representation of subregions of the interpeduncular nucleus, including the rostral, dorsolateral (DL), intermediate (I), and central (Cen) nuclei that receive input from cholinergic neurons in the ventral mHb. (C) Major stoichiometries of nAChRs predicted to be expressed presynaptically and postsynaptically in the mHb. (D) Major stoichiometries of nAChRs predicted to be expressed presynaptically and postsynaptically in the mHb in the IPn.

### C. Medial-Lateral Domains of the VTA

In addition to antero-posterior heterogeneity, VTA neurons also demonstrate a medial-lateral functional gradient (Lammel et al., 2011, 2012, 2014) (Fig. 3). Indeed, neurons in the medial VTA (VTA^MED^) are more likely to be GABAergic or glutamatergic than neurons in the lateral VTA (VTA^LAT^), where the majority of the dopamine neurons are concentrated (Lammel et al., 2008; Hnasko et al., 2012; Root et al., 2014a,b; Ntamati and Luscher, 2016; Yan et al., 2018, 2019). Projections from the PPTg to the VTA are known to regulate the rewarding properties of nicotine (Corrigall et al., 2002; Alderson et al., 2006, 2008; Maskos, 2008). These PPTg inputs synapse preferentially onto neurons in lateral domains of the VTA (PPTg-VTA^LAT^ neurons), which in turn project to the NAc medial shell (Lammel et al., 2012). Optical stimulation of PPTg-VTA^LAT^ neurons can elicit reward-related behaviors that are blocked by dopamine receptor antagonists infused into the NAc shell (Lammel et al., 2012). Rewarding doses of nicotine are known to preferentially increase dopamine transmission in the NAc shell (Nisell et al., 1997; Lecca et al., 2006), which may reflect the recruitment of this PPTg-VTA-NAc shell reward circuit (Fig. 3). In addition to reward-relevant VTA^LAT^ dopamine neurons, cholinergic inputs from PPTg and the adjacent laterodorsal tegmental nucleus (LDTg) also project to VTA^MED^ glutamatergic neurons that have been heavily implicated in aversion-related behavioral states (Root et al., 2014a; Lammel et al., 2015; Qi et al., 2016; McGovern et al., 2020) and are known to express functional *α*4* and *α*6* nAChRs (Yan et al., 2018, 2019). Recent data have revealed that PPTg- and LDTg-derived cholinergic transmission in the VTA bidirectionally modulates reward and aversion behaviors (Dautan et al., 2016). This suggests that the rewarding and aversive effects of nicotine may reflect the nonphysiologic recruitment of these processes, which are normally controlled by endogenous cholinergic transmission acting at local nAChRs in the VTA (Fig. 3).

In contrast to PPTg inputs to the VTA, which predominately but nonexclusively target VTA^LAT^ neurons, inputs from the lateral habenula synapse preferentially onto neurons in the VTA^MED^ [lateral habenula (LHb)-VTA^MED^ neurons] (Fig. 3). VTA^MED^ neurons targeted by the LHb in turn project to the medial prefrontal cortex (mPFC) (Lammel et al., 2012) (Fig. 3). Optical stimulation of LHb-VTA^MED^ neurons elicits aversion-related behaviors that are blocked by dopamine receptor antagonists infused in the mPFC (Lammel et al., 2012). Nicotine is known to increase dopamine release in the mPFC (Rossi et al., 2005), which could reflect the engagement of this LHb-VTA-mPFC aversion circuit. VTA^MED^ dopamine neurons send a reciprocal projection back to the LHb, with these VTA^MED^ dopamine neurons known to corelease both glutamate and GABA (Root et al., 2014b) (Fig. 3). VTA-derived glutamate transmission in the LHb regulates aversion-related behaviors (Root et al., 2014a), whereas VTA-derived GABA transmission in the LHb regulates reward-related behaviors (Stamatakis et al., 2013). Notably, nicotine increases both glutamatergic and GABAergic transmission in the LHb, likely by stimulating *α*4*β*2* and *α*6* nAChRs expressed by VTA dopamine inputs to the habenula (Zuo et al., 2016). This suggests that nicotine bidirectionally modulates the activity of aversion-relevant neurons in the LHb by altering the balance of VTA-derived glutamatergic and GABAergic transmission. In addition to their projections to aversion-related neurons in VTA^MED^, LHb neurons also send a prominent projection to GABA neurons in the rostromedial tegmental nucleus (RMTg) (Jhou et al., 2009b), also known as the “tail” of the VTA. In turn, RMTg GABA neurons project to the VTA where they can inhibit dopamine neurons to elicit aversion-related behaviors (Jhou et al., 2009a,b; Hong et al., 2011; Barrot et al., 2012). Nicotine markedly enhances the activity of RMTg neurons (Lecca et al., 2011), providing another mechanism by which nicotine can modulate the activity of reward-related dopamine neurons in the VTA. Hence, nAChR-induced modulation of synaptic inputs to VTA^LAT^ and VTA^MED^ neurons and modulation of the projections from VTA^LAT^ and VTA^MED^ neurons to downstream brain sites likely contribute to the reinforcing actions of nicotine (Fig. 3).

Recent studies have begun to reveal the complex interplay between dopamine neurons located in medial and lateral domains of the VTA and how nicotine modifies these interactions (Fig. 3). Specifically, VTA^MED^ neurons were shown to provide both excitatory and inhibitory input to VTA^LAT^ dopamine neurons (Yan et al., 2019). Interestingly, a discrete subpopulation of the VTA^MED^ neurons expresses *β*2* nAChRs and coreleases both glutamate and GABA onto VTA^LAT^ dopamine neurons (Yan et al., 2019). Nicotine had bidirectional effects on VTA^MED^-derived excitatory glutamatergic transmission in VTA^LAT^ dopamine neurons, increasing excitatory input to approximately half of the recorded neurons in VTA^LAT^ and decreasing excitatory input to the other half (Yan et al., 2019). By contrast, nicotine uniformly decreased VTA^MED^-derived inhibitory transmission in VTA^LAT^ dopamine neurons (Yan et al., 2019). These findings suggest that nicotine-induced inhibition of VTA^MED^-derived GABAergic input and simultaneous stimulation of VTA^MED^-derived glutamatergic input to VTA^LAT^ “reward” dopamine neurons contribute to the rewarding properties of the drug, whereas nicotine-induced inhibition of VTA^MED^-derived glutamatergic input to VTA^LAT^ reward neurons contributes to the aversive properties of the drug. More broadly, these findings suggest that complex modulation of excitatory and inhibitory transmission onto VTA^MED^ and VTA^LAT^ dopamine neurons by nicotine acting through local neurons within the VTA and on long-range inputs to the VTA determines whether nicotine has rewarding or aversive effects. Further investigation will be required to better define the complex effects of nicotine on local and long-range synaptic inputs to VTA^MED^ and VTA^LAT^ dopamine neurons and the consequences of these actions on downstream brain sites.

The findings described above suggest that VTA-derived dopamine, glutamate, and GABA transmission in the accumbens, LHb, mPFC, and elsewhere in the brain likely contribute to aversion-related responses to nicotine. However, dopamine receptor antagonists block nicotine aversion only in animals with a limited history of nicotine exposure (Tan et al., 2009). By contrast, the same dopamine manipulations block the rewarding effects of nicotine in animals that have previously been exposed to the drug (Tan et al., 2009). This apparent switch in the role for dopamine transmission that occurs with repeated exposure to nicotine from initially regulating nicotine aversion to instead regulating nicotine reward coincides with an increase in the incidence of nicotine-induced burst firing of VTA dopamine neurons and a decrease in the baseline activity of VTA GABAergic neurons (Tan et al., 2009). Hence, VTA dopamine neurons may participate in aversive reactions to nicotine that influence the likelihood of transitioning from initial tobacco use to regular intake (de Wit & Phillips 2012). Furthermore, adaptive responses in dopamine-mediated aversion systems driven in part by alterations in the function of VTA GABA neurons may contribute to this transition process. However, these findings also suggest that dopamine transmission is unlikely to participate in aversive responses to nicotine once regular nicotine use has been established and that other aversion-related brain systems influence patterns and amounts of tobacco smoking in smokers (Fowler and Kenny, 2014). This raises the important issue of the identity of dopamine-independent brain systems that regulate nicotine aversion in tobacco smokers and nicotine-experienced laboratory animals, which is considered in more detail below.

## V. Nondopamine Mechanisms of Nicotine Aversion

### A. Human Genetics Reveal nAChR Subtypes that Regulate Nicotine Intake

As noted above, nicotine has rewarding properties that motivate intake and aversive properties that motivate avoidance. These competing positive and negative effects likely explain the inverted U-shape of the dose-response curve for self-administered nicotine seen in humans, nonhuman primates, and laboratory rodents responding under fixed-ratio schedules. Lower doses elicit primarily rewarding effects that motivate self-administration, whereas higher doses elicit mixed rewarding/aversive effects that necessitate careful titration of intake (Goldberg and Spealman, 1982; Risner and Goldberg, 1983; Corrigall and Coen, 1989; Harvey et al., 2004; DeNoble and Mele, 2006; Fowler and Kenny, 2011). One potential explanation for these opposing motivational properties of nicotine and the shape of the dose-response curve is that the same population of nAChRs regulates both the rewarding and aversive effects of nicotine, with lower doses activating these nAChRs and higher doses desensitizing and thereby inactivating these nAChRs (Pidoplichko et al., 1997). An alternative explanation is that lower unit doses of nicotine engage high-affinity nAChRs located in brain reward circuits to motivate nicotine self-administration, whereas higher nicotine doses engage low-affinity nAChRs in brain aversion circuits that motivate nicotine avoidance. According to this framework, nicotine intake is titrated to maximize the activation of reward-related nAChRs while minimizing the activation of aversion-related nAChRs. The mesoaccumbens dopamine system is enriched in (*α*4*β*2)(*α*6*β*2)*β*3 nAChRs (Grady et al., 2007), which have the highest sensitivity to nicotine of any native nAChR so far identified (Grady et al., 2007) and are thought to contribute to nicotine reward. Until recently, little was known about the identity of the putative “low-affinity” nAChRs activated by higher nicotine doses or the contributions of the brain regions in which they are located to the control of nicotine intake.

The *CHRNA5-CHRNA3-CHRNB4* gene cluster located in chromosome region 15q25 encodes the *α*5, *α*3, and *β*4 nAChR subunits, respectively. nAChRs that incorporate subunits encoded by this gene cluster are known as “low-affinity” nAChRs because they bind nicotine far less efficiently than the so-called high affinity *β*2* nAChRs (Zoli et al., 1998). They are also known as the ganglionic nAChRs because of their dense expression in neurons of the autonomic nervous system (Kemp and Morley, 1986). Large-scale human genetics studies have shown that allelic variation across the entire *CHRNA5-CHRNA3-CHRNB4* gene cluster is associated with increased vulnerability to tobacco dependence and higher numbers of cigarettes smoked per day (Amos et al., 2008; Ware et al., 2011; Marques-Vidal et al., 2011; Gallego et al., 2013; Liu et al., 2018; Perez-Morales et al., 2018). In particular, individuals who carry risk alleles in *CHRNA5* are less sensitive to the aversive effects of nicotine (Jensen et al., 2015) and more likely to be heavy smokers (Saccone et al., 2007; Berrettini et al., 2008; Lips et al., 2009; Ohi et al., 2019). A particular variant in C*HRNA5* (rs16969968) that gives rise to an amino-acid substitution (*α*5-398D→*α*5-398N) thought to decrease the function of *α*5* nAChRs that incorporate the mutant subunit increases tobacco dependence risk by ∼30% in individuals who carry a single copy of the gene variant and doubles the risk in individuals who carry two copies of the variant (Bierut et al., 2008; Wang et al., 2009). Alleles in *CHRNA5* are also associated with heavy smoking (Berrettini et al., 2008; Bierut et al., 2008; Grucza et al., 2008; Stevens et al., 2008), early onset of smoking behavior (Weiss et al., 2008), and with “pleasurable buzz” from tobacco (Sherva et al., 2008). Hence, low-affinity *α*3*β*4* nAChRs and *α*5* nAChR subtypes are of considerable interest in the context of nicotine aversion.

### B. α5*, α3*, and β4* nAChRs Regulate Nicotine Avoidance

As noted above, the so-called “ganglionic” *α*3*β*4* nAChRs are expressed in the CNS, where they account for low-affinity nicotine binding sites (Flores et al., 1992). They are far less abundant than *α*4*β*2* nAChRs and are concentrated in just a few brain regions, most notably the medial habenula and interpeduncular nucleus (Clarke et al., 1985; Cimino et al., 1992). In heterologous expression systems, *α*5 subunits can incorporate into the *α*3*β*4* nAChRs, which modifies their sensitivity to agonist-induced desensitization (Wang et al., 1996; Mao et al., 2008; Gotti et al., 2007; Perry et al., 2007; Kuryatov et al., 2008). However, *α*5 subunits incorporate more efficiently into *α*4*β*2 nAChRs (Gotti et al., 2007; Perry et al., 2007; Kuryatov et al., 2008; Mao et al., 2008). Similar to *α*5, *β*3 can also functions as an accessory subunit in *α*3*β*4* and *α*3*β*2* nAChRs (Gotti et al., 2009). *β*3 has a dominant-negative function when incorporated into *α*3*β*4* nAChRs, rendering the resulting receptor complex nonfunctional (Palma et al., 1999; Broadbent et al., 2006). Less frequently, *α*3 nAChRs can coassemble with *β*2 subunits to form a functional *α*3*β*2* nAChR subtype.

*α*5 nAChR subunit knockout mice and their wild-type littermates intravenously self-administer nicotine according to an inverted U-shaped dose-response curve (Fowler et al., 2011), as expected based on previous findings in humans and laboratory animals (Goldberg et al., 1981; Henningfield and Goldberg, 1983; Corrigall and Coen, 1989; Le Foll et al., 2007). Levels of nicotine self-administration were similar in *α*5 subunit knockout and wild-type mice on the “ascending” portion of the dose-response curve when lower unit doses of nicotine were available (Fowler et al., 2011). By contrast, responding was much higher in the knockout mice than their wild-type littermates when higher unit doses of nicotine were on the “descending” portion of the dose-response curve (Fowler et al., 2011). Transgenic rats that express a mutant form of the *α*5 nAChR subunit gene modified to contain the same amino-acid substitution caused by the rs16969968 risk allele also self-administered greater quantities of nicotine than wild-type rats but only when higher unit doses of nicotine on the descending portion of the dose-response curve were available (Forget et al., 2018). Enhanced responding for nicotine as the unit dose increases is thought to reflect an intensification of the reinforcing properties of the drug, thereby motivating higher levels of intake (Lynch and Carroll, 2001). Diminished responding for nicotine as the dose increases reflects greater restraint over intake to avoid the increasingly aversive effects of higher drug doses (Henningfield and Goldberg, 1983; Lynch and Carroll, 2001), more rapid development of drug satiation (Lynch and Carroll, 1999, 2001), or the manifestation of performance-disrupting actions of the drug (Lynch and Carroll, 1999, 2001). Hence, deletion of *α*5 nAChR subunits has dissociable effects on the motivational processes that control nicotine intake. The stimulatory effects of nicotine on brain reward systems that motivate nicotine use were unaltered by *α*5 subunit knockout, whereas the aversive effects of nicotine that limit its self-administration were attenuated by *α*5* nAChR deficiency. Consistent with this interpretation, *α*5 nAChR subunit knockout mice had similar sensitivity to the rewarding effects of nicotine as wild-type mice but markedly reduced sensitivity to the aversive effects of nicotine, as measured using CPP and ICSS procedures (Jackson et al., 2010; Fowler et al., 2011, 2013; Grieder et al., 2017). More recently, *Chrna3^t^*^m1.1Hwrt^ hypomorphic mice, which express much lower levels of *α*3 nAChR subunits than wild-type mice (Caffery et al., 2009), were shown to self-administer greater numbers of intravenous nicotine infusions than their wild-type littermates, with this effect most apparent when nicotine doses on the descending portion of the dose-response curve were available (Elayouby et al., 2021). Similarly, *β*4 nAChR subunit knockout mice also self-administered greater numbers of nicotine infusions directly into the VTA when higher nicotine doses were available (Husson et al., 2020), although these animals self-administered less nicotine across all doses tested when the drug was delivered by intravenous infusion (Harrington et al., 2016). Transgenic mice that overexpressed *β*4 nAChR subunits in only those neurons that constitutively express *β*4 subunits showed enhanced nicotine aversion and decreased oral nicotine intake (Frahm et al., 2011). Similarly, lentivirus-mediated expression in the brains of mice of mutant gain-of-function *β*4 nAChR subunits, which incorporated *CHRNB4* variants associated with reduced risk of tobacco dependence, increased aversion to nicotine (Slimak et al., 2014). Together, these findings suggest that *α*5*, *α*3*, and *β*4* nAChRs regulate nicotine aversion.

### C. Low-Affinity nAChRs Are Enriched in Medial Habenula and Interpeduncular Nucleus

*α*3 and *β*4 nAChR subunit transcripts are expressed at low levels in the midbrain dopamine system (Klink et al., 2001; Azam et al., 2002), and functional *α*3* and *β*4* nAChRs have not been reliably detected in the VTA. *α*5 subunit transcripts are expressed at modest levels in the VTA, although functional (*α*4*β*2)_2_*α*5 nAChRs are thought to be expressed by dopamine and nondopamine neurons in the VTA (Fig. 2). By contrast, high densities of *α*5, *α*3 and *α*4 nAChR subunit transcripts are detected in the mHb, interpeduncular nucleus (IPn), and nucleus of the solitary tract (nTS) (Marks et al., 1992; Winzer-Serhan and Leslie, 1997; Sheffield et al., 2000; De Biasi and Salas, 2008; O'Leary et al., 2008; Grady et al., 2009; Frahm et al., 2011; Tuesta et al., 2011, 2017; Hsu et al., 2013; Antolin-Fontes et al., 2015; Morton et al., 2018; Husson et al., 2020). The mHb is located immediately adjacent to the LHb in the epithalamus, projects almost exclusively to the IPn, and is subdivided into four functional domains: superior, inferior, central, and lateral nuclei (Aizawa et al., 2012). Neurons in the inferior, central, and lateral domains produce acetylcholine, whereas those in the superior portion produce substance P (neurokinin 1) or the cytokine interleukin-18 (Contestabile et al., 1987; Andres et al., 1999; Aizawa et al., 2012; Kobayashi et al., 2013; Quina et al., 2017). All mHb neurons are thought to produce and corelease glutamate, which is considered the major functional transmitter at the habenula-IPn synapse (Ren et al., 2011; Aizawa et al., 2012; Aizawa and Zhu, 2019). The cholinergic neurons in ventral portions of the mHb project to central, rostral, and intermediate domains of the IPn (Kimura et al., 1981; Houser et al., 1983; Qin and Luo, 2009) where they assume highly unusual “serpentine” patterns of innervation (Ramón y Cajal, 1953; Lenn, 1976; Herkenham and Nauta, 1979; Lenn et al., 1983), whereas substance P–positive neurons in dorsal MHb neurons project to the lateral domains of the IPn (Qin and Luo, 2009). nAChRs located on the presynaptic terminals of mHb inputs to the IPn regulate neurotransmitter release into the IPn, and the mHb-IPn synapse has long served as a model system for nAChR signaling in the brain (Sastry, 1978; Brown et al., 1983; McGehee et al., 1995). In addition, postsynaptic nAChRs located on IPn neurons can generate retrograde signaling molecules, including endocannabinoids and nitric oxide, that provide a source of feedback inhibition onto mHb inputs (Rodrigo et al., 1994; Ables et al., 2017; Quina et al., 2017; Melani et al., 2019). Little is currently known about the function of the mHb-IPn circuit, although available data suggest that mHb-derived cholinergic, glutamatergic, and substance P transmission in the IPn modulated by GABA_B_, cannabinoid 1 (CB1), or glycine receptors regulates the encoding of memories related to aversive stimuli or events (Thompson, 1960; Donovick et al., 1970; Agetsuma et al., 2010; Yamaguchi et al., 2013; Soria-Gomez et al., 2015; Zhang et al., 2016; Koppensteiner et al., 2017; Geng et al., 2019; Melani et al., 2019). The mHb-IPn circuit may also coordinate adaptive physiologic and behavioral responses to stressful or anxiety-provoking stimuli (Lisoprawski et al., 1981; Murray et al., 1994; Shumake et al., 2003; Hsu et al., 2012, 2016; Xu et al., 2018; Duncan et al., 2019; Sherafat et al., 2020).

### D. nAChR Signaling in the mHb-IPn Circuit Regulates Nicotine Avoidance

Considering the high densities of *α*5, *α*3 and *α*4 nAChR subunits in the habenula-IPn circuit (Marks et al., 1992; De Biasi and Salas, 2008) and the role for this circuit in regulating responses to aversive stimuli, it is perhaps not surprising that evidence has accumulated over the past decade implicating the mHb-IPn circuit in coordinating aversive behavioral responses to nicotine. Infusion of the local anesthetic lidocaine into the mHb or IPn of rats increased their nicotine intake via intravenous infusions (Fowler et al., 2011), suggesting that the mHb-IPn circuit exerts an inhibitory influence over nicotine-taking behavior. IPn neurons that receive excitatory inputs from the mHb are perhaps the most nicotine-responsive cells in the brain, as measured by nicotine-induced cFos immunoreactivity (Ren and Sagar, 1992; Pang et al., 1993; Salminen et al., 2000). Nicotine-induced cFos expression in IPn was greatly reduced in *α*5 subunit knockout mice compared with wild-type mice (Fowler et al., 2011). nAChR function was also decreased in the mHb and IPn of *α*5 subunit knockout mice, as measured by the rubidium efflux assay (Fowler et al., 2011). By contrast, nicotine-induced activation of the VTA was similar in wild-type and *α*5 knockout mice (Fowler et al., 2011). This suggests that *α*5* nAChRs are an important functional subtype that regulates the stimulatory effects of nicotine on the mHb-IPn circuit. The elevated level of nicotine self-administration in *α*5 subunit knockout mice was normalized by virus-mediated re-expression of *α*5 subunits specifically in the mHb-IPN circuit (Fowler et al., 2011). Conversely, lentivirus-mediated expression of short interfering RNAs (shRNAs) to knock down *α*5 or *α*3 nAChR subunits in the mHb or IPn increased nicotine self-administration in rats (Fowler et al., 2011; Elayouby et al., 2021), with these effects most apparent when higher unit doses were available. Conversely, lentivirus-mediated expression of mutant gain-of-function *β*4 nAChR subunits in the mHb of mice increased nicotine aversion and decreased their nicotine intake (Slimak et al., 2014). Infusion of the *α*3*β*4* nAChR antagonists 18-methoxycoronaridine (18-MC) or *α*-conotoxin AuIB into the mHb or IPn but not the *α*3*β*2* nAChR antagonist *α*-conotoxin MII increased nicotine intake in rats (Fowler et al., 2011; Elayouby et al., 2021). By contrast, infusion of 18-MC into the VTA had no effect on nicotine self-administration in rats (Glick et al., 2011). These findings suggest that stimulatory effects of nicotine on the mHb-IPn circuit mediated by *α*5* and *α*3*β*4* nAChRs decrease the motivation to consume nicotine.

### E. nAChR Signaling in mHb-IPn Circuit Regulates the Reward-Inhibiting Effects of Nicotine

Using an ICSS procedure, it was shown that shRNA-mediated knockdown of *α*5 nAChR transcripts in the mHb-IPn circuit of rats had no effects on the threshold-lowering effects of lower nicotine doses but attenuated the threshold-elevating effects of higher nicotine doses in rats (Fowler et al., 2011). These findings in rats recapitulate the same pattern of effects described above in *α*5 nAChR subunit knockout mice (Fowler et al., 2013). The ICSS threshold-lowering doses of nicotine are thought to reflect nicotine-induced enhancement of brain reward activity (Kenny and Markou, 2006). Conversely, the ICSS threshold-elevating effects of nicotine are through to reflect the reward-inhibiting effects of the drug that contribute to nicotine aversion and motivate nicotine avoidance (Schaefer and Michael, 1986; Fowler et al., 2011). Rats regulate their intravenous nicotine self-administration to achieve maximal lowering of ICSS thresholds (Kenny and Markou, 2006), suggesting that nicotine intake is titrated to obtain this action of nicotine while avoiding the threshold-elevating effects of the drug. Hence, disruption of *α*5* nAChR-mediated signaling in the mHb-IPn circuit likely increases nicotine intake by attenuating the reward-inhibiting effects of nicotine and extending the range of nicotine doses that are reward-enhancing (Fowler et al., 2011). This is consistent with the observation that aversive responses to nicotine are attenuated in individuals who carry the rs16969968 polymorphism in C*HRNA5* (Jensen et al., 2015). In contrast to dopamine-mediated mechanisms of nicotine aversion, which were detected only when animals had limited previous exposure to nicotine (Tan et al., 2009) (see above), the aversion mediated by the mHb-IPn circuit was observed even in animals that had experienced extended periods of daily access to high unit doses of nicotine (Fowler et al., 2011; Elayouby et al., 2021). This suggests that nAChR transmission in the mHb-IPn circuit is likely to control nicotine intake in human smokers even after the habit has been established. Indeed, functional brain imaging studies have shown that nicotine modulates habenular activity in both nonsmokers and habitual smokers (Flannery et al., 2019; Jennings et al., 2020). Notably, mHb neurons receive excitatory and inhibitory synaptic input almost exclusively from the posterior septum via the stria medullaris (Sperlagh et al., 1998; Qin and Luo, 2009; Yamaguchi et al., 2013; Otsu et al., 2018), with ATP thought to function as a neurotransmitter at the septo-mHb synapse (Edwards et al., 1992; Sperlagh et al., 1995). Nevertheless, little is known about function of these septal inputs to the mHb, and their potential involvement in behavioral responses to nicotine has not been explored.

### F. Stoichiometries of nAChRs Expressed in the mHb-IPn Circuit

The mHb contains some of the highest concentrations of nAChRs in the brain, particularly those that account for the “low-affinity” nicotine binding sites (i.e., do not contain *β*2 subunits) (Clarke et al., 1985; Wada et al., 1989; Marks et al., 1992; Zoli et al., 1998). As noted above, the highest concentrations of *α*5, *α*3, and *α*4 nAChR subunits in the brain are detected in the mHb and IPn. nAChRs expressed by mHb neurons are highly calcium-permeable (Mulle et al., 1992; Guo and Lester, 2007) and are expressed along the entire length of mHb neurons, including their soma, axons, and terminals in the IPn (Mulle et al., 1991; Lena et al., 1993; Passlick et al., 2018). Electrophysiological recordings of mHb neurons revealed two discrete nAChR-mediated currents, suggesting that at least two populations of nAChRs are expressed in the mHb (Mulle et al., 1991; Connolly et al., 1995). Both nAChR populations are desensitized by nicotine (Lester and Dani, 1995; Hicks et al., 2000; Duncan et al., 2019). Neurons in the ventrolateral portion of the mHb are most responsive to nicotine (Fonck et al., 2009; Shih et al., 2014), with *α*3 and *β*4 nAChR subunits expressed by most neurons in this region (Shih et al., 2014). Nicotine-responsive neurons in ventrolateral mHb provide synaptic inputs to rostral and dorsomedial domains of the IPn (Mulle et al., 1991; Shih et al., 2014). Blockade of *α*3*β*4* nAChRs has been shown to greatly reduce but not eliminate the stimulatory effects of nicotine on mHb neurons (Quick et al., 1999; Elayouby et al., 2021). Immunoprecipitation studies support these findings and suggest that relatively uncommon nAChR subtypes are expressed by mHb neurons (Fig. 4). Specifically, mHb neurons express high concentrations of *α*3*β*4* nAChRs but also *α*3*β*2*, *α*3*α*4*β*4*, and *α*4*β*2*; nAChRs (Grady et al., 2009); and much smaller populations of *α*2*β*2* and *α*6*β*2* nAChRs (Fig. 4). A large proportion of these nAChRs contained *α*5 accessory subunits, which associate primarily with *β*2* nAChR stoichiometries (Grady et al., 2009). A large proportion of the nAChRs in the mHb also contained *β*3 accessory subunits, which were most often found in combination with *β*4* nAChRs (Grady et al., 2009). This contrasts with ventral midbrain dopamine neurons, in which *β*3 subunits are exclusively almost expressed within *α*6* nAChRs. In the IPn, *α*2*β*2*, *α*3*β*2*, *α*4*β*2*, *α*3*β*4*, *α*4*β*4*, and *α*6*β*4* nAChRs have been detected (Grady et al., 2009). Again, a large proportion of these contained *α*5 or *β*3 accessory subunits, with *α*5 subunits associating primarily with *β*2* nAChRs and *β*3 with *β*4* nAChRs (Grady et al., 2009) (Fig. 4).

**Fig. 5:**
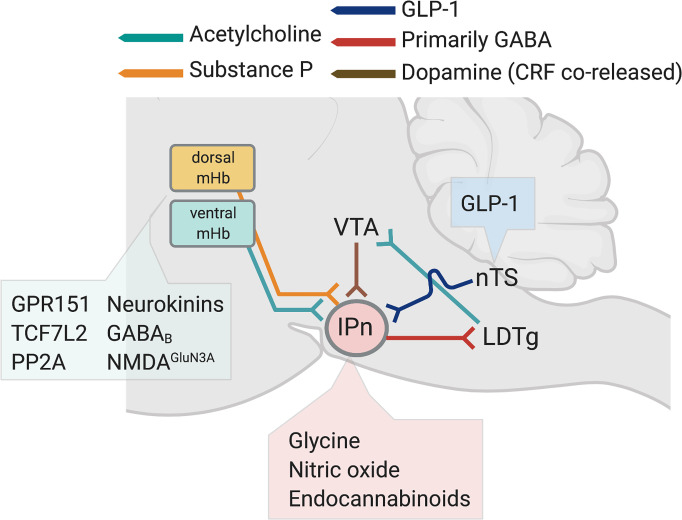
Habenula-interpeduncular mechanisms of nicotine aversion. Neurons in dorsal and ventral mHb express genes implicated in nicotine aversion and other aversion-related behavioral states. These genes include the orphan G-protein coupled receptor (GPCR) GPR151, the transcription factor TCF7L2, the phosphatase PP2A, neurokinins and their receptors, GABA_B_ receptors, and NMDA receptors that contain NR3A subunits. The IPn receives inputs from the nTS that release the neuropeptide GLP-1, which facilitates excitatory transmission in the IPn and thereby enhances nicotine aversion. The IPn also receives inputs from VTA neurons that release dopamine and CRF, both of which increase IPn neural activity. Excitatory transmission in the IPn derived from excitatory inputs is regulated by locally released glycine, nitric oxide, and endocannabinoids. The IPn sends an inhibitory projection to the LDTg that inhibits cholinergic projections from the LDTg to the VTA, which results in decreased activity of VTA dopamine neurons. See main text for further details.

As noted above, a large proportion of nAChRs in the mHb contain *α*5 accessory subunits (Grady et al., 2009) and nAChR function in the mHb was markedly reduced in *α*5 subunit knockout mice when measured using rubidium efflux from habenular synaptosomes (Fowler et al., 2011). Habenular synaptosomes generated from *β*2 but not *β*4 knockout mice also showed striking deficits in nAChR function (Grady et al., 2009). These findings suggest that *α*5* nAChRs play an important role in the mHb (Fig. 4). However, *α*5 subunit mRNA transcripts are expressed at very low levels by mHb neurons, with far higher concentrations found in the IPn (Sheffield et al., 2000; Hsu et al., 2013; Morton et al., 2018), although *α*5 nAChR subunit protein is detected in the mHb (Grady et al., 2009). As nAChR signaling in synaptosomes primarily reflects presynaptic nAChRs, these data suggest that *α*4*β*2*α*5* nAChRs are the major functional nAChR subtype expressed on the terminals of synaptic inputs to mHb neurons. Consistent with this notion, nicotine increases the intrinsic excitability of mHb neurons through an action that depends on *α*5* nAChRs contained on presynaptic terminals in the mHb (Dao et al., 2014), with these presynaptic *α*5* nAChRs regulating the release of neurokinins into the mHb, which act at postsynaptic neurokinin receptors located on mHb neurons (Dao et al., 2014).

This raises the important question of which nAChR subtypes into which *α*5 subunits assemble in the mHb-IPn circuit to regulate behavioral responses to nicotine. In heterologous expression systems, *α*5 nAChR subunits can coassemble into *α*4*β*2, *α*3*β*2, and *α*3*β*4 nAChR subtypes (Fucile et al., 1997; Gerzanich et al., 1998; Tapia et al., 2007). However, in the brain *α*5 subunits preferentially assemble into *α*4*β*2* nAChR subtypes and are rarely detected in *α*3* nAChRs (Gotti et al., 2007; Perry et al., 2007; Kuryatov et al., 2008; Mao et al., 2008). Moreover, *α*5 subunits were shown to incorporate far more readily into *β*2* nAChRs than *β*4* nAChR in the mHb-IPn circuit of rats and mice (Grady et al., 2009). In *Xenopus* oocytes, only the function of *α*4*β*2*α*5* nAChRs but not *α*3*β*4*α*5* or *α*3*β*2*α*5* nAChRs was affected by incorporation of mutant *α*5 subunits modified to include the major smoking-related *CHRNA5* risk allele (D398N), as measured by agonist-evoked calcium influx (Kuryatov et al., 2011). This suggests that *α*4*β*2*α*5* nAChRs likely play a major role in regulating the stimulatory actions of nicotine on the mHb-IPn circuit. However, as noted above, pharmacological blockade of *α*3*β*4* nAChRs attenuated the stimulatory effects of nicotine on the mHb-IPn circuit and increased nicotine intake in rats (Glick et al., 2011; Elayouby et al., 2021), whereas overexpression of these receptors in the mHb-IPn circuit increased nicotine aversion and decreased nicotine intake in mice (Frahm et al., 2011; Slimak et al., 2014). Hence, both *α*4*β*2*α*5* and *α*3*β*4* nAChRs in the mHb-IPn circuit appear to regulate nicotine aversion. Nicotine enhances both glutamate and acetylcholine release from habenular terminals in the IPn (Ren et al., 2011), with both neurotransmitters coreleased from the same mHb cholinergic neurons (Ren et al., 2011). Acetylcholine also facilitates the packaging of glutamate into synaptic vesicles in mHb cholinergic neurons, thereby indirectly facilitating glutamatergic transmission in the IPn (Frahm et al., 2015). Discrete firing patterns of habenular cholinergic neurons are required to stimulate glutamate versus acetylcholine release (Ren et al., 2011), with brief optogenetic stimulation sufficient to elicit glutamate release but more persistent (tetanic) optogenetic stimulation required to trigger acetylcholine release (Ren et al., 2011). Intriguingly, *α*3*β*4* nAChRs are thought to exclusively regulate acetylcholine release in the mHb-IPn circuit (Grady et al., 2001; Hussain et al., 2008; Grady et al., 2009), whereas *α*4*β*2*α*5* nAChRs regulate glutamate transmission (Girod et al., 2000). These findings suggest that multiple parallel yet pharmacologically distinct streams of information originate from habenular inputs to the IPn. The first is a glutamate-regulated signal modulated by *α*4*β*2*α*5* nAChRs, and the second is an acetylcholine-regulated signal modulated by *α*3*β*4* nAChRs. In addition, a third stream of information is likely to originate from substance P–expressing neurons in dorsal portions of the mHb that project to the lateral IPn (see below), but remarkably little is known about the function of these substance P cells. How these putatively dissociable signals function together and independently to regulate the motivational properties of nicotine is unknown.

### G. nAChRs in IPn Regulate Nicotine Reward and Aversion

The mHb projects almost exclusively to the IPn via the fasciculus retroflexus (Qin and Luo, 2009), where mHb neurons corelease glutamate and acetylcholine (Ren et al., 2011). Nevertheless, relatively little is known about how IPn neurons regulate behavioral responses to nicotine. Aversive doses of nicotine decrease then increase locomotor activity of rats in a time-dependent manner (Morrison and Stephenson, 1972; Stolerman et al., 1973; Clarke and Kumar 1983a,b). Locomotor depression occurs during the first 15–20 minutes after administration of higher nicotine doses followed by locomotor stimulation that can persist for >60 minutes. The locomotor-depressing effects of nicotine are thought to reflect nicotine-induced malaise and are mediated at least partly by mHb cholinergic neurons (Salas et al., 2004a; Frahm et al., 2015; Antolin-Fontes et al., 2020). Excitotoxic lesion of the IPn attenuated the initial locomotor-depressing but enhanced the later locomotor-stimulating effects of nicotine (Hentall and Gollapudi, 1995). This suggests that stimulation of excitatory inputs from the mHb to the IPn and direct actions on IPn neurons (Mulle et al., 1991; Lena et al., 1993) contributes to aversive behavioral responses to nicotine. Immunoprecipitation techniques have established that significant populations of *β*2* nAChRs that combine with *α*3 or *α*4 subunits and sparse populations of *β*2* nAChRs that combine with *α*6 subunits are contained within the IPn (Grady et al., 2009). High concentrations of *β*4* nAChRs are also detected in the IPn, with the majority containing *α*3 subunits and smaller populations containing *α*4 or *α*6 subunits (Grady et al., 2009). As noted above, knockdown of *α*3 nAChR subunits or pharmacological blockade of *α*3*β*4* nAChRs in the IPn increased nicotine self-administration in rats (Glick et al., 2011; Elayouby et al., 2021). However, blockade of *α*3*β*4* nAChRs in the VTA located bilaterally to the IPn had no effects on nicotine self-administration in rats (Glick et al., 2011). This suggests that *α*3*β*4* nAChRs expressed by IPn neurons regulate the motivational properties of nicotine. High concentrations of *α*5* nAChRs are detected in the IPn (Morton et al., 2018), particularly in rostral, central, and the intermediate regions that receive input from ventral portions of the mHb (Fonck et al., 2009; Shih et al., 2014; Quina et al., 2017). Optogenetic stimulation of *α*5 nAChR-expressing neurons in the IPn elicits aversion-related behaviors in mice, with these behaviors facilitated by pretreatment with nicotine (Morton et al., 2018). Translating ribosomal affinity profiling combined with RNA sequencing has been used to transcriptionally profile *α*5* nAChR-expressing (*α*5^+^) neurons in the IPn (Ables et al., 2017). This analysis identified at least two transcriptionally dissociable and spatially segregated populations of *α*5^+^ neurons (Ables et al., 2017). *α*5^+^ neurons that express *Amigo1* (*α*5^Amigo1^ cells) also coexpress high concentrations of somatostatin and nitric oxide synthase 1, are concentrated rostral nucleus of the IPn, and send efferent input to the median raphe and LDTg (Ables et al., 2017). By contrast, *α*5^+^ neurons that express *Epyc* (*α*5^Epyc^ cells) are concentrated in the intermediate nucleus but also present in the rostral nuclei of the IPn and do not send long-range projections but instead are scattered locally within the IPn and to a lesser extent in the raphe nuclei (Ables et al., 2017). *Amigo1* and *Epyc* encode cell adhesion proteins, the function is poorly understood, but their expression is useful for distinguishing between these subpopulations of *α*5^+^ neurons in IPn. As might be expected, *α*5^Amigo1^ cells concentrated in the rostral IPn, which receives dense input from the ventrolateral mHb, showed robust increases in activity in response to nicotine treatment (Ables et al., 2017). *α*5^Epyc^ cells located in the rostral IPn also showed nicotine-induced increases in activity, whereas those located in the intermediate nucleus were relatively insensitive to nicotine (Ables et al., 2017). This suggests that *α*5^Epyc^ cells are a collection of at least two further subpopulations of *α*5^+^ neurons. Excitatory inputs to the IPn from mHb cholinergic were inhibited by nitric oxide–mediated retrograde signaling presumably derived from the *α*5^Amigo1^ cells (Ables et al., 2017). Furthermore, expression of a genetically encoded toxin to inhibit neurotransmitter release and thereby silence *α*5^Amigo1^ cells attenuated nicotine-induced CPP and decreased oral intake of the drug, whereas silencing of *α*5^Epyc^ cells had no effects of nicotine reward (Ables et al., 2017). shRNA-mediated knockdown of nitric oxide synthase 1 in the IPn also attenuated nicotine reward (Ables et al., 2017). These findings suggest that *α*5^Amigo1^ cells are activated by nicotine and, presumably, by acetylcholine and glutamate derived from mHb inputs from cholinergic neurons in ventral mHb, which results in the generation of nitric oxide–mediated inhibitory feedback inhibition onto mHb inputs to these cells. This feedback inhibition of mHb inputs by *α*5^Amigo1^ cells contributes to the rewarding properties of nicotine. It is notable that the rewarding and not the aversive properties of nicotine were attenuated by silencing of *α*5^Amigo1^ cells (Ables et al., 2017), a finding similar to the attenuated nicotine reward seen in mice after genetic deletion of choline acetyltransferase in mHb cholinergic neurons (Frahm et al., 2015). This suggests that the mHb-IPn circuit not only regulates nicotine aversion but also nicotine reward. The fact that only the aversive properties of nicotine were in impacted in *α*5 nAChR subunit knockout mice suggests that *α*5* nAChR-independent signaling mechanisms in the mHb-IPn circuit regulate nicotine reward (Jackson et al., 2010; Fowler et al., 2011, 2013; Grieder et al., 2017). In addition to *α*3* and *α*5* nAChRs, the IPn also contains the highest concentrations of *α*2 nAChR subunit transcripts in the rodent brain (Wada et al., 1988). Allelic variation in *CHRNA2*, the gene that encodes the *α*2 nAChR subunit, is associated with increased vulnerability to physical dependence on nicotine (Wang et al., 2014) and cannabis (Demontis et al., 2019). *α*2 nAChR subunit knockout mice show higher levels of intravenous nicotine self-administration during the acquisition phase when nicotine-taking behavior is being established but not after stable intake has been established (Lotfipour et al., 2013). In the IPn, *α*2 nAChRs incorporate into in *β*2* nAChRs primarily (Grady et al., 2009). Hence, *α*2*β*2* nAChRs in the IPn are likely to regulate the acquisition of nicotine-taking behaviors.

### H. Efferent Projections from IPn Regulate Nicotine Reward and Aversion

Intra-mHb infusion of the *α*3*β*4* nAChR antagonists 18-MC or *α*-conotoxin AuIB blocked the stimulatory effects of nicotine but not morphine or *d*-amphetamine on dopamine release in the NAc shell (McCallum et al., 2012). Further, dopamine levels were elevated in the accumbens, and VTA dopamine cells appeared more sensitive to the stimulatory effects of nicotine in *β*4 nAChR subunit knockout mice compared with wild-type mice (Harrington et al., 2016). These dopamine-related effects were reversed by virus-mediated re-expression of *β*4 subunits in the IPn of the knockout mice (Harrington et al., 2016). This regulatory action of the mHb-IPn circuit over nicotine-induced increases in accumbal dopamine transmission may explain why manipulations of neurons in the mHb-IPn circuit can modify both the rewarding and aversive properties of nicotine (Jackson et al., 2010; Fowler et al., 2011, 2013; Frahm et al., 2015; Ables et al., 2017; Grieder et al., 2017). Unlike the LHb, the mHb does not provide any direct projections to midbrain dopamine neurons, nor does it project to the accumbens (Aizawa et al., 2012; Viswanath et al., 2014). Similarly, IPn neurons do not send projections to the VTA (Lima et al., 2017; Quina et al., 2017; Metzger et al., 2019). How then does the mHb-IPn circuit regulate dopamine transmission and the reward effects of nicotine? Recent findings have begun to shed some light on this issue. The IPn sends inhibitory and excitatory inputs from anatomically segregated subnuclei to the LDTg (Lima et al., 2017; Quina et al., 2017), a component of which arises from the *α*5^Amigo1^ GABAergic neurons in rostral IPn (Ables et al., 2017) (Fig. 5). IPn inputs to LDTg are predominately GABAergic, as expected considering the majority of IPn neurons synthesize GABA. However, a small component of IPn input to the LDTg is glutamatergic, likely reflecting efferent projections from the sparse populations of neurons in IPn that synthesize glutamate (Quina et al., 2017). The LDTg sends reciprocal GABAergic and cholinergic projections to the IPn (Bueno et al., 2019). Nicotine acts at presynaptic terminals to enhance GABAergic and glutamatergic transmission in the LDTg (Ishibashi et al., 2009), with at least a portion of these responses presumably reflecting the actions of nicotine on IPn terminals in the LDTg. The LDTg also sends cholinergic projections to the VTA that regulate the tonic and phasic activity of local dopamine neurons through LDTg-derived cholinergic transmission acting act *β*2* nAChRs (Lodge and Grace, 2006; Mameli-Engvall et al., 2006; Chen and Lodge, 2013; Faget et al., 2016) (Fig. 5). Hence, LDTg cholinergic projections to the VTA control VTA-derived dopamine release in the accumbens (Blaha et al., 1996; Forster and Blaha, 2000). LDTg cholinergic, GABAergic, and glutamatergic afferents can also bypass the VTA to directly innervate the accumbens (Dautan et al., 2014; Coimbra et al., 2019). These LDTg inputs to the VTA and accumbens play major roles in controlling reward- and aversion-related behaviors (Dautan et al., 2016; Xiao et al., 2016; Steidl et al., 2017b; Coimbra et al., 2019) in a manner that depends on accumbal dopamine transmission (Steidl and Veverka, 2015; Steidl et al., 2017a). Moreover, the LDTg-VTA circuit is known to regulate behavioral responses to nicotine (Alderson et al., 2005; Maskos, 2008; Ishibashi et al., 2009). Hence, the MHb-IPn circuit may influence mesoaccumbens dopamine transmission through an indirect mechanism that involves IPn projections to the LDTg. Consistent with this possibility, nicotine stimulates *β*2* nAChRs on IPn neurons to increase inhibitory GABAergic transmission in LDTg neurons (Wolfman et al., 2018). Moreover, optical stimulation of IPn terminals in the LDTg elicited avoidance behaviors, whereas optical inhibition of the IPn-LDTg circuit attenuated nicotine aversion (Wolfman et al., 2018). These findings suggest that an mHb-IPn-LDTg-VTA circuit regulates nicotine reward and aversion, in part by modulating mesoaccumbens dopamine transmission.

**Fig. 6:**
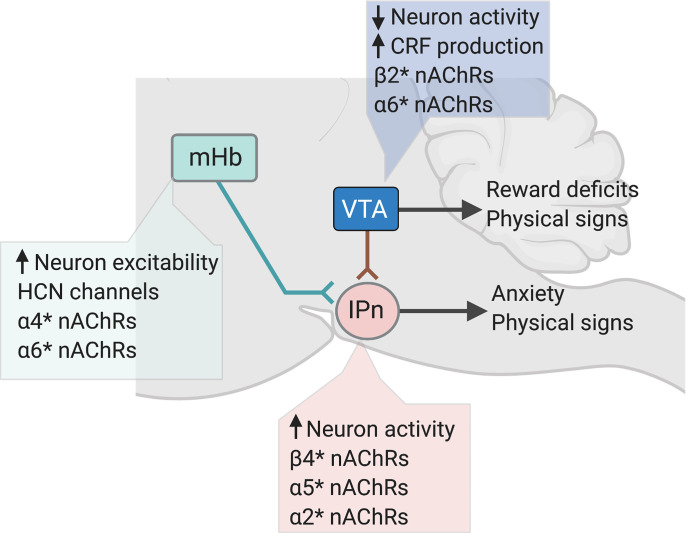
VTA, habenula, and interpeduncular nucleus contributions to nicotine withdrawal. Neurons in ventral mHb show increased excitability after chronic nicotine treatment, and inhibitors of HCN pacemaker channels can precipitate withdrawal in nicotine-dependent animals. In addition, *α*4* and *α*6* nAChR function in the mHb is upregulated in nicotinedependent animals. In the IPn, nicotine withdrawal is associated with increased activity of local GABAergic neurons that express somatostatin, and upregulated expression/function of *β*4*, *α*5*, and *α*2* nAChRs is thought to contribute to the expression of nicotine withdrawal. In the VTA, nicotine withdrawal is associated with decreased activity of dopamine neurons that project to the nucleus accumbens, increased production and release of the stress hormone CRF, and upregulated expression/function of *β*2*, *α*6* nAChRs.

In addition to the LDTg and raphe nuclei, the IPN also projects to other hindbrain sites, including the dorsolateral tegmental nucleus and nucleus incertus (Smaha and Kaelber, 1973; Shibata and Suzuki, 1984; Goto et al., 2001; Olucha-Bordonau et al., 2003). The IPn also provides ascending projections to septal and hippocampal sites, although whether IPn neurons are the source of these ascending projections has been questioned (Quina et al., 2017). There is a small population of serotonergic (5-hydroxytryptophan) neurons in apical regions of the IPn (IPn^5HT^ cells) known to provide ascending input to the hippocampus and septum (Groenewegen and Steinbusch, 1984; Sherafat et al., 2020), which are activated by aversive stimuli (Chen et al., 2003). Chemogenetic inhibition of the IPn^5HT^ cells that project to ventral hippocampus (IPn^5HT^-VH cells) enhanced escape behaviors and increased sucrose intake in mice (Sherafat et al., 2020). However, chemogenetic inhibition of IPn^5HT^-VH cells did not alter responding for intravenous nicotine infusions in mice even when a higher unit dose of nicotine on the descending portion of the dose-response curve was available (Sherafat et al., 2020). This suggests that IPn^5HT^-VH cells are not involved in regulating nicotine intake, although it is unclear whether projections from IPn^5HT^ cells to other addiction-relevant brain sites may be involved. It is important to note that the apical portion of the IPn, which contains serotonergic and other ascending projections to limbic brain regions is functionally and anatomically distinct from the IPn proper (Quina et al., 2017). It has been argued that this apical region may in fact represent an extended portion of the median raphe nucleus (Quina et al., 2017). If so, the serotonergic neurons in apical IPn regions may not be participate in behaviors directly regulated by the mHb-IPn circuit. As noted above, the IPn sends dense projections to the nucleus incertus in the hindbrain. Indeed, it was shown that *α*5^+^ IPn neurons send GABAergic projections to the nucleus incertus (Morton et al., 2018), and the sparse population of IPn glutamatergic neurons also project to this site (Quina et al., 2017). Nucleus incertus neurons in turn project to the hippocampus and elsewhere in the brain (Goto et al., 2001; Szőnyi et al., 2019). Optogenetic stimulation of nucleus incertus neurons that project to hippocampus blocked the encoding of fear memories in mice, whereas their inhibition markedly enhanced the encoding of fear memories (Szőnyi et al., 2019). This suggests that bidirectional modulation of nucleus incertus activity by inhibitory and excitatory inputs from the IPn potentially contributes to the rewarding and aversive actions of nicotine, respectively, a possibility that has not yet been explored.

### I. Afferent Projections to IPn Regulate Nicotine Reward and Aversion

The IPn receives inputs from brain sites other than the mHb known to encode reward and aversion states and likely to influence behavioral responses to nicotine. Recent evidence suggests that dopamine neurons in the VTA project to the IPn (VTA-IPn neurons) (Molas et al., 2017; DeGroot et al., 2020) (Fig. 5). Optogenetic stimulation of VTA-IPn neurons decreased anxiety-related behaviors in mice (DeGroot et al., 2020). Similarly, infusion of a D1 dopamine receptor agonist into the IPn decreased anxiety-related behaviors whereas a D1 receptor antagonist had the opposite effect (DeGroot et al., 2020). Hence, nicotine acting on VTA dopamine neurons that project to the IPn, potentially through *α*5* nAChRs expressed by these cells, may contribute to its well known anxiety-promoting actions and other aversive responses that promote nicotine avoidance (File et al., 1998, 2000; Ouagazzal et al., 1999a,b; Kenny et al., 2000). In addition to the mHb-IPn circuit, *α*5, *α*3, and *α*4 nAChR subunit transcripts are also densely expressed by neurons in the nTS. Nicotine dose-dependently activates nTS neurons, as reflected by increases in cFos immunoreactivity, with this effect most apparent in nTS neurons that express the neuropeptide glucagon-like peptide-1 (GLP-1) (Tuesta et al., 2017). Using a line of *Chrna5*-tdTomato reporter mice, GLP-1 neurons were shown to express *α*5 nAChR subunits (Tuesta et al., 2017). Systemic administration of the GLP-1 receptor agonist exendin-4 or the dipeptidyl peptidase 4 inhibitor sitagliptin, which inhibits GLP-1 breakdown, decreased nicotine self-administration in mice (Tuesta et al., 2017). Using transgenic mice that express Cre recombinase in GLP-1 neurons in nTS, it was shown that chemogenetic stimulation of these cells reduced nicotine self-administration (Tuesta et al., 2017). Conversely, GLP-1 receptor knockout mice self-administered greater quantities of nicotine than their wild-type littermates (Tuesta et al., 2017). Some of the highest densities of GLP-1 receptor binding sites in the brain are detected in the IPn (Goke et al., 1995), suggesting that GLP-1–expressing nTS neurons project to the IPn and thereby regulate nicotine intake. Indeed, GLP-1–immunoreactive fibers were detected in the IPn of mice. Furthermore, optical stimulation of the terminals of GLP-1 neurons in the IPn enhanced local excitatory transmission through a mechanism involving increased glutamate release from mHb inputs (Tuesta et al., 2017) (Fig. 5). Consistent with this mechanism of action, infusion of exendin-4 into the IPn or habenula decreased nicotine intake, whereas IPn infusion of the GLP-1 receptor antagonist exendin-(9-39)-amide or shRNA-mediated knockdown of GLP-1 receptor mRNA in the mHb-IPn circuit increased nicotine self-administration in rats (Tuesta et al., 2017). GLP-1 neurons are known to be activated by gastric expansion to induce feelings of satiety and trigger cessation of food consumption (van Bloemendaal et al., 2014). GLP-1 neurons also regulate feelings of malaise and nausea when food is consumed past satiety (Chang and Scott, 1984; Hayama et al., 1985; Scott et al., 1986; Garcia-Diaz et al., 1988; Monnikes et al., 1997; Willing and Berthoud, 1997; Rinaman et al., 1998; Gutzwiller et al., 1999; Schwartz, 2000; Qin et al., 2005; Appleyard et al., 2007; Bello and Moran, 2008). Hence, it was proposed that activation of GLP-1 neurons by nicotine engages IPn circuits to elicit satiety-like responses to the drug that promote nicotine avoidance behaviors and thereby terminate nicotine self-administration (Tuesta et al., 2017).

### J. Other Reward and Aversion Signaling Mechanisms in the mHb-IPn Circuit

In addition to nAChRs, other receptors and signaling mechanisms in the mHb-IPn circuit have been shown to regulate behavioral responses to nicotine. As described above, GLP-1 facilitates excitatory transmission in the mHb-PN circuit to promote nicotine avoidance behaviors (Tuesta et al., 2017). GLP-1 receptors are G-protein–coupled receptors (GPCRs) that, when activated, enhance the production of cAMP. In pancreatic *β* cells, GLP-1–enhanced cAMP signaling results in the phosphorylation and nuclear translocation of *β*-catenin, which dimerizes with the transcription factor TCF7L2, and TCF7L2 is considered a core component the GLP-1 signaling cascade (Yi et al., 2005; Liu and Habener, 2008; Vazquez-Roque et al., 2011; Chiang et al., 2012; Shao et al., 2013). TCF7L2 is densely expressed in mHb cholinergic neurons (Duncan et al., 2019). Using a line of genetically modified rats that express a loss-of-function TCF7L2 variant, it was shown that TCF7L2 deficiency markedly reduced aversive behavioral responses to nicotine and increased nicotine self-administration behavior (Duncan et al., 2019). Similarly, CRISPR/CRISPR-associated protein 9–mediated genomic cleavage of the *Tcf7l2* gene in the mHb of mice or shRNA-mediated knockdown of TCF7L2 mRNA transcripts in the mHb of rats increased nicotine self-administration behavior (Duncan et al., 2019). The stimulatory effects of nicotine on mHb inputs to the IPn were diminished in the TCF7L2-deficient rats, as reflected by reductions in mHb-derived excitatory transmission in IPn slices evoked by nicotine (Duncan et al., 2019). The diminished sensitivity of the mHb-IPn circuit to nicotine was explained by a deficit in the function but not the expression of nAChRs in mHb neurons (Duncan et al., 2019). Deficient TCF7L2-mediated transcriptional activity in the mHb of the TCF7L2 mutant rats resulted in striking deficits in the production of cAMP in mHb neurons, which reduced activity of the cAMP-regulated protein kinases that positively regulate nAChR function in mHb neurons (Huganir et al., 1986; Paradiso and Brehm, 1998; Giniatullin et al., 2005). This abnormality in cAMP signaling explained the deficits in habenular nAChR function and the elevated nicotine intake seen in TCF7L2-deficient animals (Duncan et al., 2019). These findings highlight the importance of cAMP and likely other intracellular signaling processes that regulate nAChR function in the mHb-IPn pathway in the reinforcing actions to nicotine.

GPR151 is orphan GPCR that is highly enriched in habenular neurons in rodents and humans, particularly mHb neurons that coexpress *α*3* nAChRs (Quina et al., 2009; Kobayashi et al., 2013; Broms et al., 2015; Wagner et al., 2016; Broms et al., 2017; Antolin-Fontes et al., 2020). The stimulatory effects of nicotine on mHb neurons are attenuated in GPR151 knockout mice (Antolin-Fontes et al., 2020). The knockout mice also had attenuated aversive behavioral responses to nicotine and self-administered greater numbers of intravenous nicotine infusions than wild-type mice, particularly when a higher unit dose on the descending portion of the dose-response curve was available (Antolin-Fontes et al., 2020). GPR151 appears to be negatively coupled to adenylyl cyclase activity, as levels of cAMP were elevated in mHb tissue collected from GPR151 knockout mice compared with wild-type mice (Antolin-Fontes et al., 2020). Hence, GPR151 may modulate behavioral responses to nicotine in a manner similar to TCF7L2 by regulating the cAMP-dependent kinases that control nAChR function in the mHb-IPn circuit. GPR151 is thought to be expressed almost exclusively on the terminals of mHb neurons in the IPn (Antolin-Fontes et al., 2020). Hence, GPR151 may influence nicotine intake by regulating the function of presynaptically located nAChRs on mHb terminals and the neurotransmitter release machineries in mHb terminals known to be controlled by cAMP-dependent signaling processes (Azhderian et al., 1994; Chen and Regehr, 1997; Duncan et al., 2019).

GPR139 is another orphan GPCR that is densely expressed in the mHb and is also found in the LHb, septum, and striatum (Liu et al., 2015; Wagner et al., 2016). Allelic variation in the *GPR139* gene influences body mass index and blood pressure in a manner that depends on smoking status (current smoker, ex-smoker, or nonsmoker) (Justice et al., 2017; Sung et al., 2018). GPR139 modulates *μ* opioid receptor function in the mHb to regulate the rewarding actions of opioids (Wang et al., 2019) through a mechanism that likely involves regulation of cAMP signaling dynamics in mHb neurons (Stoveken et al., 2020). The role for GPR139 in regulating the motivational properties of nicotine has not yet been explored. However, the novel GPR139 agonist TAK-041 blocks the stimulatory effects of nicotine on accumbal dopamine release (Schiffer et al., 2020), suggesting that GPR139 located in the mHb-IPn circuit and striatum may modulate the rewarding and aversive actions of nicotine, a possibility that awaits further investigation.

Phosphodiesterase 2A (PDE2A) metabolizes cAMP and is robustly expressed by neurons in the mHb and IPn as well as the hippocampus, striatum, globus pallidus, and substantia nigra (Stephenson et al., 2009, 2012). Atrial natriuretic peptide activates PDE2A in mHb neurons, thereby inhibiting the release of glutamate into the IPn (Hu et al., 2012). This inhibitory action of atrial natriuretic peptide on mHb-derived glutamatergic transmission in the IPn was prevented by PDE2A inhibition or by a chemical activator of cAMP-regulated protein kinase A (Hu et al., 2012). These findings further highlight the importance of cAMP signaling in regulating mHb-IPn circuit function and suggest that PDE2A modulators that influence mHb-IPn circuit responses to nicotine may represent a novel class smoking-cessation therapeutics.

As described above, *α*5*β*2* nAChRs enhance neurokinin signaling in the mHb, which increases the intrinsic excitability of mHb neurons (Dao et al., 2014). The source of neurokinins in this action is unclear but may derive from noncholinergic neurons in dorsal mHb that synthesize substance P (neurokinin A) and project to lateral IPn (Contestabile et al., 1987; Qin and Luo, 2009; Aizawa et al., 2012; Melani et al., 2019). Substance P–expressing mHb neurons in dorsal mHb that project to lateral IPn also express CB_1_, GABA_B_, and glycine receptors, all of which regulate the encoding or extinction of fear memories (Soria-Gomez et al., 2015; Zhang et al., 2016; Melani et al., 2019) (Fig. 5). Stimulation of neurons in dorsal mHb neurons increased excitatory glutamatergic transmission in the lateral IPn, which in turn stimulated local inhibitory glycine-containing neurons in the IPn (Melani et al., 2019) (Fig. 5). Glycine released in this manner is thought to serve a negative feedback role by counterbalancing the local actions of mHb-derived glutamate and thereby opposing glutamate-driven long-term synaptic potentiation in the IPn (Melani et al., 2019). Strychnine-mediated inhibition of glycinergic transmission in lateral IPn disrupted this counter-regulatory response and enhanced the release of glutamate from mHb terminals, which in turn facilitated the induction of long-term synaptic potentiation at the dorsal mHb-lateral IPn synapse (Melani et al., 2019). This synaptic potentiation was blocked by neurokinin 1 receptor antagonism (Melani et al., 2019), suggesting that glycine released from lateral IPn neurons acts by inhibiting substance P and glutamate corelease from mHb terminals in the IPn (Melani et al., 2019). Injection of a neurokinin 1 receptor antagonist into lateral IPn, which blocked the actions of substance P released by dorsal mHb inputs, impaired the ability of mice to extinguish fear memories (Melani et al., 2019), suggesting that dorsal mHb–lateral IPn synapse controls the processing and gradual extinction of behavioral responses to aversive stimuli. Notably, CB_1_ receptor antagonism also blocked activity-dependent synaptic potentiation at the dorsal mHb-lateral IPn synapse, an effect mimicked by a GABA_B_ receptor antagonist (Melani et al., 2019) (Fig. 5). Conditional deletion of CB_1_ receptors from mHb neurons decreased fear-conditioned freezing behavior and abolished conditioned odor aversion in mice but did not alter behavioral responses to neutral or appetitive stimuli Soria-Gomez et al. (2015). These findings suggest that glycine, endocannabinoid, and neurokinin transmission in the IPn regulate the acquisition, expression, and extinction of behavioral responses to aversive stimuli (Fig. 5). Interestingly, mHb neurons express an uncommon NMDA receptor subtype that contains GluN1/GluN3A subunits, which are activated by glycine (Otsu et al., 2019). Activation of these GluN1/GluN3A NMDA receptors by glycine increases the activity of mHb neurons, which triggers avoidance-related behaviors (Otsu et al., 2019) (Fig. 5). It is likely that glycine, endocannabinoid, and neurokinin transmission in lateral IPn regulate aversive responses to nicotine in a manner that involves GluN1/GluN3A NMDA receptors on the terminals of mHb neurons. This possibility has not yet been explored.

### K. Nicotine Actions in the Periphery Impact Reward and Aversion Behaviors

The nTS is the major site to which the vagus nerve (cranial nerve X) delivers sensory information to the brain from peripheral organs involved in processing the interoceptive actions of nicotine contained in cigarette smoke (Ogawa et al., 1984; Hines et al., 1994). This raises the possibility that nicotine acts not only at nAChRs located directly in the nTS but also at nAChRs located in peripheral organs, with nicotine-related sensory information contributing to nTS activation and nicotine avoidance behaviors. Indeed, nAChRs are expressed in tissues that come into direct contact with nicotine in tobacco smoke, including nAChRs in the mouth and lungs. Sensory information related to the actions of nicotine in the oral cavity, airways, and elsewhere in the body (Cain, 1980; Ahijevych et al., 2015) activate nTS neurons through vagal inputs (Lemon and Smith, 2005; Simons et al., 2006). Nicotine-related sensory information is then relayed from the nTS to higher-order midbrain and corticothalamic circuits involved in reward and avoidance behaviors. This nicotine-related sensory information contributes to the interoceptive properties of tobacco smoke that smokers use to titrate their intake to avoid its aversive properties (Iwasaki and Sato, 1981; Feyerabend et al., 1982; Rose et al., 1993; Pritchard et al., 1996; Dahl et al., 1997; Ming et al., 1998; Fowler and Kenny, 2014). Nicotine acting peripherally can also precipitate craving in smokers during periods of abstinence (Naqvi et al., 2007; Gray and Critchley, 2007; Naqvi and Bechara, 2010; Fowler and Kenny, 2014). Moreover, blocking the sensory properties of nicotine in cigarette smokers attenuates the rewarding properties of cigarettes and decreases smoking behavior (Rose et al., 1985, 1993, 2016; McClernon et al., 2016). In rodents, actions of nicotine outside the brain contribute to many of the behavioral and physiologic responses to the drug (Kiyatkin, 2014). Despite the considerable evidence linking peripheral actions of nicotine to tobacco use disorder, remarkably little is known about the underlying mechanisms of such brain-body interactions. However, a number of groups have used the modified nicotine analog nicotine pyrrolidine methiodide (nicotine-PM), which is a full nAChR agonist that does not cross the blood-brain barrier (Lenoir and Kiyatkin, 2011; Kiyatkin, 2014) to map brain circuits and cells activated by nicotine acting at peripheral organs (Dehkordi et al., 2015; Rose et al., 2016). It was found that nicotine and nicotine-PM increased cFos immunoreactivity in overlapping brain regions, including the IPn and other sites involved in the reward and aversion, such as the LDTg, PPTg, LHb, accumbens, and VTA (Dehkordi et al., 2015; Rose et al., 2016). Within the VTA, GABAergic but not dopaminergic cells were almost exclusively activated by a higher aversive but not by a lower rewarding dose of nicotine (Dehkordi et al., 2018), suggesting that these cells may transduce aversive sensory properties of peripherally acting nicotine. Further studies will be required to explore this possibility and to determine whether nTS inputs to VTA IPn, LDTg, PPTg, LHb, or accumbens explain their sensitivity to the peripheral actions of nicotine.

## VI. Mechanisms of Nicotine Withdrawal

### A. Nicotine Withdrawal Syndrome in Humans and Rodents

Tobacco use disorder depends not only on the rewarding properties of nicotine that underlie its positive reinforcing properties but also on escape from the aversive consequences of nicotine withdrawal that motivate tobacco smoking according to negative reinforcement processes (Doherty et al., 1995; Kenny and Markou, 2001). Chronic nicotine exposure results in the development of a dependent state in smokers such that cessation of intake elicits an aversive nicotine withdrawal syndrome (Shiffman and Jarvik, 1976; Hughes et al., 1991). This withdrawal syndrome is attenuated by nicotine replacement therapy (Schneider and Jarvik, 1984; Fagerstrom et al., 1993; Molander et al., 2000), confirming that nicotine plays a major role in its induction and expression. Conversely, smoking cigarettes with reduced nicotine content can elicit a withdrawal syndrome in smokers that is accompanied by a reduction in plasma nicotine levels (West et al., 1984). The duration and severity of the nicotine withdrawal syndrome in abstinent smokers accurately predict their likelihood of relapse to tobacco use (Piasecki et al., 1998, 2000, 2003). Conversely, the efficacy of nicotine replacement therapy in certain individuals is related to its ability to prevent the onset and reduce the duration of nicotine withdrawal (Fagerstrom, 1988; Sachs and Leischow, 1991). Hence, it is important to understand the brain mechanisms that regulate nicotine withdrawal–associated aversive states.

Nicotine withdrawal in abstinent smokers is comprised of physical and affective components. The most common physical symptoms include bradycardia and gastrointestinal discomfort, suggesting that autonomic nervous system function is disrupted in smokers during withdrawal (Niedermaier et al., 1993). Affective symptoms primarily include irritability, depressed mood, anxiety, difficulty concentrating, and craving (Parrott, 1993; Doherty et al., 1995; Kenny and Markou, 2001). Escape from affective components of withdrawal is thought to play a particularly important role in the maintenance nicotine dependence (Koob and Le Moal, 1997; Markou et al., 1998; Kenny and Markou, 2001). Nicotine withdrawal is associated with physical and affective abnormalities in laboratory rodents. Withdrawal from nicotine and other drugs of abuse is thought to reflect compensatory adaptations in the same neurobiological substrates that regulate acute responses to the drug (Koob and Bloom, 1988; Koob and Le Moal, 1997). Thus, it is likely that the same nAChRs that regulate the rewarding and aversive effects of acutely administered nicotine undergo adaptations upon prolonged exposure to nicotine, which contributes to the development of nicotine dependence and the expression of withdrawal-associated physical and affective components of nicotine withdrawal (Kenny and Markou, 2001).

### B. β2* nAChRs Regulate Affective but Not Physical Components of Nicotine Withdrawal

Spontaneous withdrawal from nicotine, accomplished by the surgical removal of subcutaneously implanted osmotic minipumps that delivered nicotine continuously for >7 days, was associated with the expression of a physical withdrawal syndrome in rats (Malin et al., 1992; Epping-Jordan et al., 1998). Systemic administration of mecamylamine precipitated physical withdrawal signs in nicotine-dependent rats (Watkins et al., 2000). Systemic or intracerebroventricular administration of the nAChR antagonist chlorisondamine, which does not readily cross the blood-brain barrier, also precipitated physical withdrawal signs in nicotine-dependent rats (Watkins et al., 2000). These findings suggest that nAChRs in the brain regulate physical withdrawal from nicotine. They also suggest that peripheral ganglionic nAChRs located outside the brain contribute to the physical withdrawal syndrome. Indeed, expression of physical withdrawal signs in rats undergoing spontaneous nicotine withdrawal were attenuated by the peripherally acting nAChR agonist tetramethylammonium (Hildebrand et al., 1997). By contrast, *β*2* nAChR-preferring antagonist DH*β*E did not precipitate physical withdrawal signs in nicotine-dependent rats (Epping-Jordan et al., 1998). This suggests that *β*2* nAChRs in the brain are unlikely to contribute to physical dependence on nicotine (see Fig. 6).

In addition to a physical syndrome, spontaneous and antagonist-precipitated nicotine withdrawal is also associated with an affective withdrawal syndrome in rats and mice, which can be measured by elevations of ICSS reward thresholds, the induction of a conditioned place avoidance (CPA), decrements in sucrose consumption, increases in anxiety-related behaviors, and other behavioral abnormalities consistent with the manifestation of a negative affective state (see Kenny and Markou, 2001; Fowler et al., 2008). In nicotine-dependent rats, spontaneous withdrawal or withdrawal precipitated by mecamylamine or DH*β*E, elevated ICSS thresholds (Ivanova and Greenshaw, 1997; Epping-Jordan et al., 1998; Watkins et al., 2000; Harrison et al., 2002). Similarly, systemic administration of chlorisondamine at doses unlikely to cross the blood-brain barrier or its direct intracerebroventricular administration also elevated ICSS thresholds in nicotine-dependent rats (Epping-Jordan et al., 1998; Watkins et al., 2000). This suggests that nAChRs located in brain reward circuits, including *β*2* nAChRs, are contribute to the development of “affective dependence” on nicotine. Surprisingly, ganglionic nAChRs located outside the brain may also contribute to this process, although very little is known about the contribution of peripherally located nAChRs to affective components of withdrawal.

*β*2* nAChR subunit knockout mice, which are insensitive to the rewarding properties of nicotine (see above), demonstrated a robust physical nicotine withdrawal syndrome (Besson et al., 2006), but withdrawal-induced elevations in ICSS thresholds were absent in these animals (Stoker et al., 2015). Withdrawal-induced increases in anxiety-related behaviors and the establishment of a CPA for an environment paired with antagonist-precipitated nicotine withdrawal were also absent in *β*2 knockout mice but readily apparent in wild-type mice (Jackson et al., 2008). Deficits in fear conditioning typically observed in wild-type mice experiencing nicotine withdrawal were also attenuated in *β*2 knockout mice (Portugal et al., 2008; Raybuck and Gould, 2009). Most recently, it was shown that *β*2 subunit knockout mice were resistant to nicotine withdrawal-induced deficits in sucrose consumption and increases in anxiety-related behaviors (Alkhlaif et al., 2017). Hence, *β*2* nAChRs regulate affective but not physical signs of nicotine withdrawal (see Fig. 6).

### C. Other nAChR Subtypes Contribute to Affective Components of Nicotine Withdrawal

Similar to *β*2 nAChR subunit knockout mice, *β*3 and *α*6 nAChR subunit knockout mice showed attenuated sucrose drinking and anxiety-related behavioral abnormalities during nicotine withdrawal, whereas physical withdrawal signs were unaltered in these animals (Alkhlaif et al., 2017; Jackson et al., 2019). Furthermore, an *α*6* nAChR-selective antagonist blocked the expression of a nicotine withdrawal-induced CPA and withdrawal-associated increases in anxiety-related behavior but did not modify the expression of physical withdrawal signs in mice (Jackson et al., 2009). These findings suggest that the same *α*6*β*2*β*3* nAChR subtype known to regulate the stimulatory effects of nicotine on striatal dopamine release (see above) is likely to undergo nicotine-induced adaptations in function and expression during chronic nicotine exposure, which contributes to the development of affective but not physical dependence on nicotine. The role for *α*7 nAChRs in affective components of nicotine withdrawal is less clear. Deficits in context-related encoding of fear memories typically seen in mice experiencing nicotine withdrawal were unaltered in *α*7 knockout mice, whereas similar withdrawal-related cognitive deficits were absent in *β*2 subunit knockout mice (Portugal et al., 2008). The *α*7 nAChR antagonist MLA did not precipitate a CPA or other affective signs of withdrawal in nicotine-dependent mice (Markou and Paterson, 2001; Jackson et al., 2008). Nor did MLA precipitate withdrawal-related cognitive deficits in nicotine-dependent rats (Shoaib and Bizarro, 2005). The novel *α*7 nAChR partial agonist encenicline did not attenuate nicotine withdrawal-associated cognitive deficits in abstinent human smokers, as reflected by similar deficits in treated and untreated abstinent smokers when tested using the Conners Continuous Performance Task to assess attention and response inhibition and using the N-Back task to measure working memory (Schuster et al., 2018). Also, the *α*7 nAChR agonist ABT-107 did not attenuate cognitive deficits (encoding of fear memories) detected in mice undergoing nicotine withdrawal (Yildirim et al., 2015). However, the *α*7 nAChR agonist PNU282987 attenuated nicotine withdrawal–induced increases in anxiety-related behavior in mice (Jackson et al., 2018). Furthermore, the deficits in attention typically seen in wild-type mice undergoing nicotine withdrawal were absent in *α*7 knockout mice (Higa et al., 2017). Infusion of MLA into the VTA of nicotine-dependent rats decreased accumbal dopamine release in a manner similar to mecamylamine infusion (Nomikos et al., 1999), suggesting that *α*7 nAChRs may contribute to deficits in dopamine transmission during nicotine withdrawal (Nomikos et al., 2000). Together, these findings suggest that *α*7 nAChRs may play a role in affective components of nicotine withdrawal, but its precise contributions remain unclear (see Fig. 6).

### D. Dopamine Transmission Contributes to Affective Components of Nicotine Withdrawal

Consistent with a role for mesoaccumbens dopamine transmission in nicotine withdrawal, extracellular dopamine levels were markedly reduced in rats undergoing nicotine withdrawal compared with nicotine-naïve animals (Hildebrand et al., 1998), opposite to the stimulatory effects of acutely administered nicotine on accumbal dopamine release (see above). Nicotine withdrawal–related deficits in accumbens dopamine release are less severe in adolescent than adult rats (Natividad et al., 2010), which may explain the attenuated withdrawal-related behaviors seen in adolescent versus adult rats (O’Dell et al., 2004, 2007). Mecamylamine delivered into the VTA decreased extracellular dopamine levels in the accumbens and precipitated withdrawal-like physical signs in nicotine-dependent but not nicotine-naïve control rats (Hildebrand et al., 1999). Similarly, infusion of DH*β*E into the VTA (Bruijnzeel and Markou, 2004) but not the central nucleus of the amygdala or bed nucleus of the stria terminalis (Jonkman and Markou, 2006) precipitated withdrawal-associated elevations of ICSS thresholds in nicotine-dependent but not control rats. Hence, the VTA serves as a key neuroanatomical substrate in the regulation of affective components of nicotine withdrawal.

In contrast to the decreased dopamine release detected in the NAc during nicotine withdrawal, dopamine levels were dramatically increased in the mPFC during withdrawal (Carboni et al., 2000a). This effect is similar to the increased cortical dopamine transmission seen in rodents undergoing withdrawal from opioids (Bozarth, 1994; Bassareo et al., 1995; Espejo et al., 2001). Hence, it is unlikely that the reward deficits and other affective components of withdrawal from nicotine and other major drugs of abuse reflect a generalized state of depressed activity of midbrain dopamine neurons. Instead, the activity of some populations of dopamine neurons are decreased, whereas the activity of others is increased. This likely reflects the same type of partitioning of withdrawal-relevant dopamine neurons in the VTA into functional domains as described above for the reward-related actions of nicotine. Hence, it is an interesting possibility that withdrawal from nicotine and other drugs of abuse is associated with decreased activity of VTA^LAT^ dopamine neurons that project to the NAc shell and increased activity of VTA^MED^ dopamine neurons that project to the mPFC.

A population of dopamine neurons that express corticotropin-releasing factor (CRF) has been identified in the posterior VTA (Grieder et al., 2014). Chronic nicotine treatment upregulated CRF expression in these VTA dopamine neurons, which blocked the stimulatory effects nicotine on local GABAergic transmission in the VTA (Grieder et al., 2014). Blockade of CRF signaling in the VTA during nicotine withdrawal restored the stimulatory actions of nicotine on VTA GABA transmission and attenuated affective components of nicotine withdrawal in rats (Grieder et al., 2014). Notably, the majority of these CRF-positive neurons were located in posterior regions of the VTA immediately adjacent to the IPn (Grieder et al., 2014). Moreover, the increased CRF mRNA levels detected in the VTA of nicotine-dependent rats coincided with decreased CRF immunoreactivity in the IPn of the same animals (Grieder et al., 2014). This suggests that posterior VTA neurons that synthesize CRF may project to the IPn, a circuit known to regulate anxiety-related behaviors in rodents and release CRF into the IPn to drive affective components of nicotine withdrawal. Consistent with this possibility, it was shown that increased anxiety-related behaviors in mice during nicotine withdrawal were associated with increased activity of neurons located in the intermediate nucleus of the IPn, in which *α*5^Epyc^ neurons are known to be located (see above) (Zhao-Shea et al., 2015). This effect was regulated by CRF derived from VTA inputs (Zhao-Shea et al., 2015). Furthermore, blockade of CRF transmission in or optogenetic inhibition of mHb inputs to the intermediate nucleus of the IPn attenuated nicotine withdrawal–induced increases in anxiety-related behavior (Zhao-Shea et al., 2015). Further supporting an important role for the IPn in nicotine withdrawal-related increases in anxiety-related behavior, it was shown that chronic nicotine treatment increased the sensitivity of nAChRs in the mHb to nicotine (Pang et al., 2016; Arvin et al., 2019), with habenular *α*4* and *α*6* nAChRs located in the ventroinferior portion of the mHb being particularly sensitive to this action (Shih et al., 2015; Pang et al., 2016). Moreover, pharmacological blockade of *α*4*β*2* or *α*6*β*2* nAChRs but not *α*3*β*4* nAChRs in the mHb alleviated nicotine withdrawal-related increased in anxiety-related behaviors in mice (Pang et al., 2016). Most recently, it was shown that a history of intravenous nicotine self-administration similarly upregulated nAChR function in the mHb of rats (Jin et al., 2020). Finally, it was shown that a metabotropic glutamate 2/3 receptor antagonist infused systemically or directly into the VTA attenuated the elevations of ICSS thresholds in rats undergoing spontaneous nicotine withdrawal (Kenny et al., 2003). These findings suggest that adaptive responses in *β*2*, *β*3*, and *α*6* nAChRs in the VTA, leading to alterations in dopamine, CRF, GABA, and glutamate signaling in the accumbens, IPn, and other reward and aversion relevant brain sites, drives the expression of affective but not physical components of nicotine withdrawal.

### E. β4* nAChRs Regulate Physical but Not Affective Components of Nicotine Withdrawal

*β*4* nAChRs appear to regulate physical not affective signs of nicotine withdrawal, opposite to the contributions of *β*2* nAChRs described above. Indeed, *β*4 nAChR subunit knockout mice had decreased physical signs in response to spontaneous or mecamylamine-precipitated nicotine withdrawal (Salas et al., 2004b; Stoker et al., 2012). However, mecamylamine-precipitated nicotine withdrawal elevated ICSS thresholds by a similar magnitude in wild-type and *β*4 subunit knockout mice (Stoker et al., 2012), although the onset of threshold elevations during spontaneous nicotine withdrawal was delayed in *β*4 knockout mice relative to wild-type mice (Stoker et al., 2012). Similar to *β*4 knockout mice, *α*5 subunit knockout mice also demonstrated a loss of nicotine withdrawal–induced physical withdrawal signs compared with wild-type mice (Jackson et al., 2008; Salas et al., 2009). By contrast, withdrawal-induced increases in anxiety-related behaviors were unaltered in *α*5 knockout mice (Jackson et al., 2008). The role for *α*2 nAChRs in nicotine withdrawal is complex, however, as one study reported that physical withdrawal signs were attenuated in *α*2 nAChR subunit knockout mice (Salas et al., 2009), and another reported that physical withdrawal was exacerbated in these animals (Lotfipour et al., 2013). The role for *α*7 nAChRs in physical aspects of nicotine withdrawal is also unclear. Some studies have shown that physical withdrawal signs were attenuated in *α*7 subunit knockout mice (Salas et al., 2007), whereas others have shown no difference between knockout and wild-type mice (Stoker et al., 2012). The *α*7 nAChR antagonist MLA did not precipitate physical signs of withdrawal in nicotine dependent mice (Markou and Paterson, 2001; Jackson et al., 2008). However, the *α*7 nAChR agonist PNU282987 attenuated nicotine withdrawal–induced increases in anxiety-related behaviors and physical withdrawal signs in mice (Jackson et al., 2018). Overall, these findings suggest that *β*4* and *α*5* nAChRs and perhaps *α*2* and *α*7 nAChRs regulate physical dependence on nicotine.

### F. nAChR Signaling in mHb-IPn Circuit Regulates Nicotine Withdrawal

Considering the high concentrations of *β*4, *α*5, and *α*2 nAChR subunits in the mHb-IPn circuit, it is perhaps not surprising that this circuit has been implicated in physical components of the nicotine-withdrawal syndrome. Direct infusion of mecamylamine into the mHb or IPn but not the cortex, VTA, or hippocampus precipitated physical withdrawal signs and increased anxiety-related behaviors in nicotine-dependent mice (Salas et al., 2009; Zhao-Shea et al., 2013). Mecamylamine infused into the IPn also increased physical signs of withdrawal and anxiety-related behaviors in nicotine-dependent male and female rats (Correa et al., 2019). The onset of nicotine withdrawal was associated with increased activity of IPn GABAergic neurons, as reflected by increased cFos expression and spontaneous excitatory currents in IPn neurons (Zhao-Shea et al., 2013). Pharmacological blockade of mHb-derived glutamatergic transmission in the IPn attenuated, whereas the optical stimulation of IPn GABAergic neurons precipitated, withdrawal-related physical signs in mice (Zhao-Shea et al., 2013). Chronic nicotine treatment upregulated the expression of *β*4 nAChR subunits in somatostatin-expressing IPn neurons in dorsal portions of the IPn (Zhao-Shea et al., 2013), which may reflect the *α*5^Amigo^ cells that regulate nitric oxide–mediated retrograde inhibition of mHb inputs to the IPn (see above) (Ables et al., 2017). This suggests that adaptions in *β*4* nAChR signaling in the IPn contribute to the development of nicotine dependence. Consistent with this interpretation, infusion of the *α*3*β*4* nAChR antagonist SR 16584 into the IPn of nicotine-dependent mice precipitated physical signs of nicotine withdrawal (Zhao-Shea et al., 2013). Together, these findings suggest that *β*4* nAChRs and perhaps *α*5* and *α*2* nAChRs located in the mHb-IPn circuit regulate physical signs of nicotine withdrawal. However, the mHb-IPn circuit also regulates the increases in anxiety-related behaviors that occur during nicotine withdrawal, although *β*4*, *α*5*, and *α*2* nAChRs do not appear to be involved. This may instead reflect the contribution of *β*2* nAChR-expressing dopamine neurons in the VTA that express CRF and project to the IPn to modulate anxiety-related states (Grieder et al., 2014; Zhao-Shea et al., 2015) (see above).

In addition to IPn neurons, mHb neurons also show adaptive alterations in their activity in response to chronic nicotine exposure, which contribute to the development of nicotine dependence and expression of the aversive nicotine withdrawal syndrome (Gorlich et al., 2013). In slice recordings, mHb neurons show tonic trains of action potentials (Kim and Chang, 2005; Gorlich et al., 2013), which likely reflects “pacemaker” activity driven by the high concentrations of HCN4 channels expressed by these cells (Monteggia et al., 2000; Santoro et al., 2000; Gorlich et al., 2013; Oyrer et al., 2019). This pattern of tonic cellular activity contrasts with LHb neurons, which show much lower levels of baseline activity but a greater propensity to engage in burst firing (Kim and Chang, 2005). Infusion of an HCN antagonist into the mHb of nicotine-dependent mice precipitated physical signs of withdrawal and increased anxiety-related behavior (Gorlich et al., 2013). No change in the rate of tonic firing of mHb neurons was detected in mice after chronic nicotine treatment (Gorlich et al., 2013). However, the ability of nicotine to stimulate mHb neurons was markedly enhanced during nicotine withdrawal in a manner that depended on *α*3*β*4* nAChRs (Gorlich et al., 2013). Hence, modulatory actions of nicotine on mHb neurons could alleviate signs of withdrawal regulated by the mHb-IPn circuit and thereby serve as a source of motivation to seek and consume the drug during periods of abstinence, which contributes to relapse vulnerability. Overall, these findings support a major role for the mHb-IPn circuit in regulating behavioral abnormalities associated with nicotine withdrawal.

## VII. Summary

The diversity of nAChR subtypes and their dense expression in brain systems involved in reward, aversion, mood regulation, and cognition explain the complex actions of nicotine contained in tobacco products on these behavioral processes. By carefully titrating their nicotine intake to dynamically control the activity of nAChRs in these brain systems, tobacco users can powerfully modulate the motivational, mood, and cognitive processes that drive the initiation, establishment, and maintenance of regular tobacco use. Better understanding of the nAChR subtypes involved in the actions of nicotine in the brain and periphery and identification of the precise circuits, cells, and molecules through which nAChRs transduce responses to nicotine will reveal fundamental new insights into the mechanisms of tobacco dependence. Moreover, because nAChRs have also been implicated in dependence on other classes of abused drugs, including alcohol, opioids, and cocaine, and tobacco smoking is highly comorbid with other neuropsychiatric disorders, such as schizophrenia, improved understanding of the mechanisms of tobacco use disorder may provide key insights into other substance use and neuropsychiatric disorders. Ultimately, it is hoped that such information will catalyze the development of “next-generation” therapeutics to combat these disorders.

## Abbreviations

aVTA: anterior VTA
CB_1_: cannabinoid 1
CNS: central nervous system
CPA: conditioned place avoidance
CPP: conditioned place preference
CRF: corticotropin-releasing hormone
DH*β*E: dihydro-*β*-erythroidine
Ex-4: exendin-4
GluN1: glutamate ionotropic receptor NMDA subunit 1
GluN3A: glutamate ionotropic receptor NMDA subunit 3A
GLP-1: glucagon-like peptide 1
GPCR: G protein–coupled receptor
HCN: hyperpolarization-activated cyclic nucleotide-gated channel
ICSS: intracranial self-stimulation
IPn: interpeduncular nucleus
LDTg: laterodorsal tegmental nucleus
LHb: lateral habenula
18-MC: 18-methoxycoronaridine
mHb: medial habenula
MLA: methyllycaconitine
mPFC: medial prefrontal cortex
NAc: nucleus accumbens
nAChR: nicotinic acetylcholine receptor
NMDA: *N*-methyl-d-aspartic acid
nTS: nucleus tractus solitarius/nucleus of the solitary tract
PDE2A: phosphodiesterase 2A
PPTg: pedunculopontine tegmental nucleus
pVTA: posterior VTA
RMTg: rostromedial tegmental nucleus
shRNA: short interfering ribonucleic acid
TCF7L2: transcription factor 7–like 2
VH: ventral hippocampus
VTA: ventral tegmental area
VTA^MED^: medial VTA
VTA^LAT^: lateral VTA.

## References

[B3] Ables JL Görlich A Antolin-Fontes B Wang C Lipford SM Riad MH Ren J Hu F Luo M Kenny PJ, (2017) Retrograde inhibition by a specific subset of interpeduncular α5 nicotinic neurons regulates nicotine preference. Proc Natl Acad Sci USA 114:13012–13017. 29158387 10.1073/pnas.1717506114 PMC5724287

[B4] Acquas E Carboni E Leone P Di Chiara G (1989) SCH 23390 blocks drug-conditioned place-preference and place-aversion: anhedonia (lack of reward) or apathy (lack of motivation) after dopamine-receptor blockade? Psychopharmacology (Berl) 99:151–155. 2572027 10.1007/BF00442800

[B5] Agetsuma M Aizawa H Aoki T, (2010) The habenula is crucial for experiencedependent modification of fear responses in zebrafish. Nat Neurosci 13:1354–1356. 20935642 10.1038/nn.2654

[B2] Ahijevych K Tepper BJ Graham MC Holloman C Watson WA (2015) Relationships of PROP taste phenotype, taste receptor genotype, and oral nicotine replacement use. Nicotine Tob Res 17:1149–1155. 25542917 10.1093/ntr/ntu281 PMC4542738

[B6] Ai J Taylor KM Lisko JG Tran H Watson CH Holman MR (2016) Menthol content in US marketed cigarettes. Nicotine Tob Res 18:1575–1580. 26259988 10.1093/ntr/ntv162 PMC4747842

[B7] Aizawa H Kobayashi M Tanaka S Fukai T Okamoto H (2012) Molecular characterization of the subnuclei in rat habenula. J Comp Neurol 520:4051–4066. 22700183 10.1002/cne.23167

[B8] Aizawa H Zhu M (2019) Toward an understanding of the habenula’s various roles in human depression. Psychiatry Clin Neurosci 73:607–612. 31131942 10.1111/pcn.12892

[B9] Akers AT Cooper SY Baumgard ZJ Casinelli GP Avelar AJ Henderson BJ (2020) Upregulation of nAChRs and changes in excitability on VTA dopamine and GABA neurons correlates to changes in nicotine-reward-related behavior. eNeuro 7:ENEURO.0189-20.2020. 10.1523/ENEURO.0189-20.2020 PMC7568605 32988984

[B11] Albuquerque EX Pereira EF Alkondon M Rogers SW (2009) Mammalian nicotinic acetylcholine receptors: from structure to function. Physiol Rev 89:73–120. 19126755 10.1152/physrev.00015.2008 PMC2713585

[B12] Albuquerque EX Pereira EF Castro NG Alkondon M Reinhardt S Schröder H Maelicke A (1995) Nicotinic receptor function in the mammalian central nervous system. Ann N Y Acad Sci 757:48–72. 7611705 10.1111/j.1749-6632.1995.tb17464.x

[B13] Alderson HL Latimer MP Winn P (2005) Involvement of the laterodorsal tegmental nucleus in the locomotor response to repeated nicotine administration. Neurosci Lett 380:335–339. 15862913 10.1016/j.neulet.2005.01.067

[B14] Alderson HL Latimer MP Winn P (2006) Intravenous self-administration of nicotine is altered by lesions of the posterior, but not anterior, pedunculopontine tegmental nucleus. Eur J Neurosci 23:2169–2175. 16630063 10.1111/j.1460-9568.2006.04737.x

[B15] Alderson HL Latimer MP Winn P (2008) A functional dissociation of the anterior and posterior pedunculopontine tegmental nucleus: excitotoxic lesions have differential effects on locomotion and the response to nicotine. Brain Struct Funct 213:247–253. 18266007 10.1007/s00429-008-0174-4 PMC2522332

[B16] Alkhlaif Y Bagdas D Jackson A Park AJ Damaj IM (2017) Assessment of nicotine withdrawal-induced changes in sucrose preference in mice. Pharmacol Biochem Behav 161:47–52. 28919072 10.1016/j.pbb.2017.08.013 PMC6408212

[B17] Alkondon M Albuquerque EX (2005) Nicotinic receptor subtypes in rat hippocampal slices are differentially sensitive to desensitization and early in vivo functional up-regulation by nicotine and to block by bupropion. J Pharmacol Exp Ther 313:740–750. 15647329 10.1124/jpet.104.081232

[B18] Amos CI Wu X Broderick P Gorlov IP Gu J Eisen T Dong Q Zhang Q Gu X Vijayakrishnan J, (2008) Genome-wide association scan of tag SNPs identifies a susceptibility locus for lung cancer at 15q25.1. Nat Genet 40:616–622. 18385676 10.1038/ng.109 PMC2713680

[B19] Andres KH von Düring M Veh RW (1999) Subnuclear organization of the rat habenular complexes. J Comp Neurol 407:130–150. 10213193 10.1002/(sici)1096-9861(19990428)407:1<130::aid-cne10>3.0.co;2-8

[B20] Antolin-Fontes B Ables JL Görlich A Ibañez-Tallon I (2015) The habenulo-interpeduncular pathway in nicotine aversion and withdrawal. Neuropharmacology 96 (Pt B):213–222. 25476971 10.1016/j.neuropharm.2014.11.019 PMC4452453

[B21] Antolin-Fontes B Li K Ables JL Riad MH Görlich A Williams M Wang C Lipford SM Dao M Liu J, (2020) The habenular G-protein-coupled receptor 151 regulates synaptic plasticity and nicotine intake. Proc Natl Acad Sci USA 117:5502–5509. 32098843 10.1073/pnas.1916132117 PMC7071848

[B22] Appleyard SM Marks D Kobayashi K Okano H Low MJ Andresen MC (2007) Visceral afferents directly activate catecholamine neurons in the solitary tract nucleus. J Neurosci 27:13292–13302. 18045923 10.1523/JNEUROSCI.3502-07.2007 PMC6673415

[B23] Arvin MC Jin XT Yan Y Wang Y Ramsey MD Kim VJ Beckley NA Henry BA Drenan RM (2019) Chronic nicotine exposure alters the neurophysiology of habenulo-interpeduncular circuitry. J Neurosci 39:4268–4281. 30867261 10.1523/JNEUROSCI.2816-18.2019 PMC6538858

[B24] Avale ME Faure P Pons S Robledo P Deltheil T David DJ Gardier AM Maldonado R Granon S Changeux JP, (2008) Interplay of beta2* nicotinic receptors and dopamine pathways in the control of spontaneous locomotion. Proc Natl Acad Sci USA 105:15991–15996. 18832468 10.1073/pnas.0807635105 PMC2559801

[B25] Avelar AJ Akers AT Baumgard ZJ Cooper SY Casinelli GP Henderson BJ (2019) Why flavored vape products may be attractive: green apple tobacco flavor elicits reward-related behavior, upregulates nAChRs on VTA dopamine neurons, and alters midbrain dopamine and GABA neuron function. Neuropharmacology 158:107729. 31369741 10.1016/j.neuropharm.2019.107729 PMC6751572

[B26] Azam L Maskos U Changeux JP Dowell CD Christensen S De Biasi M McIntosh JM (2010) α-Conotoxin BuIA[T5A;P6O]: a novel ligand that discriminates between α6ß4 and α6ß2 nicotinic acetylcholine receptors and blocks nicotine-stimulated norepinephrine release. FASEB J 24:5113–5123. 20739611 10.1096/fj.10-166272 PMC3229426

[B27] Azam L Winzer-Serhan UH Chen Y Leslie FM (2002) Expression of neuronal nicotinic acetylcholine receptor subunit mRNAs within midbrain dopamine neurons. J Comp Neurol 444:260–274. 11840479 10.1002/cne.10138

[B28] Azhderian EM Hefner D Lin CH Kaczmarek LK Forscher P (1994) Cyclic AMP modulates fast axonal transport in Aplysia bag cell neurons by increasing the probability of single organelle movement. Neuron 12:1223–1233. 7516686 10.1016/0896-6273(94)90439-1

[B29] Bagdas D Diester CM Riley J Carper M Alkhlaif Y AlOmari D Alayoubi H Poklis JL Damaj MI (2019) Assessing nicotine dependence using an oral nicotine free-choice paradigm in mice. Neuropharmacology 157:107669. 31220484 10.1016/j.neuropharm.2019.107669 PMC6697382

[B30] Barlow RB McLeod LJ (1969) Some studies on cytisine and its methylated derivatives. Br J Pharmacol 35:161–174. 4387392 10.1111/j.1476-5381.1969.tb07977.x PMC1703083

[B31] Barrot M Sesack SR Georges F Pistis M Hong S Jhou TC (2012) Braking dopamine systems: a new GABA master structure for mesolimbic and nigrostriatal functions. J Neurosci 32:14094–14101. 23055478 10.1523/JNEUROSCI.3370-12.2012 PMC3513755

[B32] Bassareo V Tanda G Di Chiara G (1995) Increase of extracellular dopamine in the medial prefrontal cortex during spontaneous and naloxone-precipitated opiate abstinence. Psychopharmacology (Berl) 122:202–205. 8848538 10.1007/BF02246097

[B33] Bell RL Eiler BJ 2nd Cook JB Rahman S (2009) Nicotinic receptor ligands reduce ethanol intake by high alcohol-drinking HAD-2 rats. Alcohol 43:581–592. 20004336 10.1016/j.alcohol.2009.09.027 PMC2813038

[B34] Bello NT Moran TH (2008) GLP-1 agonists and satiety. Immunol Endocr Metab Agents Med Chem 8:311–316.

[B35] Benowitz NL (2009) Pharmacology of nicotine: addiction, smoking-induced disease, and therapeutics. Annu Rev Pharmacol Toxicol 49:57–71. 18834313 10.1146/annurev.pharmtox.48.113006.094742 PMC2946180

[B36] Berrendero F Kieffer BL Maldonado R (2002) Attenuation of nicotine-induced antinociception, rewarding effects, and dependence in mu-opioid receptor knock-out mice. J Neurosci 22:10935–10940. 12486188 10.1523/JNEUROSCI.22-24-10935.2002 PMC6758457

[B37] Berrendero F Mendizábal V Robledo P Galeote L Bilkei-Gorzo A Zimmer A Maldonado R (2005) Nicotine-induced antinociception, rewarding effects, and physical dependence are decreased in mice lacking the preproenkephalin gene. J Neurosci 25:1103–1112. 15689546 10.1523/JNEUROSCI.3008-04.2005 PMC6725961

[B38] Berrettini W Yuan X Tozzi F Song K Francks C Chilcoat H Waterworth D Muglia P Mooser V (2008) Alpha-5/alpha-3 nicotinic receptor subunit alleles increase risk for heavy smoking. Mol Psychiatry 13:368–373. 18227835 10.1038/sj.mp.4002154 PMC2507863

[B39] Besson M David V Suarez S Cormier A Cazala P Changeux JP Granon S (2006) Genetic dissociation of two behaviors associated with nicotine addiction: beta-2 containing nicotinic receptors are involved in nicotine reinforcement but not in withdrawal syndrome. Psychopharmacology (Berl) 187:189–199. 16752141 10.1007/s00213-006-0418-z

[B40] Bierut LJ Madden PA Breslau N Johnson EO Hatsukami D Pomerleau OF Swan GE Rutter J Bertelsen S Fox L, (2007) Novel genes identified in a high-density genome wide association study for nicotine dependence. Hum Mol Genet 16:24–35. 17158188 10.1093/hmg/ddl441 PMC2278047

[B41] Bierut LJ Stitzel JA Wang JC Hinrichs AL Grucza RA Xuei X Saccone NL Saccone SF Bertelsen S Fox L, (2008) Variants in nicotinic receptors and risk for nicotine dependence. Am J Psychiatry 165:1163–1171. 18519524 10.1176/appi.ajp.2008.07111711 PMC2574742

[B42] Blaha CD Allen LF Das S Inglis WL Latimer MP Vincent SR Winn P (1996) Modulation of dopamine efflux in the nucleus accumbens after cholinergic stimulation of the ventral tegmental area in intact, pedunculopontine tegmental nucleus-lesioned, and laterodorsal tegmental nucleus-lesioned rats. J Neurosci 16:714–722. 8551354 10.1523/JNEUROSCI.16-02-00714.1996 PMC6578651

[B43] Bordia T Hrachova M Chin M McIntosh JM Quik M (2012) Varenicline is a potent partial agonist at α6β2* nicotinic acetylcholine receptors in rat and monkey striatum. J Pharmacol Exp Ther 342:327–334. 22550286 10.1124/jpet.112.194852 PMC3400806

[B44] Bozarth MA (1994) Physical dependence produced by central morphine infusions: an anatomical mapping study. Neurosci Biobehav Rev 18:373–383. 7984355 10.1016/0149-7634(94)90050-7

[B45] Breese CR Marks MJ Logel J Adams CE Sullivan B Collins AC Leonard S (1997) Effect of smoking history on [3H]nicotine binding in human postmortem brain. J Pharmacol Exp Ther 282:7–13. 9223534

[B46] Brimblecombe KR Threlfell S Dautan D Kosillo P Mena-Segovia J Cragg SJ (2018) Targeted activation of cholinergic interneurons accounts for the modulation of dopamine by striatal nicotinic receptors. eNeuro 5:5. 10.1523/ENEURO.0397-17.2018 PMC6220583 30406189

[B47] Broadbent S Groot-Kormelink PJ Krashia PA Harkness PC Millar NS Beato M Sivilotti LG (2006) Incorporation of the beta3 subunit has a dominant-negative effect on the function of recombinant central-type neuronal nicotinic receptors. Mol Pharmacol 70:1350–1357. 16822928 10.1124/mol.106.026682

[B48] Brody AL Mandelkern MA Olmstead RE Allen-Martinez Z Scheibal D Abrams AL Costello MR Farahi J Saxena S Monterosso J, (2009) Ventral striatal dopamine release in response to smoking a regular vs a denicotinized cigarette. Neuropsychopharmacology 34:282–289. 18563061 10.1038/npp.2008.87 PMC2777990

[B49] Brody AL Mandelkern MA Olmstead RE Scheibal D Hahn E Shiraga S Zamora-Paja E Farahi J Saxena S London ED, (2006) Gene variants of brain dopamine pathways and smoking-induced dopamine release in the ventral caudate/nucleus accumbens. Arch Gen Psychiatry 63:808–816. 16818870 10.1001/archpsyc.63.7.808 PMC2873693

[B50] Brody AL Mukhin AG Mamoun MS Luu T Neary M Liang L Shieh J Sugar CA Rose JE Mandelkern MA (2014) Brain nicotinic acetylcholine receptor availability and response to smoking cessation treatment: a randomized trial. JAMA Psychiatry 71:797–805. 24850280 10.1001/jamapsychiatry.2014.138 PMC4634637

[B51] Brody AL Olmstead RE London ED Farahi J Meyer JH Grossman P Lee GS Huang J Hahn EL Mandelkern MA (2004) Smoking-induced ventral striatum dopamine release. Am J Psychiatry 161:1211–1218. 15229053 10.1176/appi.ajp.161.7.1211

[B52] Bromberg-Martin ES Matsumoto M Hikosaka O (2010) Dopamine in motivational control: rewarding, aversive, and alerting. Neuron 68:815–834. 21144997 10.1016/j.neuron.2010.11.022 PMC3032992

[B53] Broms J Antolin-Fontes B Tingström A Ibañez-Tallon I (2015) Conserved expression of the GPR151 receptor in habenular axonal projections of vertebrates. J Comp Neurol 523:359–380. 25116430 10.1002/cne.23664 PMC4270839

[B54] Broms J Grahm M Haugegaard L Blom T Meletis K Tingström A (2017) Monosynaptic retrograde tracing of neurons expressing the G-protein coupled receptor Gpr151 in the mouse brain. J Comp Neurol 525:3227–3250. 28657115 10.1002/cne.24273 PMC5601234

[B55] Brown DA Docherty RJ Halliwell JV (1983) Chemical transmission in the rat interpeduncular nucleus in vitro. J Physiol 341:655–670. 6137562 10.1113/jphysiol.1983.sp014831 PMC1195356

[B56] Bruijnzeel AW Markou A (2004) Adaptations in cholinergic transmission in the ventral tegmental area associated with the affective signs of nicotine withdrawal in rats. Neuropharmacology 47:572–579. 15380374 10.1016/j.neuropharm.2004.05.005

[B57] Brunzell DH (2012) Preclinical evidence that activation of mesolimbic alpha 6 subunit containing nicotinic acetylcholine receptors supports nicotine addiction phenotype. Nicotine Tob Res 14:1258–1269. 22492084 10.1093/ntr/nts089 PMC3482009

[B58] Brunzell DH Boschen KE Hendrick ES Beardsley PM McIntosh JM (2010) Alpha-conotoxin MII-sensitive nicotinic acetylcholine receptors in the nucleus accumbens shell regulate progressive ratio responding maintained by nicotine. Neuropsychopharmacology 35:665–673. 19890263 10.1038/npp.2009.171 PMC2821821

[B59] Bryant DL Free RB Thomasy SM Lapinsky DJ Ismail KA McKay SB Bergmeier SC McKay DB (2002) Structure-activity studies with ring E analogues of methyllycaconitine on bovine adrenal alpha3beta4* nicotinic receptors. Neurosci Res 42:57–63. 11814609 10.1016/s0168-0102(01)00304-2

[B60] Bueno D Lima LB Souza R Gonçalves L Leite F Souza S Furigo IC Donato J Jr Metzger M (2019) Connections of the laterodorsal tegmental nucleus with the habenular-interpeduncular-raphe system. J Comp Neurol 527:3046–3072. 31199515 10.1002/cne.24729

[B61] Cachope R Mateo Y Mathur BN Irving J Wang HL Morales M Lovinger DM Cheer JF (2012) Selective activation of cholinergic interneurons enhances accumbal phasic dopamine release: setting the tone for reward processing. Cell Rep 2:33–41. 22840394 10.1016/j.celrep.2012.05.011 PMC3408582

[B62] Caffery PM Krishnaswamy A Sanders T Liu J Hartlaub H Klysik J Cooper E Hawrot E (2009) Engineering neuronal nicotinic acetylcholine receptors with functional sensitivity to alpha-bungarotoxin: a novel alpha3-knock-in mouse. Eur J Neurosci 30:2064–2076. 20128845 10.1111/j.1460-9568.2009.07016.x PMC2818262

[B63] Cahir E Pillidge K Drago J Lawrence AJ (2011) The necessity of α4* nicotinic receptors in nicotine-driven behaviors: dissociation between reinforcing and motor effects of nicotine. Neuropsychopharmacology 36:1505–1517. 21430644 10.1038/npp.2011.35 PMC3096818

[B64] Cain WS(1980) Sensory attributes of cigarette smoking. In: A Safe Cigarette? (G. B. Gori F. G. Bock eds.), Vol. 3. Cold Spring Harbor Laboratory, New York, Banbury Center.

[B65] Campling BG Kuryatov A Lindstrom J (2013) Acute activation, desensitization and smoldering activation of human acetylcholine receptors. PLoS One 8:e79653. 24244538 10.1371/journal.pone.0079653 PMC3828267

[B66] Carboni E Bortone L Giua C Di Chiara G (2000a) Dissociation of physical abstinence signs from changes in extracellular dopamine in the nucleus accumbens and in the prefrontal cortex of nicotine dependent rats. Drug Alcohol Depend 58:93–102. 10669059 10.1016/s0376-8716(99)00064-2

[B67] Carboni E Silvagni A Rolando MT Di Chiara G (2000b) Stimulation of in vivo dopamine transmission in the bed nucleus of stria terminalis by reinforcing drugs. J Neurosci 20:RC102. 11027253 10.1523/JNEUROSCI.20-20-j0002.2000 PMC6772858

[B68] Champtiaux N Gotti C Cordero-Erausquin M David DJ Przybylski C Léna C Clementi F Moretti M Rossi FM Le Novère N, (2003) Subunit composition of functional nicotinic receptors in dopaminergic neurons investigated with knock-out mice. J Neurosci 23:7820–7829. 12944511 10.1523/JNEUROSCI.23-21-07820.2003 PMC6740613

[B69] Champtiaux N Han ZY Bessis A Rossi FM Zoli M Marubio L McIntosh JM Changeux JP (2002) Distribution and pharmacology of alpha 6-containing nicotinic acetylcholine receptors analyzed with mutant mice. J Neurosci 22:1208–1217. 11850448 10.1523/JNEUROSCI.22-04-01208.2002 PMC6757563

[B70] Chang FC Scott TR (1984) Conditioned taste aversions modify neural responses in the rat nucleus tractus solitarius. J Neurosci 4:1850–1862. 6737042 10.1523/JNEUROSCI.04-07-01850.1984 PMC6564891

[B71] Changeux JP (1979) The acetylcholine receptor: an “allosteric” membrane protein. Harvey Lect 75:85–254. 400609

[B72] Changeux JP Devillers-Thiéry A Chemouilli P (1984) Acetylcholine receptor: an allosteric protein. Science 225:1335–1345. 6382611 10.1126/science.6382611

[B73] Changeux JP Taly A (2008) Nicotinic receptors, allosteric proteins and medicine. Trends Mol Med 14:93–102. 18262468 10.1016/j.molmed.2008.01.001

[B74] Charpantier E Barnéoud P Moser P Besnard F Sgard F (1998) Nicotinic acetylcholine subunit mRNA expression in dopaminergic neurons of the rat substantia nigra and ventral tegmental area. Neuroreport 9:3097–3101. 9804323 10.1097/00001756-199809140-00033

[B75] Chatterjee S Santos N Holgate J Haass-Koffler CL Hopf FW Kharazia V Lester H Bonci A Bartlett SE (2013) The α5 subunit regulates the expression and function of α4*-containing neuronal nicotinic acetylcholine receptors in the ventral-tegmental area. PLoS One 8:e68300. 23869214 10.1371/journal.pone.0068300 PMC3712017

[B76] Chen C Regehr WG (1997) The mechanism of cAMP-mediated enhancement at a cerebellar synapse. J Neurosci 17:8687–8694. 9348337 10.1523/JNEUROSCI.17-22-08687.1997 PMC6573078

[B77] Chen L Lodge DJ (2013) The lateral mesopontine tegmentum regulates both tonic and phasic activity of VTA dopamine neurons. J Neurophysiol 110:2287–2294. 24004527 10.1152/jn.00307.2013 PMC3841868

[B78] Chen T Dong YX Li YQ (2003) Fos expression in serotonergic neurons in the rat brainstem following noxious stimuli: an immunohistochemical double-labelling study. J Anat 203:579–588. 14686693 10.1046/j.1469-7580.2003.00242.x PMC1571201

[B79] Chiang YT Ip W Jin T (2012) The role of the Wnt signaling pathway in incretin hormone production and function. Front Physiol 3:273. 22934027 10.3389/fphys.2012.00273 PMC3429047

[B80] Cimino M Marini P Fornasari D Cattabeni F Clementi F (1992) Distribution of nicotinic receptors in cynomolgus monkey brain and ganglia: localization of alpha 3 subunit mRNA, alpha-bungarotoxin and nicotine binding sites. Neuroscience 51:77–86. 1465189 10.1016/0306-4522(92)90472-e

[B81] Clarke PB Fu DS Jakubovic A Fibiger HC (1988) Evidence that mesolimbic dopaminergic activation underlies the locomotor stimulant action of nicotine in rats. J Pharmacol Exp Ther 246:701–708. 3136244

[B82] Clarke PB Hamill GS Nadi NS Jacobowitz DM Pert A (1986) 3H-nicotine- and 125I-alpha-bungarotoxin-labeled nicotinic receptors in the interpeduncular nucleus of rats. II. Effects of habenular deafferentation. J Comp Neurol 251:407–413. 3771837 10.1002/cne.902510311

[B83] Clarke PB Kumar R (1983a) Characterization of the locomotor stimulant action of nicotine in tolerant rats. Br J Pharmacol 80:587–594. 6640208 10.1111/j.1476-5381.1983.tb10733.x PMC2044999

[B84] Clarke PB Kumar R (1983b) The effects of nicotine on locomotor activity in non-tolerant and tolerant rats. Br J Pharmacol 78:329–337. 6131718 10.1111/j.1476-5381.1983.tb09398.x PMC2044704

[B85] Clarke PB Pert A (1985) Autoradiographic evidence for nicotine receptors on nigrostriatal and mesolimbic dopaminergic neurons. Brain Res 348:355–358. 4075093 10.1016/0006-8993(85)90456-1

[B86] Clarke PB Schwartz RD Paul SM Pert CB Pert A (1985) Nicotinic binding in rat brain: autoradiographic comparison of [3H]acetylcholine, [3H]nicotine, and [125I]-alpha-bungarotoxin. J Neurosci 5:1307–1315. 3998824 10.1523/JNEUROSCI.05-05-01307.1985 PMC6565049

[B87] Coe JW Brooks PR Vetelino MG Wirtz MC Arnold EP Huang J Sands SB Davis TI Lebel LA Fox CB, (2005) Varenicline: an alpha4beta2 nicotinic receptor partial agonist for smoking cessation. J Med Chem 48:3474–3477. 15887955 10.1021/jm050069n

[B88] Cohen C Bergis OE Galli F Lochead AW Jegham S Biton B Leonardon J Avenet P Sgard F Besnard F, (2003) SSR591813, a novel selective and partial alpha4beta2 nicotinic receptor agonist with potential as an aid to smoking cessation. J Pharmacol Exp Ther 306:407–420. 12682217 10.1124/jpet.103.049262

[B89] Coimbra B Soares-Cunha C Vasconcelos NAP Domingues AV Borges S Sousa N Rodrigues AJ (2019) Role of laterodorsal tegmentum projections to nucleus accumbens in reward-related behaviors. Nat Commun 10:4138. 31515512 10.1038/s41467-019-11557-3 PMC6742663

[B90] Collaborators GBDT; GBD 2015 Tobacco Collaborators (2017) Smoking prevalence and attributable disease burden in 195 countries and territories, 1990-2015: a systematic analysis from the Global Burden of Disease Study 2015. Lancet 389:1885–1906. 28390697 10.1016/S0140-6736(17)30819-X PMC5439023

[B91] Connolly JG Gibb AJ Colquhoun D (1995) Heterogeneity of neuronal nicotinic acetylcholine receptors in thin slices of rat medial habenula. J Physiol 484:87–105. 7541465 10.1113/jphysiol.1995.sp020650 PMC1157924

[B92] Contestabile A Villani L Fasolo A Franzoni MF Gribaudo L Oktedalen O Fonnum F (1987) Topography of cholinergic and substance P pathways in the habenulo-interpeduncular system of the rat. An immunocytochemical and microchemical approach. Neuroscience 21:253–270. 2439945 10.1016/0306-4522(87)90337-x

[B93] Correa VL Flores RJ Carcoba LM Arreguin MC O’Dell LE (2019) Sex differences in cholinergic systems in the interpeduncular nucleus following nicotine exposure and withdrawal. Neuropharmacology 158:107714. 31325431 10.1016/j.neuropharm.2019.107714 PMC6752203

[B94] Corrigall WA Coen KM (1989) Nicotine maintains robust self-administration in rats on a limited-access schedule. Psychopharmacology (Berl) 99:473–478. 2594913 10.1007/BF00589894

[B95] Corrigall WA Coen KM (1991) Selective dopamine antagonists reduce nicotine self-administration. Psychopharmacology (Berl) 104:171–176. 1876661 10.1007/BF02244174

[B96] Corrigall WA Coen KM Zhang J Adamson L (2002) Pharmacological manipulations of the pedunculopontine tegmental nucleus in the rat reduce self-administration of both nicotine and cocaine. Psychopharmacology (Berl) 160:198–205. 11875638 10.1007/s00213-001-0965-2

[B97] Corrigall WA Franklin KB Coen KM Clarke PB (1992) The mesolimbic dopaminergic system is implicated in the reinforcing effects of nicotine. Psychopharmacology (Berl) 107:285–289. 1615127 10.1007/BF02245149

[B98] Cover, K. K. Gyawali, U. Kerkhoff, W. G., (2019) Activation of the rostral intralaminar thalamus drives reinforcement through striatal dopamine release. Cell Rep 26, 13891398 e1383. 10.1016/j.celrep.2019.01.044 PMC6402336 30726725

[B99] Cragg SJ (2003) Variable dopamine release probability and short-term plasticity between functional domains of the primate striatum. J Neurosci 23:4378–4385. 12764127 10.1523/JNEUROSCI.23-10-04378.2003 PMC6741072

[B100] Cui C Booker TK Allen RS Grady SR Whiteaker P Marks MJ Salminen O Tritto T Butt CM Allen WR, (2003) The beta3 nicotinic receptor subunit: a component of alpha-conotoxin MII-binding nicotinic acetylcholine receptors that modulate dopamine release and related behaviors. J Neurosci 23:11045–11053. 14657161 10.1523/JNEUROSCI.23-35-11045.2003 PMC6741047

[B101] D’Silva J Cohn AM Johnson AL Villanti AC (2018) Differences in subjective experiences to first use of menthol and nonmenthol cigarettes in a national sample of young adult cigarette smokers. Nicotine Tob Res 20:1062–1068. 29059351 10.1093/ntr/ntx181 PMC6093322

[B102] Dahl M Erickson RP Simon SA (1997) Neural responses to bitter compounds in rats. Brain Res 756:22–34. 9187310 10.1016/s0006-8993(97)00131-5

[B103] Dao DQ Perez EE Teng Y Dani JA De Biasi M (2014) Nicotine enhances excitability of medial habenular neurons via facilitation of neurokinin signaling. J Neurosci 34:4273–4284. 24647947 10.1523/JNEUROSCI.2736-13.2014 PMC3960468

[B104] Dash B Bhakta M Chang Y Lukas RJ (2012) Modulation of recombinant, α2*, α3* or α4*-nicotinic acetylcholine receptor (nAChR) function by nAChR β3 subunits. J Neurochem 121:349–361. 22309577 10.1111/j.1471-4159.2012.07685.x PMC3569009

[B105] Dash B Li MD (2014) Two rare variations, D478N and D478E, that occur at the same amino acid residue in nicotinic acetylcholine receptor (nAChR) α2 subunit influence nAChR function. Neuropharmacology 85:471–481. 24950454 10.1016/j.neuropharm.2014.05.014 PMC4135378

[B106] Dautan D Huerta-Ocampo I Witten IB Deisseroth K Bolam JP Gerdjikov T Mena-Segovia J (2014) A major external source of cholinergic innervation of the striatum and nucleus accumbens originates in the brainstem. J Neurosci 34:4509–4518. 24671996 10.1523/JNEUROSCI.5071-13.2014 PMC3965779

[B107] Dautan D Souza AS Huerta-Ocampo I Valencia M Assous M Witten IB Deisseroth K Tepper JM Bolam JP Gerdjikov TV, (2016) Segregated cholinergic transmission modulates dopamine neurons integrated in distinct functional circuits. Nat Neurosci 19:1025–1033. 27348215 10.1038/nn.4335 PMC5086413

[B108] David V Besson M Changeux JP Granon S Cazala P (2006) Reinforcing effects of nicotine microinjections into the ventral tegmental area of mice: dependence on cholinergic nicotinic and dopaminergic D1 receptors. Neuropharmacology 50:1030–1040. 16580026 10.1016/j.neuropharm.2006.02.003

[B109] David V Segu L Buhot MC Ichaye M Cazala P (2004) Rewarding effects elicited by cocaine microinjections into the ventral tegmental area of C57BL/6 mice: involvement of dopamine D1 and serotonin1B receptors. Psychopharmacology (Berl) 174:367–375. 15024548 10.1007/s00213-003-1767-5

[B110] Dawe S Gerada C Russell MA Gray JA (1995) Nicotine intake in smokers increases following a single dose of haloperidol. Psychopharmacology (Berl) 117:110–115. 7724695 10.1007/BF02245105

[B111] De Biasi M Dani JA (2011) Reward, addiction, withdrawal to nicotine. Annu Rev Neurosci 34:105–130. 21438686 10.1146/annurev-neuro-061010-113734 PMC3137256

[B112] De Biasi M Salas R (2008) Influence of neuronal nicotinic receptors over nicotine addiction and withdrawal. Exp Biol Med (Maywood) 233:917–929. 18480414 10.3181/0712-MR-355

[B584] de Wit H Phillips TJ (2012) Do initial responses to drugs predict future use or abuse? Neurosci Biobehav Rev 36:1565–1576. 22542906 10.1016/j.neubiorev.2012.04.005 PMC3372699

[B113] DeGroot SR Zhao-Shea R Chung L Klenowski PM Sun F Molas S Gardner PD Li Y Tapper AR (2020) Midbrain dopamine controls anxiety-like behavior by engaging unique interpeduncular nucleus microcircuitry. Biol Psychiatry 88:855–866. 32800629 10.1016/j.biopsych.2020.06.018 PMC8043246

[B114] Dehkordi O Rose JE Asadi S Manaye KF Millis RM Jayam-Trouth A (2015) Neuroanatomical circuitry mediating the sensory impact of nicotine in the central nervous system. J Neurosci Res 93:230–243. 25223294 10.1002/jnr.23477 PMC4270827

[B115] Dehkordi O Rose JE Millis RM Dalivand MM Johnson SM (2018) GABAergic neurons as putative neurochemical substrate mediating aversive effects of nicotine. J Alcohol Drug Depend 6:6. 10.4172/2329-6488.1000312 PMC6042868 30009210

[B116] Demontis D Rajagopal VM Thorgeirsson TE Als TD Grove J Leppälä K Gudbjartsson DF Pallesen J Hjorthøj C Reginsson GW, (2019) Genome-wide association study implicates CHRNA2 in cannabis use disorder. Nat Neurosci 22:1066–1074. 31209380 10.1038/s41593-019-0416-1 PMC7596896

[B117] Deneris ES Connolly J Rogers SW Duvoisin R (1991) Pharmacological and functional diversity of neuronal nicotinic acetylcholine receptors. Trends Pharmacol Sci 12:34–40. 2006540 10.1016/0165-6147(91)90486-c

[B118] DeNoble VJ Mele PC (2006) Intravenous nicotine self-administration in rats: effects of mecamylamine, hexamethonium and naloxone. Psychopharmacology (Berl) 184:266–272. 16088413 10.1007/s00213-005-0054-z

[B119] Di Chiara G (2000) Role of dopamine in the behavioural actions of nicotine related to addiction. Eur J Pharmacol 393:295–314. 10771025 10.1016/s0014-2999(00)00122-9

[B120] Di Chiara G Imperato A (1988) Drugs abused by humans preferentially increase synaptic dopamine concentrations in the mesolimbic system of freely moving rats. Proc Natl Acad Sci USA 85:5274–5278. 2899326 10.1073/pnas.85.14.5274 PMC281732

[B121] Doherty K Kinnunen T Militello FS Garvey AJ (1995) Urges to smoke during the first month of abstinence: relationship to relapse and predictors. Psychopharmacology (Berl) 119:171–178. 7659764 10.1007/BF02246158

[B122] Doll R Peto R Boreham J Sutherland I (2004) Mortality in relation to smoking: 50 years’ observations on male British doctors. BMJ 328:1519. 15213107 10.1136/bmj.38142.554479.AE PMC437139

[B123] Doll R Peto R Wheatley K Gray R Sutherland I (1994) Mortality in relation to smoking: 40 years’ observations on male British doctors. BMJ 309:901–911. 7755693 10.1136/bmj.309.6959.901 PMC2541142

[B124] Donovick PJ Burright RG Zuromski E (1970) Localization of quinine aversion within the septum, habenula, and interpeduncular nucleus of the rat. J Comp Physiol Psychol 71:376–383. 5480869 10.1037/h0029114

[B125] Drenan RM Grady SR Steele AD McKinney S Patzlaff NE McIntosh JM Marks MJ Miwa JM Lester HA (2010) Cholinergic modulation of locomotion and striatal dopamine release is mediated by alpha6alpha4* nicotinic acetylcholine receptors. J Neurosci 30:9877–9889. 20660270 10.1523/JNEUROSCI.2056-10.2010 PMC3390922

[B126] Drenan RM Nashmi R Imoukhuede P Just H McKinney S Lester HA (2008) Subcellular trafficking, pentameric assembly, and subunit stoichiometry of neuronal nicotinic acetylcholine receptors containing fluorescently labeled alpha6 and beta3 subunits. Mol Pharmacol 73:27–41. 17932221 10.1124/mol.107.039180

[B127] Duncan A Heyer MP Ishikawa M Caligiuri SPB Liu XA Chen Z Micioni Di Bonaventura MV Elayouby KS Ables JL Howe WM, (2019) Habenular TCF7L2 links nicotine addiction to diabetes. Nature 574:372–377. 31619789 10.1038/s41586-019-1653-x PMC9851388

[B128] Durand-de Cuttoli R Mondoloni S Marti F Lemoine D Nguyen C Naudé J d’Izarny-Gargas T Pons S Maskos U Trauner D, (2018) Manipulating midbrain dopamine neurons and reward-related behaviors with light-controllable nicotinic acetylcholine receptors. eLife 7:7. 10.7554/eLife.37487 PMC6122951 30176987

[B129] Dwoskin LP Smith AM Wooters TE Zhang Z Crooks PA Bardo MT (2009) Nicotinic receptor-based therapeutics and candidates for smoking cessation. Biochem Pharmacol 78:732–743. 19523455 10.1016/j.bcp.2009.06.002 PMC4110684

[B130] Edwards FA Gibb AJ Colquhoun D (1992) ATP receptor-mediated synaptic currents in the central nervous system. Nature 359:144–147. 1381811 10.1038/359144a0

[B131] Elayouby KS Ishikawa M Dukes AJ Smith ACW Lu Q Fowler CD Kenny PJ (2021) α3* Nicotinic acetylcholine receptors in the habenula-interpeduncular nucleus circuit regulate nicotine intake. J Neurosci 41:1779–1787. 33380469 10.1523/JNEUROSCI.0127-19.2020 PMC8115890

[B132] Elgoyhen AB Johnson DS Boulter J Vetter DE Heinemann S (1994) Alpha 9: an acetylcholine receptor with novel pharmacological properties expressed in rat cochlear hair cells. Cell 79:705–715. 7954834 10.1016/0092-8674(94)90555-x

[B133] Elgoyhen AB Vetter DE Katz E Rothlin CV Heinemann SF Boulter J (2001) alpha10: a determinant of nicotinic cholinergic receptor function in mammalian vestibular and cochlear mechanosensory hair cells. Proc Natl Acad Sci USA 98:3501–3506. 11248107 10.1073/pnas.051622798 PMC30682

[B134] Engle SE Shih PY McIntosh JM Drenan RM (2013) α4α6β2* nicotinic acetylcholine receptor activation on ventral tegmental area dopamine neurons is sufficient to stimulate a depolarizing conductance and enhance surface AMPA receptor function. Mol Pharmacol 84:393–406. 23788655 10.1124/mol.113.087346 PMC3876818

[B135] Epping-Jordan MP Watkins SS Koob GF Markou A (1998) Dramatic decreases in brain reward function during nicotine withdrawal. Nature 393:76–79. 9590692 10.1038/30001

[B136] Espejo EF Serrano MI Caillé S Stinus L (2001) Behavioral expression of opiate withdrawal is altered after prefrontocortical dopamine depletion in rats: monoaminergic correlates. Neuropsychopharmacology 25:204–212. 11425504 10.1016/S0893-133X(01)00226-3

[B137] Esterlis I Hillmer AT Bois F Pittman B McGovern E O’Malley SS Picciotto MR Yang BZ Gelernter J Cosgrove KP (2016) CHRNA4 and ANKK1 polymorphisms influence smoking-induced nicotinic acetylcholine receptor upregulation. Nicotine Tob Res 18:1845–1852. 27611310 10.1093/ntr/ntw081 PMC4978979

[B138] Esterlis I Ranganathan M Bois F Pittman B Picciotto MR Shearer L Anticevic A Carlson J Niciu MJ Cosgrove KP, (2014) In vivo evidence for β2 nicotinic acetylcholine receptor subunit upregulation in smokers as compared with nonsmokers with schizophrenia. Biol Psychiatry 76:495–502. 24360979 10.1016/j.biopsych.2013.11.001 PMC4019710

[B139] Etter JF (2006) Cytisine for smoking cessation: a literature review and a meta-analysis. Arch Intern Med 166:1553–1559. 16908787 10.1001/archinte.166.15.1553

[B140] Exley R Clements MA Hartung H McIntosh JM Cragg SJ (2008) Alpha6-containing nicotinic acetylcholine receptors dominate the nicotine control of dopamine neurotransmission in nucleus accumbens. Neuropsychopharmacology 33:2158–2166. 18033235 10.1038/sj.npp.1301617

[B141] Exley R Cragg SJ (2008) Presynaptic nicotinic receptors: a dynamic and diverse cholinergic filter of striatal dopamine neurotransmission. Br J Pharmacol 153 (Suppl 1):S283–S297. 18037926 10.1038/sj.bjp.0707510 PMC2268048

[B142] Exley R Maubourguet N David V Eddine R Evrard A Pons S Marti F Threlfell S Cazala P McIntosh JM, (2011) Distinct contributions of nicotinic acetylcholine receptor subunit alpha4 and subunit alpha6 to the reinforcing effects of nicotine. Proc Natl Acad Sci USA 108:7577–7582. 21502501 10.1073/pnas.1103000108 PMC3088627

[B143] Exley R McIntosh JM Marks MJ Maskos U Cragg SJ (2012) Striatal α5 nicotinic receptor subunit regulates dopamine transmission in dorsal striatum. J Neurosci 32:2352–2356. 22396410 10.1523/JNEUROSCI.4985-11.2012 PMC3742968

[B144] Ezzati M Lopez AD (2003) Estimates of global mortality attributable to smoking in 2000. Lancet 362:847–852. 13678970 10.1016/S0140-6736(03)14338-3

[B145] Fagerström KO (1988) Efficacy of nicotine chewing gum: a review. Prog Clin Biol Res 261:109–128. 3289000

[B146] Fagerström KO Schneider NG Lunell E (1993) Effectiveness of nicotine patch and nicotine gum as individual versus combined treatments for tobacco withdrawal symptoms. Psychopharmacology (Berl) 111:271–277. 7870963 10.1007/BF02244941

[B147] Faget L Osakada F Duan J Ressler R Johnson AB Proudfoot JA Yoo JH Callaway EM Hnasko TS (2016) Afferent inputs to neurotransmitter-defined cell types in the ventral tegmental area. Cell Rep 15:2796–2808. 27292633 10.1016/j.celrep.2016.05.057 PMC4917450

[B148] Feyerabend C Higenbottam T Russell MA (1982) Nicotine concentrations in urine and saliva of smokers and non-smokers. Br Med J (Clin Res Ed) 284:1002–1004. 10.1136/bmj.284.6321.1002 PMC1497991 6802384

[B149] File SE Cheeta S Kenny PJ (2000) Neurobiological mechanisms by which nicotine mediates different types of anxiety. Eur J Pharmacol 393:231–236. 10771018 10.1016/s0014-2999(99)00889-4

[B150] File SE Kenny PJ Ouagazzal AM (1998) Bimodal modulation by nicotine of anxiety in the social interaction test: role of the dorsal hippocampus. Behav Neurosci 112:1423–1429. 9926824 10.1037//0735-7044.112.6.1423

[B151] Flannery JS Riedel MC Poudel R Laird AR Ross TJ Salmeron BJ Stein EA Sutherland MT (2019) Habenular and striatal activity during performance feedback are differentially linked with state-like and trait-like aspects of tobacco use disorder. Sci Adv 5:eaax2084. 31633021 10.1126/sciadv.aax2084 PMC6785263

[B152] Flores CM Rogers SW Pabreza LA Wolfe BB Kellar KJ (1992) A subtype of nicotinic cholinergic receptor in rat brain is composed of alpha 4 and beta 2 subunits and is up-regulated by chronic nicotine treatment. Mol Pharmacol 41:31–37. 1732720

[B153] Fonck C Cohen BN Nashmi R Whiteaker P Wagenaar DA Rodrigues-Pinguet N Deshpande P McKinney S Kwoh S Munoz J, (2005) Novel seizure phenotype and sleep disruptions in knock-in mice with hypersensitive alpha 4* nicotinic receptors. J Neurosci 25:11396–11411. 16339034 10.1523/JNEUROSCI.3597-05.2005 PMC6725918

[B154] Fonck C Nashmi R Salas R Zhou C Huang Q De Biasi M Lester RA Lester HA (2009) Demonstration of functional alpha4-containing nicotinic receptors in the medial habenula. Neuropharmacology 56:247–253. 18789953 10.1016/j.neuropharm.2008.08.021 PMC2645341

[B155] Forget B Scholze P Langa F Morel C Pons S Mondoloni S Besson M Durand-de Cuttoli R Hay A Tricoire L, (2018) A human polymorphism in CHRNA5 is linked to relapse to nicotine seeking in transgenic rats. Curr Biol 28:3244–3253.e7. 30293722 10.1016/j.cub.2018.08.044

[B156] Forsayeth JR Kobrin E (1997) Formation of oligomers containing the beta3 and beta4 subunits of the rat nicotinic receptor. J Neurosci 17:1531–1538. 9030613 10.1523/JNEUROSCI.17-05-01531.1997 PMC6573367

[B157] Forster GL Blaha CD (2000) Laterodorsal tegmental stimulation elicits dopamine efflux in the rat nucleus accumbens by activation of acetylcholine and glutamate receptors in the ventral tegmental area. Eur J Neurosci 12:3596–3604. 11029630 10.1046/j.1460-9568.2000.00250.x

[B158] Fowler CD Arends MA Kenny PJ (2008) Subtypes of nicotinic acetylcholine receptors in nicotine reward, dependence, and withdrawal: evidence from genetically modified mice. Behav Pharmacol 19:461–484. 18690103 10.1097/FBP.0b013e32830c360e PMC2669417

[B159] Fowler CD Kenny PJ (2011) Intravenous nicotine self-administration and cue-induced reinstatement in mice: effects of nicotine dose, rate of drug infusion and prior instrumental training. Neuropharmacology 61:687–698. 21640128 10.1016/j.neuropharm.2011.05.012 PMC3130070

[B160] Fowler, C. D. Kenny, P. J. (2014) Nicotine aversion: Neurobiological mechanisms and relevance to tobacco dependence vulnerability. Neuropharmacology 76 Pt B, 533-544. 24055497 10.1016/j.neuropharm.2013.09.008 PMC3858456

[B161] Fowler CD Lu Q Johnson PM Marks MJ Kenny PJ (2011) Habenular α5 nicotinic receptor subunit signalling controls nicotine intake. Nature 471:597–601. 21278726 10.1038/nature09797 PMC3079537

[B162] Fowler, C. D. Tuesta, L. Kenny, P. J. (2013) Role of alpha5* nicotinic acetylcholine receptors in the effects of acute and chronic nicotine treatment on brain reward function in mice. Psychopharmacology (Berl). 10.1007/s00213-013-3235-1 PMC3930613 23958943

[B163] Frahm S Antolin-Fontes B Görlich A Zander JF Ahnert-Hilger G Ibañez-Tallon I (2015) An essential role of acetylcholine-glutamate synergy at habenular synapses in nicotine dependence. eLife 4:e11396. 26623516 10.7554/eLife.11396 PMC4718731

[B164] Frahm S Slimak MA Ferrarese L Santos-Torres J Antolin-Fontes B Auer S Filkin S Pons S Fontaine JF Tsetlin V, (2011) Aversion to nicotine is regulated by the balanced activity of β4 and α5 nicotinic receptor subunits in the medial habenula. Neuron 70:522–535. 21555077 10.1016/j.neuron.2011.04.013

[B165] Fu Y Matta SG Gao W Brower VG Sharp BM (2000) Systemic nicotine stimulates dopamine release in nucleus accumbens: re-evaluation of the role of N-methyl-D-aspartate receptors in the ventral tegmental area. J Pharmacol Exp Ther 294:458–465. 10900219

[B166] Fu Y Matta SG Kane VB Sharp BM (2003) Norepinephrine release in amygdala of rats during chronic nicotine self-administration: an in vivo microdialysis study. Neuropharmacology 45:514–523. 12907312 10.1016/s0028-3908(03)00201-6

[B167] Fucile S Barabino B Palma E Grassi F Limatola C Mileo AM Alemà S Ballivet M Eusebi F (1997) Alpha 5 subunit forms functional alpha 3 beta 4 alpha 5 nAChRs in transfected human cells. Neuroreport 8:2433–2436. 9261804 10.1097/00001756-199707280-00005

[B168] Galeote L Berrendero F Bura SA Zimmer A Maldonado R (2009) Prodynorphin gene disruption increases the sensitivity to nicotine self-administration in mice. Int J Neuropsychopharmacol 12:615–625. 18937881 10.1017/S1461145708009450

[B169] Gallego X Cox RJ Laughlin JR Stitzel JA Ehringer MA (2013) Alternative CHRNB4 3′-UTRs mediate the allelic effects of SNP rs1948 on gene expression. PLoS One 8:e63699. 23691088 10.1371/journal.pone.0063699 PMC3653846

[B170] Garcia-Diaz DE Jimenez-Montufar LL Guevara-Aguilar R Wayner MJ Armstrong DL (1988) Olfactory and visceral projections to the nucleus of the solitary tract. Physiol Behav 44:619–624. 3237848 10.1016/0031-9384(88)90327-7

[B171] Geng F Liu JY Chen XW Zou WJ Wu JL Rodriguez G Peng C Tian J Lu GF (2019) ErbB4 receptors in the medial habenula regulate contextual fear memory. Pharmacology 103:68–75. 30513516 10.1159/000495064

[B172] Gerzanich V Wang F Kuryatov A Lindstrom J (1998) alpha 5 Subunit alters desensitization, pharmacology, Ca++ permeability and Ca++ modulation of human neuronal alpha 3 nicotinic receptors. J Pharmacol Exp Ther 286:311–320. 9655874

[B173] Giniatullin R Nistri A Yakel JL (2005) Desensitization of nicotinic ACh receptors: shaping cholinergic signaling. Trends Neurosci 28:371–378. 15979501 10.1016/j.tins.2005.04.009

[B174] Girod R Barazangi N McGehee D Role LW (2000) Facilitation of glutamatergic neurotransmission by presynaptic nicotinic acetylcholine receptors. Neuropharmacology 39:2715–2725. 11044742 10.1016/s0028-3908(00)00145-3

[B175] Glick SD Sell EM McCallum SE Maisonneuve IM (2011) Brain regions mediating α3β4 nicotinic antagonist effects of 18-MC on nicotine self-administration. Eur J Pharmacol 669:71–75. 21871879 10.1016/j.ejphar.2011.08.001 PMC3183297

[B177] Göke R Larsen PJ Mikkelsen JD Sheikh SP (1995) Distribution of GLP-1 binding sites in the rat brain: evidence that exendin-4 is a ligand of brain GLP-1 binding sites. Eur J Neurosci 7:2294–2300. 8563978 10.1111/j.1460-9568.1995.tb00650.x

[B178] Goldberg SR Spealman RD (1982) Maintenance and suppression of behavior by intravenous nicotine injections in squirrel monkeys. Fed Proc 41:216–220. 7060749

[B179] Goldberg SR Spealman RD (1983) Suppression of behavior by intravenous injections of nicotine or by electric shocks in squirrel monkeys: effects of chlordiazepoxide and mecamylamine. J Pharmacol Exp Ther 224:334–340. 6822959

[B180] Goldberg SR Spealman RD Goldberg DM (1981) Persistent behavior at high rates maintained by intravenous self-administration of nicotine. Science 214:573–575. 7291998 10.1126/science.7291998

[B181] Good CH Lupica CR (2009) Properties of distinct ventral tegmental area synapses activated via pedunculopontine or ventral tegmental area stimulation in vitro. J Physiol 587:1233–1247. 19188251 10.1113/jphysiol.2008.164194 PMC2674994

[B182] Görlich A Antolin-Fontes B Ables JL Frahm S Slimak MA Dougherty JD Ibañez-Tallon I (2013) Reexposure to nicotine during withdrawal increases the pacemaking activity of cholinergic habenular neurons. Proc Natl Acad Sci USA 110:17077–17082. 24082085 10.1073/pnas.1313103110 PMC3800986

[B183] Goto M Swanson LW Canteras NS (2001) Connections of the nucleus incertus. J Comp Neurol 438:86–122. 11503154 10.1002/cne.1303

[B184] Gotti C Clementi F Fornari A Gaimarri A Guiducci S Manfredi I Moretti M Pedrazzi P Pucci L Zoli M (2009) Structural and functional diversity of native brain neuronal nicotinic receptors. Biochem Pharmacol 78:703–711. 19481063 10.1016/j.bcp.2009.05.024

[B185] Gotti C Guiducci S Tedesco V Corbioli S Zanetti L Moretti M Zanardi A Rimondini R Mugnaini M Clementi F, (2010) Nicotinic acetylcholine receptors in the mesolimbic pathway: primary role of ventral tegmental area alpha6beta2* receptors in mediating systemic nicotine effects on dopamine release, locomotion, and reinforcement. J Neurosci 30:5311–5325. 20392953 10.1523/JNEUROSCI.5095-09.2010 PMC6632743

[B186] Gotti C Moretti M Clementi F Riganti L McIntosh JM Collins AC Marks MJ Whiteaker P (2005) Expression of nigrostriatal alpha 6-containing nicotinic acetylcholine receptors is selectively reduced, but not eliminated, by beta 3 subunit gene deletion. Mol Pharmacol 67:2007–2015. 15749993 10.1124/mol.105.011940

[B187] Gotti C Moretti M Gaimarri A Zanardi A Clementi F Zoli M (2007) Heterogeneity and complexity of native brain nicotinic receptors. Biochem Pharmacol 74:1102–1111. 17597586 10.1016/j.bcp.2007.05.023

[B188] Gotti C Moretti M Meinerz NM Clementi F Gaimarri A Collins AC Marks MJ (2008) Partial deletion of the nicotinic cholinergic receptor alpha 4 or beta 2 subunit genes changes the acetylcholine sensitivity of receptor-mediated 86Rb+ efflux in cortex and thalamus and alters relative expression of alpha 4 and beta 2 subunits. Mol Pharmacol 73:1796–1807. 18337473 10.1124/mol.108.045203

[B189] Gotti C Riganti L Vailati S Clementi F (2006a) Brain neuronal nicotinic receptors as new targets for drug discovery. Curr Pharm Des 12:407–428. 16472136 10.2174/138161206775474486

[B190] Gotti C Zoli M Clementi F (2006b) Brain nicotinic acetylcholine receptors: native subtypes and their relevance. Trends Pharmacol Sci 27:482–491. 16876883 10.1016/j.tips.2006.07.004

[B191] Goutier W Lowry JP McCreary AC O’Connor JJ (2016) Frequency-dependent modulation of dopamine release by nicotine and dopamine D1 receptor ligands: an in vitro fast cyclic voltammetry study in rat striatum. Neurochem Res 41:945–950. 26975318 10.1007/s11064-015-1786-8

[B192] Grady SR Meinerz NM Cao J Reynolds AM Picciotto MR Changeux JP McIntosh JM Marks MJ Collins AC (2001) Nicotinic agonists stimulate acetylcholine release from mouse interpeduncular nucleus: a function mediated by a different nAChR than dopamine release from striatum. J Neurochem 76:258–268. 11145999 10.1046/j.1471-4159.2001.00019.x

[B193] Grady SR Moretti M Zoli M Marks MJ Zanardi A Pucci L Clementi F Gotti C (2009) Rodent habenulo-interpeduncular pathway expresses a large variety of uncommon nAChR subtypes, but only the alpha3beta4* and alpha3beta3beta4* subtypes mediate acetylcholine release. J Neurosci 29:2272–2282. 19228980 10.1523/JNEUROSCI.5121-08.2009 PMC2680386

[B194] Grady SR Salminen O Laverty DC Whiteaker P McIntosh JM Collins AC Marks MJ (2007) The subtypes of nicotinic acetylcholine receptors on dopaminergic terminals of mouse striatum. Biochem Pharmacol 74:1235–1246. 17825262 10.1016/j.bcp.2007.07.032 PMC2735219

[B195] Grady SR Salminen O McIntosh JM Marks MJ Collins AC (2010) Mouse striatal dopamine nerve terminals express alpha4alpha5beta2 and two stoichiometric forms of alpha4beta2*-nicotinic acetylcholine receptors. J Mol Neurosci 40:91–95. 19693710 10.1007/s12031-009-9263-y PMC4386732

[B196] Grady SR Wageman CR Patzlaff NE Marks MJ (2012) Low concentrations of nicotine differentially desensitize nicotinic acetylcholine receptors that include α5 or α6 subunits and that mediate synaptosomal neurotransmitter release. Neuropharmacology 62:1935–1943. 22239849 10.1016/j.neuropharm.2011.12.026 PMC3278500

[B197] Gray MA Critchley HD (2007) Interoceptive basis to craving. Neuron 54:183–186. 17442239 10.1016/j.neuron.2007.03.024 PMC2259270

[B198] Grenhoff J Aston-Jones G Svensson TH (1986) Nicotinic effects on the firing pattern of midbrain dopamine neurons. Acta Physiol Scand 128:351–358. 3788613 10.1111/j.1748-1716.1986.tb07988.x

[B199] Grieder TE Besson M Maal-Bared G Pons S Maskos U van der Kooy D (2019) β2* nAChRs on VTA dopamine and GABA neurons separately mediate nicotine aversion and reward. Proc Natl Acad Sci USA 116:25968–25973. 31776253 10.1073/pnas.1908724116 PMC6925992

[B200] Grieder TE George O Tan H George SR Le Foll B Laviolette SR van der Kooy D (2012) Phasic D1 and tonic D2 dopamine receptor signaling double dissociate the motivational effects of acute nicotine and chronic nicotine withdrawal. Proc Natl Acad Sci USA 109:3101–3106. 22308372 10.1073/pnas.1114422109 PMC3286981

[B201] Grieder TE George O Yee M Bergamini MA Chwalek M Maal-Bared G Vargas-Perez H van der Kooy D (2017) Deletion of α5 nicotine receptor subunits abolishes nicotinic aversive motivational effects in a manner that phenocopies dopamine receptor antagonism. Eur J Neurosci 46:1673–1681. 28498560 10.1111/ejn.13605 PMC8162765

[B202] Grieder TE Herman MA Contet C Tan LA Vargas-Perez H Cohen A Chwalek M Maal-Bared G Freiling J Schlosburg JE, (2014) VTA CRF neurons mediate the aversive effects of nicotine withdrawal and promote intake escalation. Nat Neurosci 17:1751–1758. 25402857 10.1038/nn.3872 PMC4241147

[B203] Grillner P Svensson TH (2000) Nicotine-induced excitation of midbrain dopamine neurons in vitro involves ionotropic glutamate receptor activation. Synapse 38:1–9. 10941135 10.1002/1098-2396(200010)38:1<1::AID-SYN1>3.0.CO;2-A

[B204] Groenewegen HJ Steinbusch HW (1984) Serotonergic and non-serotonergic projections from the interpeduncular nucleus to the ventral hippocampus in the rat. Neurosci Lett 51:19–24. 6096770 10.1016/0304-3940(84)90256-8

[B205] Grucza RA Wang JC Stitzel JA Hinrichs AL Saccone SF Saccone NL Bucholz KK Cloninger CR Neuman RJ Budde JP, (2008) A risk allele for nicotine dependence in CHRNA5 is a protective allele for cocaine dependence. Biol Psychiatry 64:922–929. 18519132 10.1016/j.biopsych.2008.04.018 PMC2582594

[B206] Gu S Matta JA Davini WB Dawe GB Lord B Bredt DS (2019) α6-Containing nicotinic acetylcholine receptor reconstitution involves mechanistically distinct accessory components. Cell Rep 26:866–874.e3. 30673609 10.1016/j.celrep.2018.12.103

[B207] Guo X Lester RA (2007) Ca2+ flux and signaling implications by nicotinic acetylcholine receptors in rat medial habenula. J Neurophysiol 97:83–92. 17050826 10.1152/jn.01046.2005

[B208] Gutzwiller JP Göke B Drewe J Hildebrand P Ketterer S Handschin D Winterhalder R Conen D Beglinger C (1999) Glucagon-like peptide-1: a potent regulator of food intake in humans. Gut 44:81–86. 9862830 10.1136/gut.44.1.81 PMC1760073

[B209] Harmey D Griffin PR Kenny PJ (2012) Development of novel pharmacotherapeutics for tobacco dependence: progress and future directions. Nicotine Tob Res 14:1300–1318. 23024249 10.1093/ntr/nts201 PMC3611986

[B592] Harpsoe K Ahring PK Christensen JK Jensen ML Peters D Balle T (2011) Unraveling the high- and low-sensitivity agonist responses of nicotinic acetylcholine receptors. J Neurosci 31:10759–10766. 21795528 10.1523/JNEUROSCI.1509-11.2011 PMC6623092

[B210] Harrington L Viñals X Herrera-Solís A Flores A Morel C Tolu S Faure P Maldonado R Maskos U Robledo P (2016) Role of β4* nicotinic acetylcholine receptors in the habenulo-interpeduncular pathway in nicotine reinforcement in mice. Neuropsychopharmacology 41:1790–1802. 26585290 10.1038/npp.2015.346 PMC4869047

[B211] Harris AC Muelken P Swain Y Palumbo M Jain V Goniewicz ML Stepanov I LeSage MG (2019) Non-nicotine constituents in e-cigarette aerosol extract attenuate nicotine’s aversive effects in adolescent rats. Drug Alcohol Depend 203:51–60. 31404849 10.1016/j.drugalcdep.2019.05.023 PMC6941564

[B212] Harrison AA Gasparini F Markou A (2002) Nicotine potentiation of brain stimulation reward reversed by DH β E and SCH 23390, but not by eticlopride, LY 314582 or MPEP in rats. Psychopharmacology (Berl) 160:56–66. 11862374 10.1007/s00213-001-0953-6

[B213] Harvey DM Yasar S Heishman SJ Panlilio LV Henningfield JE Goldberg SR (2004) Nicotine serves as an effective reinforcer of intravenous drug-taking behavior in human cigarette smokers. Psychopharmacology (Berl) 175:134–142. 14997277 10.1007/s00213-004-1818-6

[B214] Harvey SC Luetje CW (1996) Determinants of competitive antagonist sensitivity on neuronal nicotinic receptor beta subunits. J Neurosci 16:3798–3806. 8656274 10.1523/JNEUROSCI.16-12-03798.1996 PMC6578601

[B215] Harvey SC Maddox FN Luetje CW (1996) Multiple determinants of dihydro-beta-erythroidine sensitivity on rat neuronal nicotinic receptor alpha subunits. J Neurochem 67:1953–1959. 8863500 10.1046/j.1471-4159.1996.67051953.x

[B216] Hayama T Ito S Ogawa H (1985) Responses of solitary tract nucleus neurons to taste and mechanical stimulations of the oral cavity in decerebrate rats. Exp Brain Res 60:235–242. 4054268 10.1007/BF00235918

[B593] Hedman L Backman H Stridsman C Bosson JA Lundbäck M Lindberg A Rönmark E Ekerljung L (2018) Association of electronic cigarette use with smoking habits, demographic factors, and respiratory symptoms. JAMA Netw Open 1:e180789. 30646032 10.1001/jamanetworkopen.2018.0789 PMC6324524

[B219] Henderson BJ Wall TR Henley BM Kim CH McKinney S Lester HA (2017) Menthol Enhances Nicotine Reward-Related Behavior by Potentiating Nicotine-Induced Changes in nAChR Function, nAChR Upregulation, and DA Neuron Excitability. Neuropsychopharmacology 42:2285–2291. 28401925 10.1038/npp.2017.72 PMC5645749

[B220] Henningfield JE Goldberg SR (1983) Nicotine as a reinforcer in human subjects and laboratory animals. Pharmacol Biochem Behav 19:989–992. 6657732 10.1016/0091-3057(83)90405-7

[B221] Hentall ID Gollapudi L (1995) The interpeduncular nucleus regulates nicotine’s effects on free-field activity. Neuroreport 6:2469–2472. 8741744 10.1097/00001756-199512150-00008

[B222] Herkenham M Nauta WJ (1979) Efferent connections of the habenular nuclei in the rat. J Comp Neurol 187:19–47. 226566 10.1002/cne.901870103

[B223] Hicks JH Dani JA Lester RA (2000) Regulation of the sensitivity of acetylcholine receptors to nicotine in rat habenula neurons. J Physiol 529:579–597. 11118491 10.1111/j.1469-7793.2000.00579.x PMC2270233

[B224] Higa KK Grim A Kamenski ME, (2017) Nicotine withdrawal-induced inattention is absent in alpha7 nAChR knockout mice. Psychopharmacology (Berl) 234:1573–1586. 28243714 10.1007/s00213-017-4572-2 PMC5420484

[B594] Hildebrand BE Nomikos GG Bondjers C Nisell M Svensson TH (1997) Behavioral manifestations of the nicotine abstinence syndrome in the rat: peripheral versus central mechanisms. Psychopharmacology (Berl) 129:348–356. 9085404 10.1007/s002130050200

[B225] Hildebrand BE Nomikos GG Hertel P Schilström B Svensson TH (1998) Reduced dopamine output in the nucleus accumbens but not in the medial prefrontal cortex in rats displaying a mecamylamine-precipitated nicotine withdrawal syndrome. Brain Res 779:214–225. 9473676 10.1016/s0006-8993(97)01135-9

[B226] Hildebrand BE Panagis G Svensson TH Nomikos GG (1999) Behavioral and biochemical manifestations of mecamylamine-precipitated nicotine withdrawal in the rat: role of nicotinic receptors in the ventral tegmental area. Neuropsychopharmacology 21:560–574. 10481840 10.1016/S0893-133X(99)00055-X

[B227] Hines T Toney GM Mifflin SW (1994) Responses of neurons in the nucleus tractus solitarius to stimulation of heart and lung receptors in the rat. Circ Res 74:1188–1196. 8187285 10.1161/01.res.74.6.1188

[B228] Hnasko TS Hjelmstad GO Fields HL Edwards RH (2012) Ventral tegmental area glutamate neurons: electrophysiological properties and projections. J Neurosci 32:15076–15085. 23100428 10.1523/JNEUROSCI.3128-12.2012 PMC3685320

[B229] Hong S Jhou TC Smith M Saleem KS Hikosaka O (2011) Negative reward signals from the lateral habenula to dopamine neurons are mediated by rostromedial tegmental nucleus in primates. J Neurosci 31:11457–11471. 21832176 10.1523/JNEUROSCI.1384-11.2011 PMC3315151

[B230] Houser CR Crawford GD Barber RP Salvaterra PM Vaughn JE (1983) Organization and morphological characteristics of cholinergic neurons: an immunocytochemical study with a monoclonal antibody to choline acetyltransferase. Brain Res 266:97–119. 6850348 10.1016/0006-8993(83)91312-4

[B231] Hsu YW Morton G Guy EG Wang SD Turner EE (2016) Dorsal Medial Habenula Regulation of Mood-Related Behaviors and Primary Reinforcement by Tachykinin-Expressing Habenula Neurons. eNeuro 3:3. 10.1523/ENEURO.0109-16.2016 PMC4947983 27482535

[B232] Hsu YW Tempest L Quina LA Wei AD Zeng H Turner EE (2013) Medial habenula output circuit mediated by α5 nicotinic receptor-expressing GABAergic neurons in the interpeduncular nucleus. J Neurosci 33:18022–18035. 24227714 10.1523/JNEUROSCI.2927-13.2013 PMC3828458

[B233] Hu F Ren J Zhang JE Zhong W Luo M (2012) Natriuretic peptides block synaptic transmission by activating phosphodiesterase 2A and reducing presynaptic PKA activity. Proc Natl Acad Sci USA 109:17681–17686. 23045693 10.1073/pnas.1209185109 PMC3491473

[B234] Hucho F Changeux JP (1973) Molecular weight and quaternary structure of the cholinergic receptor protein extracted by detergents from Electrophorus electricus electric tissue. FEBS Lett 38:11–15. 4772687 10.1016/0014-5793(73)80500-9

[B235] Huganir RL Delcour AH Greengard P Hess GP (1986) Phosphorylation of the nicotinic acetylcholine receptor regulates its rate of desensitization. Nature 321:774–776. 2423885 10.1038/321774a0

[B236] Hughes JR Gust SW Skoog K Keenan RM Fenwick JW (1991) Symptoms of tobacco withdrawal. A replication and extension. Arch Gen Psychiatry 48:52–59. 1984762 10.1001/archpsyc.1991.01810250054007

[B237] Hung RJ McKay JD Gaborieau V Boffetta P Hashibe M Zaridze D Mukeria A Szeszenia-Dabrowska N Lissowska J Rudnai P, (2008) A susceptibility locus for lung cancer maps to nicotinic acetylcholine receptor subunit genes on 15q25. Nature 452:633–637. 18385738 10.1038/nature06885

[B238] Hussain RJ Taraschenko OD Glick SD (2008) Effects of nicotine, methamphetamine and cocaine on extracellular levels of acetylcholine in the interpeduncular nucleus of rats. Neurosci Lett 440:270–274. 18583043 10.1016/j.neulet.2008.06.001 PMC3742071

[B239] Husson M Harrington L Tochon L Cho Y Ibañez-Tallon I Maskos U David V (2020) β4-Nicotinic Receptors Are Critically Involved in Reward-Related Behaviors and Self-Regulation of Nicotine Reinforcement. J Neurosci 40:3465–3477. 32184221 10.1523/JNEUROSCI.0356-19.2020 PMC7178913

[B240] Huston-Lyons D Sarkar M Kornetsky C (1993) Nicotine and brain-stimulation reward: interactions with morphine, amphetamine and pimozide. Pharmacol Biochem Behav 46:453–457. 8265701 10.1016/0091-3057(93)90378-7

[B241] Ikemoto S Qin M Liu ZH (2006) Primary reinforcing effects of nicotine are triggered from multiple regions both inside and outside the ventral tegmental area. J Neurosci 26:723–730. 16421292 10.1523/JNEUROSCI.4542-05.2006 PMC1380251

[B242] Ikemoto S Wise RA (2002) Rewarding effects of the cholinergic agents carbachol and neostigmine in the posterior ventral tegmental area. J Neurosci 22:9895–9904. 12427846 10.1523/JNEUROSCI.22-22-09895.2002 PMC6757811

[B243] Ikemoto S Witkin BM Morales M (2003) Rewarding injections of the cholinergic agonist carbachol into the ventral tegmental area induce locomotion and c-Fos expression in the retrosplenial area and supramammillary nucleus. Brain Res 969:78–87. 12676367 10.1016/s0006-8993(03)02280-7

[B244] Imperato A Mulas A Di Chiara G (1986) Nicotine preferentially stimulates dopamine release in the limbic system of freely moving rats. Eur J Pharmacol 132:337–338. 3816984 10.1016/0014-2999(86)90629-1

[B245] Ishibashi M Leonard CS Kohlmeier KA (2009) Nicotinic activation of laterodorsal tegmental neurons: implications for addiction to nicotine. Neuropsychopharmacology 34:2529–2547. 19625996 10.1038/npp.2009.82 PMC2762000

[B246] Ivanová S Greenshaw AJ (1997) Nicotine-induced decreases in VTA electrical self-stimulation thresholds: blockade by haloperidol and mecamylamine but not scopolamine or ondansetron. Psychopharmacology (Berl) 134:187–192. 9399383 10.1007/s002130050441

[B247] Iwasaki K Sato M (1981) Neural responses and aversion to bitter stimuli in rats. Chem Senses 6:119–128.

[B248] Iyaniwura TT Wright AE Balfour DJ (2001) Evidence that mesoaccumbens dopamine and locomotor responses to nicotine in the rat are influenced by pretreatment dose and strain. Psychopharmacology (Berl) 158:73–79. 11685386 10.1007/s002130100852

[B249] Jackson A Papke RL Damaj MI (2018) Pharmacological modulation of the α7 nicotinic acetylcholine receptor in a mouse model of mecamylamine-precipitated nicotine withdrawal. Psychopharmacology (Berl) 235:1897–1905. 29549391 10.1007/s00213-018-4879-7 PMC6015775

[B250] Jackson AB Toma W Contreras KM Alkhlaif Y Damaj MI (2019) The β3 subunit of the nicotinic acetylcholine receptor is required for nicotine withdrawal-induced affective but not physical signs or nicotine reward in mice. Pharmacol Biochem Behav 183:1–5. 31145916 10.1016/j.pbb.2019.05.003 PMC7197262

[B251] Jackson KJ Marks MJ Vann RE Chen X Gamage TF Warner JA Damaj MI (2010) Role of alpha5 nicotinic acetylcholine receptors in pharmacological and behavioral effects of nicotine in mice. J Pharmacol Exp Ther 334:137–146. 20400469 10.1124/jpet.110.165738 PMC2912049

[B252] Jackson KJ Martin BR Changeux JP Damaj MI (2008) Differential role of nicotinic acetylcholine receptor subunits in physical and affective nicotine withdrawal signs. J Pharmacol Exp Ther 325:302–312. 18184829 10.1124/jpet.107.132977 PMC3821841

[B253] Jackson KJ McIntosh JM Brunzell DH Sanjakdar SS Damaj MI (2009) The role of alpha6-containing nicotinic acetylcholine receptors in nicotine reward and withdrawal. J Pharmacol Exp Ther 331:547–554. 19644040 10.1124/jpet.109.155457 PMC2775251

[B254] Jain A Kuryatov A Wang J Kamenecka TM Lindstrom J (2016) Unorthodox Acetylcholine Binding Sites Formed by α5 and β3 Accessory Subunits in α4β2* Nicotinic Acetylcholine Receptors. J Biol Chem 291:23452–23463. 27645992 10.1074/jbc.M116.749150 PMC5095401

[B255] Jennings C Gosnell S Curtis KN Kosten T Salas R (2020) Altered habenula resting state functional connectivity in deprived veteran tobacco smokers: A pilot study. Bull Menninger Clin 84:21–34. 31939683 10.1521/bumc_2020_84_02

[B256] Jensen KP DeVito EE Herman AI Valentine GW Gelernter J Sofuoglu M (2015) A CHRNA5 Smoking Risk Variant Decreases the Aversive Effects of Nicotine in Humans. Neuropsychopharmacology 40:2813–2821. 25948103 10.1038/npp.2015.131 PMC4864657

[B257] Jhou TC Fields HL Baxter MG Saper CB Holland PC (2009a) The rostromedial tegmental nucleus (RMTg), a GABAergic afferent to midbrain dopamine neurons, encodes aversive stimuli and inhibits motor responses. Neuron 61:786–800. 19285474 10.1016/j.neuron.2009.02.001 PMC2841475

[B258] Jhou TC Geisler S Marinelli M Degarmo BA Zahm DS (2009b) The mesopontine rostromedial tegmental nucleus: A structure targeted by the lateral habenula that projects to the ventral tegmental area of Tsai and substantia nigra compacta. J Comp Neurol 513:566–596. 19235216 10.1002/cne.21891 PMC3116663

[B259] Jin XT Tucker BR Drenan RM (2020) Nicotine Self-Administration Induces Plastic Changes to Nicotinic Receptors in Medial Habenula. eNeuro 7:7. 10.1523/ENEURO.0197-20.2020 PMC7405075 32675176

[B260] Johnson JH Zhao C James JR Rosecrans JA (2000) Individual variability of dopamine release from nucleus accumbens induced by nicotine. Brain Res Bull 51:249–253. 10718517 10.1016/s0361-9230(99)00226-9

[B261] Jonkman S Markou A (2006) Blockade of nicotinic acetylcholine or dopamine D1-like receptors in the central nucleus of the amygdala or the bed nucleus of the stria terminalis does not precipitate nicotine withdrawal in nicotine-dependent rats. Neurosci Lett 400:140–145. 16563623 10.1016/j.neulet.2006.02.030

[B262] Justice AE Winkler TW Feitosa MF Graff M Fisher VA Young K Barata L Deng X Czajkowski J Hadley D, (2017) Genome-wide meta-analysis of 241,258 adults accounting for smoking behaviour identifies novel loci for obesity traits. Nat Commun 8:14977. 28443625 10.1038/ncomms14977 PMC5414044

[B263] Kaiser SA Soliakov L Harvey SC Luetje CW Wonnacott S (1998) Differential inhibition by alpha-conotoxin-MII of the nicotinic stimulation of [3H]dopamine release from rat striatal synaptosomes and slices. J Neurochem 70:1069–1076. 9489727 10.1046/j.1471-4159.1998.70031069.x

[B264] Kemp G Morley BJ (1986) Ganglionic nAChRs and high-affinity nicotinic binding sites are not equivalent. FEBS Lett 205:265–268. 3743777 10.1016/0014-5793(86)80910-3

[B265] Kenny PJ Chartoff E Roberto M Carlezon WA Jr Markou A (2009) NMDA receptors regulate nicotine-enhanced brain reward function and intravenous nicotine self-administration: role of the ventral tegmental area and central nucleus of the amygdala. Neuropsychopharmacology 34:266–281. 18418357 10.1038/npp.2008.58 PMC2654386

[B266] Kenny PJ Cheeta S File SE (2000) Anxiogenic effects of nicotine in the dorsal hippocampus are mediated by 5-HT1A and not by muscarinic M1 receptors. Neuropharmacology 39:300–307. 10670425 10.1016/s0028-3908(99)00114-8

[B267] Kenny PJ Gasparini F Markou A (2003) Group II metabotropic and alpha-amino-3-hydroxy-5-methyl-4-isoxazole propionate (AMPA)/kainate glutamate receptors regulate the deficit in brain reward function associated with nicotine withdrawal in rats. J Pharmacol Exp Ther 306:1068–1076. 12805481 10.1124/jpet.103.052027

[B268] Kenny PJ Markou A (2001) Neurobiology of the nicotine withdrawal syndrome. Pharmacol Biochem Behav 70:531–549. 11796152 10.1016/s0091-3057(01)00651-7

[B269] Kenny PJ Markou A (2006) Nicotine self-administration acutely activates brain reward systems and induces a long-lasting increase in reward sensitivity. Neuropsychopharmacology 31:1203–1211. 16192981 10.1038/sj.npp.1300905

[B270] Kim U Chang SY (2005) Dendritic morphology, local circuitry, and intrinsic electrophysiology of neurons in the rat medial and lateral habenular nuclei of the epithalamus. J Comp Neurol 483:236–250. 15678472 10.1002/cne.20410

[B271] Kimura H McGeer PL Peng JH McGeer EG (1981) The central cholinergic system studied by choline acetyltransferase immunohistochemistry in the cat. J Comp Neurol 200:151–201. 7287919 10.1002/cne.902000202

[B272] Kiyatkin EA (2014) Critical role of peripheral sensory systems in mediating the neural effects of nicotine following its acute and repeated exposure. Rev Neurosci 25:207–221. 24535300 10.1515/revneuro-2013-0067 PMC4529070

[B273] Klink R de Kerchove d’Exaerde A Zoli M Changeux JP (2001) Molecular and physiological diversity of nicotinic acetylcholine receptors in the midbrain dopaminergic nuclei. J Neurosci 21:1452–1463. 11222635 10.1523/JNEUROSCI.21-05-01452.2001 PMC6762941

[B274] Knight, C. Howard, P. Baker, C. L. Marton, J. P. (2009) The Cost-Effectiveness of an Extended Course (12 + 12 Weeks) of Varenicline Compared with Other Available Smoking Cessation Strategies in the United States: An Extension and Update to the BENESCO Model. Value Health. 10.1111/j.1524-4733.2009.00672.x 19912599

[B275] Kobayashi Y Sano Y Vannoni E Goto H Suzuki H Oba A Kawasaki H Kanba S Lipp HP Murphy NP, (2013) Genetic dissection of medial habenula-interpeduncular nucleus pathway function in mice. Front Behav Neurosci 7:17. 23487260 10.3389/fnbeh.2013.00017 PMC3594921

[B276] Koob GF Bloom FE (1988) Cellular and molecular mechanisms of drug dependence. Science 242:715–723. 2903550 10.1126/science.2903550

[B277] Koob GF Le Moal M (1997) Drug abuse: hedonic homeostatic dysregulation. Science 278:52–58. 9311926 10.1126/science.278.5335.52

[B278] Koppensteiner P Melani R Ninan I (2017) A Cooperative Mechanism Involving Ca^2+^-Permeable AMPA Receptors and Retrograde Activation of GABA_B_ Receptors in Interpeduncular Nucleus Plasticity. Cell Rep 20:1111–1122. 28768196 10.1016/j.celrep.2017.07.013 PMC5568868

[B279] Kulak JM Nguyen TA Olivera BM McIntosh JM (1997) Alpha-conotoxin MII blocks nicotine-stimulated dopamine release in rat striatal synaptosomes. J Neurosci 17:5263–5270. 9204910 10.1523/JNEUROSCI.17-14-05263.1997 PMC6793802

[B280] Kuryatov A Berrettini W Lindstrom J (2011) Acetylcholine receptor (AChR) α5 subunit variant associated with risk for nicotine dependence and lung cancer reduces (α4β2)_2_α5 AChR function. Mol Pharmacol 79:119–125. 20881005 10.1124/mol.110.066357 PMC3014277

[B281] Kuryatov A Onksen J Lindstrom J (2008) Roles of accessory subunits in alpha4beta2(*) nicotinic receptors. Mol Pharmacol 74:132–143. 18381563 10.1124/mol.108.046789

[B282] Lai A Parameswaran N Khwaja M Whiteaker P Lindstrom JM Fan H McIntosh JM Grady SR Quik M (2005) Long-term nicotine treatment decreases striatal alpha 6* nicotinic acetylcholine receptor sites and function in mice. Mol Pharmacol 67:1639–1647. 15681595 10.1124/mol.104.006429

[B283] Lammel S Hetzel A Häckel O Jones I Liss B Roeper J (2008) Unique properties of mesoprefrontal neurons within a dual mesocorticolimbic dopamine system. Neuron 57:760–773. 18341995 10.1016/j.neuron.2008.01.022

[B284] Lammel S Ion DI Roeper J Malenka RC (2011) Projection-specific modulation of dopamine neuron synapses by aversive and rewarding stimuli. Neuron 70:855–862. 21658580 10.1016/j.neuron.2011.03.025 PMC3112473

[B285] Lammel, S. Lim, B. K. Malenka, R. C. (2014) Reward and aversion in a heterogeneous midbrain dopamine system. Neuropharmacology 76 Pt B, 351-359. 23578393 10.1016/j.neuropharm.2013.03.019 PMC3778102

[B286] Lammel S Lim BK Ran C Huang KW Betley MJ Tye KM Deisseroth K Malenka RC (2012) Input-specific control of reward and aversion in the ventral tegmental area. Nature 491:212–217. 23064228 10.1038/nature11527 PMC3493743

[B287] Lammel S Steinberg EE Földy C Wall NR Beier K Luo L Malenka RC (2015) Diversity of transgenic mouse models for selective targeting of midbrain dopamine neurons. Neuron 85:429–438. 25611513 10.1016/j.neuron.2014.12.036 PMC5037114

[B288] Laviolette SR Alexson TO van der Kooy D (2002) Lesions of the tegmental pedunculopontine nucleus block the rewarding effects and reveal the aversive effects of nicotine in the ventral tegmental area. J Neurosci 22:8653–8660. 12351739 10.1523/JNEUROSCI.22-19-08653.2002 PMC6757774

[B289] Laviolette SR Lauzon NM Bishop SF Sun N Tan H (2008) Dopamine signaling through D1-like versus D2-like receptors in the nucleus accumbens core versus shell differentially modulates nicotine reward sensitivity. J Neurosci 28:8025–8033. 18685027 10.1523/JNEUROSCI.1371-08.2008 PMC6670771

[B290] Laviolette, S. R. van der Kooy, D. (2003a) Blockade of mesolimbic dopamine transmission dramatically increases sensitivity to the rewarding effects of nicotine in the ventral tegmental area. Mol Psychiatry 8, 50-59, 59. 12556908 10.1038/sj.mp.4001197

[B291] Laviolette SR van der Kooy D (2003b) The motivational valence of nicotine in the rat ventral tegmental area is switched from rewarding to aversive following blockade of the alpha7-subunit-containing nicotinic acetylcholine receptor. Psychopharmacology (Berl) 166:306–313. 12569428 10.1007/s00213-002-1317-6

[B292] Le Foll B Wertheim C Goldberg SR (2007) High reinforcing efficacy of nicotine in non-human primates. PLoS One 2:e230. 17311094 10.1371/journal.pone.0000230 PMC1794142

[B293] Le Novère N Corringer PJ Changeux JP (2002) The diversity of subunit composition in nAChRs: evolutionary origins, physiologic and pharmacologic consequences. J Neurobiol 53:447–456. 12436412 10.1002/neu.10153

[B294] Le Novère N Zoli M Changeux JP (1996) Neuronal nicotinic receptor alpha 6 subunit mRNA is selectively concentrated in catecholaminergic nuclei of the rat brain. Eur J Neurosci 8:2428–2439. 8950106 10.1111/j.1460-9568.1996.tb01206.x

[B295] Lecca D Cacciapaglia F Valentini V Gronli J Spiga S Di Chiara G (2006) Preferential increase of extracellular dopamine in the rat nucleus accumbens shell as compared to that in the core during acquisition and maintenance of intravenous nicotine self-administration. Psychopharmacology (Berl) 184:435–446. 16397746 10.1007/s00213-005-0280-4

[B296] Lecca S Melis M Luchicchi A Ennas MG Castelli MP Muntoni AL Pistis M (2011) Effects of drugs of abuse on putative rostromedial tegmental neurons, inhibitory afferents to midbrain dopamine cells. Neuropsychopharmacology 36:589–602. 21048703 10.1038/npp.2010.190 PMC3055682

[B297] Lemon CH Smith DV (2005) Neural representation of bitter taste in the nucleus of the solitary tract. J Neurophysiol 94:3719–3729. 16107527 10.1152/jn.00700.2005

[B298] Léna C Changeux JP (1998) Allosteric nicotinic receptors, human pathologies. J Physiol Paris 92:63–74. 9782446 10.1016/S0928-4257(98)80140-X

[B299] Léna C Changeux JP Mulle C (1993) Evidence for “preterminal” nicotinic receptors on GABAergic axons in the rat interpeduncular nucleus. J Neurosci 13:2680–2688. 8501532 10.1523/JNEUROSCI.13-06-02680.1993 PMC6576498

[B300] Lenn NJ (1976) Synapses in the interpeduncular nucleus: electron microscopy of normal and habenula lesioned rats. J Comp Neurol 166:77–99. 1262550 10.1002/cne.901660106

[B301] Lenn NJ Wong V Hamill GS (1983) Left-right pairing at the crest synapses of rat interpeduncular nucleus. Neuroscience 9:383–389. 6877600 10.1016/0306-4522(83)90301-9

[B302] Lenoir M Kiyatkin EA (2011) Critical role of peripheral actions of intravenous nicotine in mediating its central effects. Neuropsychopharmacology 36:2125–2138. 21654739 10.1038/npp.2011.104 PMC3158310

[B303] Lerman C LeSage MG Perkins KA O’Malley SS Siegel SJ Benowitz NL Corrigall WA (2007) Translational research in medication development for nicotine dependence. Nat Rev Drug Discov 6:746–762. 17690709 10.1038/nrd2361

[B304] Lester RA Dani JA (1995) Acetylcholine receptor desensitization induced by nicotine in rat medial habenula neurons. J Neurophysiol 74:195–206. 7472323 10.1152/jn.1995.74.1.195

[B305] Leventhal AM Strong DR Kirkpatrick MG Unger JB Sussman S Riggs NR Stone MD Khoddam R Samet JM Audrain-McGovern J (2015) Association of Electronic Cigarette Use With Initiation of Combustible Tobacco Product Smoking in Early Adolescence. JAMA 314:700–707. 26284721 10.1001/jama.2015.8950 PMC4771179

[B306] Levin ED Petro A Rezvani AH Pollard N Christopher NC Strauss M Avery J Nicholson J Rose JE (2009) Nicotinic alpha7- or beta2-containing receptor knockout: effects on radial-arm maze learning and long-term nicotine consumption in mice. Behav Brain Res 196:207–213. 18831991 10.1016/j.bbr.2008.08.048 PMC2638590

[B307] Lima LB Bueno D Leite F Souza S Gonçalves L Furigo IC Donato J Jr Metzger M (2017) Afferent and efferent connections of the interpeduncular nucleus with special reference to circuits involving the habenula and raphe nuclei. J Comp Neurol 525:2411–2442. 28340505 10.1002/cne.24217

[B308] Lips EH Gaborieau V McKay JD, (2009) Association between a 15q25 gene variant, smoking quantity and tobacco-related cancers among 17 000 individuals. Int J Epidemiol 39:563–77. 19776245 10.1093/ije/dyp288 PMC2913450

[B309] Lisoprawski A Blanc G Glowinski J (1981) Activation by stress of the habenulo-interpeduncular substance P neurons in the rat. Neurosci Lett 25:47–51. 6168982 10.1016/0304-3940(81)90099-9

[B310] Liu C Bonaventure P Lee G Nepomuceno D Kuei C Wu J Li Q Joseph V Sutton SW Eckert W, (2015) GPR139, an orphan receptor highly enriched in the habenula and septum, is activated by the essential amino acids L-tryptophan and L-phenylalanine. Mol Pharmacol 88:911–925. 26349500 10.1124/mol.115.100412

[B311] Liu L Zhao-Shea R McIntosh JM Gardner PD Tapper AR (2012) Nicotine persistently activates ventral tegmental area dopaminergic neurons via nicotinic acetylcholine receptors containing α4 and α6 subunits. Mol Pharmacol 81:541–548. 22222765 10.1124/mol.111.076661 PMC3310415

[B312] Liu Q Han H Wang M Yao Y Wen L Jiang K Ma Y Fan R Chen J Su K, (2018) Association and cis-mQTL analysis of variants in CHRNA3-A5, CHRNA7, CHRNB2, and CHRNB4 in relation to nicotine dependence in a Chinese Han population. Transl Psychiatry 8:83. 29666375 10.1038/s41398-018-0130-x PMC5904126

[B313] Liu X Koren AO Yee SK Pechnick RN Poland RE London ED (2003) Self-administration of 5-iodo-A-85380, a beta2-selective nicotinic receptor ligand, by operantly trained rats. Neuroreport 14:1503–1505. 12960773 10.1097/00001756-200308060-00020

[B314] Liu Z Habener JF (2008) Glucagon-like peptide-1 activation of TCF7L2-dependent Wnt signaling enhances pancreatic beta cell proliferation. J Biol Chem 283:8723–8735. 18216022 10.1074/jbc.M706105200 PMC2417166

[B315] Lodge DJ Grace AA (2006) The laterodorsal tegmentum is essential for burst firing of ventral tegmental area dopamine neurons. Proc Natl Acad Sci USA 103:5167–5172. 16549786 10.1073/pnas.0510715103 PMC1458812

[B316] Lotfipour S Byun JS Leach P Fowler CD Murphy NP Kenny PJ Gould TJ Boulter J (2013) Targeted deletion of the mouse α2 nicotinic acetylcholine receptor subunit gene (Chrna2) potentiates nicotine-modulated behaviors. J Neurosci 33:7728–7741. 23637165 10.1523/JNEUROSCI.4731-12.2013 PMC3831006

[B317] Lynch WJ Carroll ME (1999) Regulation of intravenously self-administered nicotine in rats. Exp Clin Psychopharmacol 7:198–207. 10472507 10.1037//1064-1297.7.3.198

[B318] Lynch WJ Carroll ME (2001) Regulation of drug intake. Exp Clin Psychopharmacol 9:131–143. 11518086 10.1037//1064-1297.9.2.131

[B319] Madsen HB Koghar HS Pooters T Massalas JS Drago J Lawrence AJ (2015) Role of α4- and α6-containing nicotinic receptors in the acquisition and maintenance of nicotine self-administration. Addict Biol 20:500–512. 24750355 10.1111/adb.12148

[B320] Malin DH Lake JR Newlin-Maultsby P Roberts LK Lanier JG Carter VA Cunningham JS Wilson OB (1992) Rodent model of nicotine abstinence syndrome. Pharmacol Biochem Behav 43:779–784. 1448472 10.1016/0091-3057(92)90408-8

[B321] Mameli-Engvall M Evrard A Pons S Maskos U Svensson TH Changeux JP Faure P (2006) Hierarchical control of dopamine neuron-firing patterns by nicotinic receptors. Neuron 50:911–921. 16772172 10.1016/j.neuron.2006.05.007

[B322] Mansvelder HD Keath JR McGehee DS (2002) Synaptic mechanisms underlie nicotine-induced excitability of brain reward areas. Neuron 33:905–919. 11906697 10.1016/s0896-6273(02)00625-6

[B323] Mansvelder HD McGehee DS (2000) Long-term potentiation of excitatory inputs to brain reward areas by nicotine. Neuron 27:349–357. 10985354 10.1016/s0896-6273(00)00042-8

[B324] Mao D Perry DC Yasuda RP Wolfe BB Kellar KJ (2008) The alpha4beta2alpha5 nicotinic cholinergic receptor in rat brain is resistant to up-regulation by nicotine in vivo. J Neurochem 104:446–456. 17961152 10.1111/j.1471-4159.2007.05011.x

[B325] Markou A Kosten TR Koob GF (1998) Neurobiological similarities in depression and drug dependence: a self-medication hypothesis. Neuropsychopharmacology 18:135–174. 9471114 10.1016/S0893-133X(97)00113-9

[B326] Markou A Paterson NE (2001) The nicotinic antagonist methyllycaconitine has differential effects on nicotine self-administration and nicotine withdrawal in the rat. Nicotine Tob Res 3:361–373. 11694204 10.1080/14622200110073380

[B327] Marks MJ Burch JB Collins AC (1983) Effects of chronic nicotine infusion on tolerance development and nicotinic receptors. J Pharmacol Exp Ther 226:817–825. 6887012

[B328] Marks MJ Grady SR Salminen O Paley MA Wageman CR McIntosh JM Whiteaker P (2014) α6β2*-subtype nicotinic acetylcholine receptors are more sensitive than α4β2*-subtype receptors to regulation by chronic nicotine administration. J Neurochem 130:185–198. 24661093 10.1111/jnc.12721 PMC4107044

[B329] Marks MJ Meinerz NM Drago J Collins AC (2007) Gene targeting demonstrates that alpha4 nicotinic acetylcholine receptor subunits contribute to expression of diverse [3H]epibatidine binding sites and components of biphasic 86Rb+ efflux with high and low sensitivity to stimulation by acetylcholine. Neuropharmacology 53:390–405. 17631923 10.1016/j.neuropharm.2007.05.021 PMC2577786

[B330] Marks MJ Pauly JR Gross SD Deneris ES Hermans-Borgmeyer I Heinemann SF Collins AC (1992) Nicotine binding and nicotinic receptor subunit RNA after chronic nicotine treatment. J Neurosci 12:2765–2784. 1613557 10.1523/JNEUROSCI.12-07-02765.1992 PMC6575859

[B331] Marques-Vidal P Kutalik Z Paccaud F Bergmann S Waeber G Vollenweider P Cornuz J (2011) Variant within the promoter region of the CHRNA3 gene associated with FTN dependence is not related to self-reported willingness to quit smoking. Nicotine Tob Res 13:833–839. 21511889 10.1093/ntr/ntr084

[B332] Marshall DL Redfern PH Wonnacott S (1997) Presynaptic nicotinic modulation of dopamine release in the three ascending pathways studied by in vivo microdialysis: comparison of naive and chronic nicotine-treated rats. J Neurochem 68:1511–1519. 9084421 10.1046/j.1471-4159.1997.68041511.x

[B333] Marubio LM Gardier AM Durier S David D Klink R Arroyo-Jimenez MM McIntosh JM Rossi F Champtiaux N Zoli M, (2003) Effects of nicotine in the dopaminergic system of mice lacking the alpha4 subunit of neuronal nicotinic acetylcholine receptors. Eur J Neurosci 17:1329–1337. 12713636 10.1046/j.1460-9568.2003.02564.x

[B334] Maskos U (2008) The cholinergic mesopontine tegmentum is a relatively neglected nicotinic master modulator of the dopaminergic system: relevance to drugs of abuse and pathology. Br J Pharmacol 153 (Suppl 1):S438–S445. 18223661 10.1038/bjp.2008.5 PMC2268063

[B335] Maskos U Molles BE Pons S Besson M Guiard BP Guilloux JP Evrard A Cazala P Cormier A Mameli-Engvall M, (2005) Nicotine reinforcement and cognition restored by targeted expression of nicotinic receptors. Nature 436:103–107. 16001069 10.1038/nature03694

[B336] Mathers CD Loncar D (2006) Projections of global mortality and burden of disease from 2002 to 2030. PLoS Med 3:e442. 17132052 10.1371/journal.pmed.0030442 PMC1664601

[B337] Maurer JJ Sandager-Nielsen K Schmidt HD (2017) Attenuation of nicotine taking and seeking in rats by the stoichiometry-selective alpha4beta2 nicotinic acetylcholine receptor positive allosteric modulator NS9283. Psychopharmacology (Berl) 234:475–484. 27844094 10.1007/s00213-016-4475-7

[B338] Mazzaferro S Benallegue N Carbone A Gasparri F Vijayan R Biggin PC Moroni M Bermudez I (2011) Additional acetylcholine (ACh) binding site at alpha4/alpha4 interface of (alpha4beta2)2alpha4 nicotinic receptor influences agonist sensitivity. J Biol Chem 286:31043–31054. 21757735 10.1074/jbc.M111.262014 PMC3162463

[B339] Mazzaferro S Bermudez I Sine SM (2019) Potentiation of a neuronal nicotinic receptor via pseudo-agonist site. Cell Mol Life Sci 76:1151–1167. 30600358 10.1007/s00018-018-2993-7 PMC8022356

[B340] McCallum SE Cowe MA Lewis SW Glick SD (2012) α3β4 nicotinic acetylcholine receptors in the medial habenula modulate the mesolimbic dopaminergic response to acute nicotine in vivo. Neuropharmacology 63:434–440. 22561751 10.1016/j.neuropharm.2012.04.015 PMC3381928

[B341] McClernon FJ Froeliger B Rose JE Kozink RV Addicott MA Sweitzer MM Westman EC Van Wert DM (2016) The effects of nicotine and non-nicotine smoking factors on working memory and associated brain function. Addict Biol 21:954–961. 25904425 10.1111/adb.12253 PMC4618271

[B342] McGehee DS Heath MJ Gelber S Devay P Role LW (1995) Nicotine enhancement of fast excitatory synaptic transmission in CNS by presynaptic receptors. Science 269:1692–1696. 7569895 10.1126/science.7569895

[B343] McGovern DJ Polter AM Root DH(2020) Neurochemical signaling of reward and aversion by ventral tegmental area glutamate neurons. In: bioRxiv. 10.1523/JNEUROSCI.1419-20.2021 PMC8221607 34001626

[B344] McGranahan TM Patzlaff NE Grady SR Heinemann SF Booker TK (2011) α4β2 nicotinic acetylcholine receptors on dopaminergic neurons mediate nicotine reward and anxiety relief. J Neurosci 31:10891–10902. 21795541 10.1523/JNEUROSCI.0937-11.2011 PMC3539812

[B345] Melani R Von Itter R Jing D Koppensteiner P Ninan I (2019) Opposing effects of an atypical glycinergic and substance P transmission on interpeduncular nucleus plasticity. Neuropsychopharmacology 44:1828–1836. 31005058 10.1038/s41386-019-0396-6 PMC6785085

[B346] Metzger M Souza R Lima LB Bueno D Goncalves L Sego C Donato J Jr Shammah-Lagnado SJ (2019) Habenular connections with the dopaminergic and serotonergic system and their role in stress-related psychiatric disorders. Eur J Neurosci 53:65–88. 31833616 10.1111/ejn.14647

[B347] Mihalak KB Carroll FI Luetje CW (2006) Varenicline is a partial agonist at alpha4beta2 and a full agonist at alpha7 neuronal nicotinic receptors. Mol Pharmacol 70:801–805. 16766716 10.1124/mol.106.025130

[B348] Mineur YS Brunzell DH Grady SR Lindstrom JM McIntosh JM Marks MJ King SL Picciotto MR (2009) Localized low-level re-expression of high-affinity mesolimbic nicotinic acetylcholine receptors restores nicotine-induced locomotion but not place conditioning. Genes Brain Behav 8:257–266. 19077117 10.1111/j.1601-183X.2008.00468.x PMC2672109

[B349] Ming D Ruiz-Avila L Margolskee RF (1998) Characterization and solubilization of bitter-responsive receptors that couple to gustducin. Proc Natl Acad Sci USA 95:8933–8938. 9671782 10.1073/pnas.95.15.8933 PMC21180

[B350] Moen JK DeBaker MC, Myjak JE Wickman K Lee AM (2021) Bidirectional sex-dependent regulation of α6 and β3 nicotinic acetylcholine receptors by protein kinase Cε. Addict Biol 26:e12954. 32776643 10.1111/adb.12954 PMC7873155

[B351] Mokdad AH Marks JS Stroup DF Gerberding JL (2004) Actual causes of death in the United States, 2000. JAMA 291:1238–1245. 15010446 10.1001/jama.291.10.1238

[B352] Molander L Lunell E Fagerström KO (2000) Reduction of tobacco withdrawal symptoms with a sublingual nicotine tablet: a placebo controlled study. Nicotine Tob Res 2:187–191. 11072457 10.1080/713688123

[B353] Molas S Zhao-Shea R Liu L DeGroot SR Gardner PD Tapper AR (2017) A circuit-based mechanism underlying familiarity signaling and the preference for novelty. Nat Neurosci 20:1260–1268. 28714952 10.1038/nn.4607 PMC5752132

[B354] Mönnikes H Lauer G Arnold R (1997) Peripheral administration of cholecystokinin activates c-fos expression in the locus coeruleus/subcoeruleus nucleus, dorsal vagal complex and paraventricular nucleus via capsaicin-sensitive vagal afferents and CCK-A receptors in the rat. Brain Res 770:277–288. 9372230 10.1016/s0006-8993(97)00865-2

[B355] Monteggia LM Eisch AJ Tang MD Kaczmarek LK Nestler EJ (2000) Cloning and localization of the hyperpolarization-activated cyclic nucleotide-gated channel family in rat brain. Brain Res Mol Brain Res 81:129–139. 11000485 10.1016/s0169-328x(00)00155-8

[B356] Morel C Fattore L Pons S Hay YA Marti F Lambolez B De Biasi M Lathrop M Fratta W Maskos U, (2014) Nicotine consumption is regulated by a human polymorphism in dopamine neurons. Mol Psychiatry 19:930–936. 24296975 10.1038/mp.2013.158 PMC8596967

[B357] Moroni M Zwart R Sher E Cassels BK Bermudez I (2006) alpha4beta2 nicotinic receptors with high and low acetylcholine sensitivity: pharmacology, stoichiometry, and sensitivity to long-term exposure to nicotine. Mol Pharmacol 70:755–768. 16720757 10.1124/mol.106.023044

[B358] Morrison CF Stephenson JA (1972) The occurrence of tolerance to a central depressant effect of nicotine. Br J Pharmacol 46:151–156. 5084817 10.1111/j.1476-5381.1972.tb06857.x PMC1666121

[B359] Morton G Nasirova N Sparks DW Brodsky M Sivakumaran S Lambe EK Turner EE (2018) Chrna5-expressing neurons in the interpeduncular nucleus mediate aversion primed by prior stimulation or nicotine exposure. J Neurosci 38:6900–6920. 29954848 10.1523/JNEUROSCI.0023-18.2018 PMC6070661

[B360] Mugnaini M Garzotti M Sartori I Pilla M Repeto P Heidbreder CA Tessari M (2006) Selective down-regulation of [(125)I]Y0-alpha-conotoxin MII binding in rat mesostriatal dopamine pathway following continuous infusion of nicotine. Neuroscience 137:565–572. 16289885 10.1016/j.neuroscience.2005.09.008

[B361] Mulle C Choquet D Korn H Changeux JP (1992) Calcium influx through nicotinic receptor in rat central neurons: its relevance to cellular regulation. Neuron 8:135–143. 1309647 10.1016/0896-6273(92)90115-t

[B362] Mulle C Vidal C Benoit P Changeux JP (1991) Existence of different subtypes of nicotinic acetylcholine receptors in the rat habenulo-interpeduncular system. J Neurosci 11:2588–2597. 1869929 10.1523/JNEUROSCI.11-08-02588.1991 PMC6575504

[B363] Murray M Murphy CA Ross LL Haun F (1994) The role of the habenula-interpeduncular pathway in modulating levels of circulating adrenal hormones. Restor Neurol Neurosci 6:301–307. 21551761 10.3233/RNN-1994-6406

[B364] Naqvi NH Bechara A (2010) The insula and drug addiction: an interoceptive view of pleasure, urges, and decision-making. Brain Struct Funct 214:435–450. 20512364 10.1007/s00429-010-0268-7 PMC3698865

[B365] Naqvi NH Rudrauf D Damasio H Bechara A (2007) Damage to the insula disrupts addiction to cigarette smoking. Science 315:531–534. 17255515 10.1126/science.1135926 PMC3698854

[B366] Nashmi R Xiao C Deshpande P McKinney S Grady SR Whiteaker P Huang Q McClure-Begley T Lindstrom JM Labarca C, (2007) Chronic nicotine cell specifically upregulates functional alpha 4* nicotinic receptors: basis for both tolerance in midbrain and enhanced long-term potentiation in perforant path. J Neurosci 27:8202–8218. 17670967 10.1523/JNEUROSCI.2199-07.2007 PMC6673074

[B367] Natividad LA Tejeda HA Torres OV O’Dell LE (2010) Nicotine withdrawal produces a decrease in extracellular levels of dopamine in the nucleus accumbens that is lower in adolescent versus adult male rats. Synapse 64:136–145. 19771590 10.1002/syn.20713 PMC2846728

[B368] Naudé J Tolu S Dongelmans M Torquet N Valverde S Rodriguez G Pons S Maskos U Mourot A Marti F, (2016) Nicotinic receptors in the ventral tegmental area promote uncertainty-seeking. Nat Neurosci 19:471–478. 26780509 10.1038/nn.4223

[B369] Nelson ME Kuryatov A Choi CH Zhou Y Lindstrom J (2003) Alternate stoichiometries of alpha4beta2 nicotinic acetylcholine receptors. Mol Pharmacol 63:332–341. 12527804 10.1124/mol.63.2.332

[B370] Nemeth-Coslett R Henningfield JE O’Keeffe MK Griffiths RR (1986) Effects of mecamylamine on human cigarette smoking and subjective ratings. Psychopharmacology (Berl) 88:420–425. 3085131 10.1007/BF00178502

[B371] Neugebauer NM Henehan RM Hales CA Picciotto MR (2011) Mice lacking the galanin gene show decreased sensitivity to nicotine conditioned place preference. Pharmacol Biochem Behav 98:87–93. 21172385 10.1016/j.pbb.2010.12.015 PMC3030658

[B372] Neugebauer NM Zhang Z Crooks PA Dwoskin LP Bardo MT (2006) Effect of a novel nicotinic receptor antagonist, N,N′-dodecane-1,12-diyl-bis-3-picolinium dibromide, on nicotine self-administration and hyperactivity in rats. Psychopharmacology (Berl) 184:426–434. 16220336 10.1007/s00213-005-0163-8

[B373] Ngolab J Liu L Zhao-Shea R Gao G Gardner PD Tapper AR (2015) Functional upregulation of α4* nicotinic acetylcholine receptors in VTA GABAergic neurons increases sensitivity to nicotine reward. J Neurosci 35:8570–8578. 26041923 10.1523/JNEUROSCI.4453-14.2015 PMC4588598

[B374] Niedermaier ON Smith ML Beightol LA Zukowska-Grojec Z Goldstein DS Eckberg DL (1993) Influence of cigarette smoking on human autonomic function. Circulation 88:562–571. 8339419 10.1161/01.cir.88.2.562

[B375] Nisell M Marcus M Nomikos GG Svensson TH (1997) Differential effects of acute and chronic nicotine on dopamine output in the core and shell of the rat nucleus accumbens. J Neural Transm (Vienna) 104:1–10. 9085189 10.1007/BF01271290

[B376] Nisell M Nomikos GG Svensson TH (1994a) Infusion of nicotine in the ventral tegmental area or the nucleus accumbens of the rat differentially affects accumbal dopamine release. Pharmacol Toxicol 75:348–352. 7534921 10.1111/j.1600-0773.1994.tb00373.x

[B377] Nisell M Nomikos GG Svensson TH (1994b) Systemic nicotine-induced dopamine release in the rat nucleus accumbens is regulated by nicotinic receptors in the ventral tegmental area. Synapse 16:36–44. 8134899 10.1002/syn.890160105

[B378] Nomikos GG Hildebrand BE Panagis G Svensson TH (1999) Nicotine withdrawal in the rat: role of alpha7 nicotinic receptors in the ventral tegmental area. Neuroreport 10:697–702. 10208533 10.1097/00001756-199903170-00007

[B379] Nomikos GG Schilström B Hildebrand BE Panagis G Grenhoff J Svensson TH (2000) Role of alpha7 nicotinic receptors in nicotine dependence and implications for psychiatric illness. Behav Brain Res 113:97–103. 10942036 10.1016/s0166-4328(00)00204-7

[B380] Ntamati NR Lüscher C (2016) VTA projection neurons releasing GABA and glutamate in the dentate gyrus. eNeuro 3:3. 10.1523/ENEURO.0137-16.2016 PMC5020313 27648470

[B381] O’Connor EC Parker D Rollema H Mead AN (2010) The alpha4beta2 nicotinic acetylcholine-receptor partial agonist varenicline inhibits both nicotine self-administration following repeated dosing and reinstatement of nicotine seeking in rats. Psychopharmacology (Berl) 208:365–376. 19967529 10.1007/s00213-009-1739-5

[B382] O’Dell LE Bruijnzeel AW Ghozland S Markou A Koob GF (2004) Nicotine withdrawal in adolescent and adult rats. Ann N Y Acad Sci 1021:167–174. 15251887 10.1196/annals.1308.022

[B383] O’Dell LE Torres OV Natividad LA Tejeda HA (2007) Adolescent nicotine exposure produces less affective measures of withdrawal relative to adult nicotine exposure in male rats. Neurotoxicol Teratol 29:17–22. 17184972 10.1016/j.ntt.2006.11.003 PMC3437755

[B384] O’Leary KT Loughlin SE Chen Y Leslie FM (2008) Nicotinic acetylcholine receptor subunit mRNA expression in adult and developing rat medullary catecholamine neurons. J Comp Neurol 510:655–672. 18698592 10.1002/cne.21833

[B385] Oberg M Jaakkola MS Woodward A Peruga A Prüss-Ustün A (2011) Worldwide burden of disease from exposure to second-hand smoke: a retrospective analysis of data from 192 countries. Lancet 377:139–146. 21112082 10.1016/S0140-6736(10)61388-8

[B386] Ogawa, H. Imoto, T. Hayama, T. (1984) Responsiveness of solitario-parabrachial relay neurons to taste and mechanical stimulation applied to the oral cavity in rats. Experimental brain research. Experimentelle Hirnforschung. Experimentation cerebrale 54, 349-358. 6723854 10.1007/BF00236236

[B387] Ohi K Kuwata A Shimada T Kataoka Y Yasuyama T Uehara T Kawasaki Y (2019) Genome-wide variants shared between smoking quantity and schizophrenia on 15q25 are associated with CHRNA5 expression in the brain. Schizophr Bull 45:813–823. 30202994 10.1093/schbul/sby093 PMC6581148

[B388] Olucha-Bordonau FE Teruel V Barcia-González J Ruiz-Torner A Valverde-Navarro AA Martínez-Soriano F (2003) Cytoarchitecture and efferent projections of the nucleus incertus of the rat. J Comp Neurol 464:62–97. 12866129 10.1002/cne.10774

[B389] Orejarena MJ Herrera-Solís A Pons S Maskos U Maldonado R Robledo P (2012) Selective re-expression of β2 nicotinic acetylcholine receptor subunits in the ventral tegmental area of the mouse restores intravenous nicotine self-administration. Neuropharmacology 63:235–241. 22480616 10.1016/j.neuropharm.2012.03.011

[B390] Otsu Y Darcq E Pietrajtis K Mátyás F Schwartz E Bessaih T Abi Gerges S Rousseau CV Grand T Dieudonné S, (2019) Control of aversion by glycine-gated GluN1/GluN3A NMDA receptors in the adult medial habenula. Science 366:250–254. 31601771 10.1126/science.aax1522 PMC7556698

[B391] Otsu Y Lecca S Pietrajtis K Rousseau CV Marcaggi P Dugué GP Mailhes-Hamon C Mameli M Diana MA (2018) Functional principles of posterior septal inputs to the medial habenula. Cell Rep 22:693–705. 29346767 10.1016/j.celrep.2017.12.064 PMC5792424

[B392] Ouagazzal AM Kenny PJ File SE (1999a) Modulation of behaviour on trials 1 and 2 in the elevated plus-maze test of anxiety after systemic and hippocampal administration of nicotine. Psychopharmacology (Berl) 144:54–60. 10379624 10.1007/s002130050976

[B393] Ouagazzal AM Kenny PJ File SE (1999b) Stimulation of nicotinic receptors in the lateral septal nucleus increases anxiety. Eur J Neurosci 11:3957–3962. 10583484 10.1046/j.1460-9568.1999.00823.x

[B394] Oyrer J Bleakley LE Richards KL Maljevic S Phillips AM Petrou S Nowell CJ Reid CA (2019) Using a multiplex nucleic acid *in situ* hybridization technique to determine HCN4 mRNA expression in the adult rodent brain. Front Mol Neurosci 12:211. 31555092 10.3389/fnmol.2019.00211 PMC6724756

[B395] Palma E Maggi L Barabino B Eusebi F Ballivet M (1999) Nicotinic acetylcholine receptors assembled from the alpha7 and beta3 subunits. J Biol Chem 274:18335–18340. 10373437 10.1074/jbc.274.26.18335

[B396] Pang X Liu L Ngolab J Zhao-Shea R McIntosh JM Gardner PD Tapper AR (2016) Habenula cholinergic neurons regulate anxiety during nicotine withdrawal via nicotinic acetylcholine receptors. Neuropharmacology 107:294–304. 27020042 10.1016/j.neuropharm.2016.03.039 PMC4982553

[B397] Pang Y Kiba H Jayaraman A (1993) Acute nicotine injections induce c-fos mostly in non-dopaminergic neurons of the midbrain of the rat. Brain Res Mol Brain Res 20:162–170. 8255178 10.1016/0169-328x(93)90122-6

[B398] Paradiso K Brehm P (1998) Long-term desensitization of nicotinic acetylcholine receptors is regulated via protein kinase A-mediated phosphorylation. J Neurosci 18:9227–9237. 9801362 10.1523/JNEUROSCI.18-22-09227.1998 PMC6792874

[B399] Parker SL Fu Y McAllen K Luo J McIntosh JM Lindstrom JM Sharp BM (2004) Up-regulation of brain nicotinic acetylcholine receptors in the rat during long-term self-administration of nicotine: disproportionate increase of the alpha6 subunit. Mol Pharmacol 65:611–622. 14978239 10.1124/mol.65.3.611

[B400] Parrott AC (1993) Cigarette smoking: effects upon self-rated stress and arousal over the day. Addict Behav 18:389–395. 8213292 10.1016/0306-4603(93)90055-e

[B401] Parsons LH Justice JB Jr (1992) Extracellular concentration and in vivo recovery of dopamine in the nucleus accumbens using microdialysis. J Neurochem 58:212–218. 1727431 10.1111/j.1471-4159.1992.tb09298.x

[B402] Passlick S Thapaliya ER Chen Z Richers MT Ellis-Davies GCR (2018) Optical probing of acetylcholine receptors on neurons in the medial habenula with a novel caged nicotine drug analogue. J Physiol 596:5307–5318. 30222192 10.1113/JP276615 PMC6235938

[B403] Peng C Engle SE Yan Y Weera MM Berry JN Arvin MC Zhao G McIntosh JM Chester JA Drenan RM (2017) Altered nicotine reward-associated behavior following α4 nAChR subunit deletion in ventral midbrain. PLoS One 12:e0182142. 28759616 10.1371/journal.pone.0182142 PMC5536316

[B404] Perez XA Ly J McIntosh JM Quik M (2012) Long-term nicotine exposure depresses dopamine release in nonhuman primate nucleus accumbens. J Pharmacol Exp Ther 342:335–344. 22562772 10.1124/jpet.112.194084 PMC3400796

[B405] Pérez-Morales R González-Zamora A González-Delgado MF Calleros Rincón EY Olivas Calderón EH Martínez-Ramírez OC Rubio J (2018) CHRNA3 rs1051730 and CHRNA5 rs16969968 polymorphisms are associated with heavy smoking, lung cancer, and chronic obstructive pulmonary disease in a mexican population. Ann Hum Genet 82:415–424. 29993116 10.1111/ahg.12264

[B406] Perkins KA Lerman C Coddington S Jetton C Karelitz JL Wilson A Jennings JR Ferrell R Bergen AW Benowitz NL (2008) Gene and gene by sex associations with initial sensitivity to nicotine in nonsmokers. Behav Pharmacol 19:630–640. 18690117 10.1097/FBP.0b013e32830c3621 PMC2743299

[B407] Perry DC Mao D Gold AB McIntosh JM Pezzullo JC Kellar KJ (2007) Chronic nicotine differentially regulates alpha6- and beta3-containing nicotinic cholinergic receptors in rat brain. J Pharmacol Exp Ther 322:306–315. 17446303 10.1124/jpet.107.121228

[B408] Peto R Darby S Deo H Silcocks P Whitley E Doll R (2000) Smoking, smoking cessation, and lung cancer in the UK since 1950: combination of national statistics with two case-control studies. BMJ 321:323–329. 10926586 10.1136/bmj.321.7257.323 PMC27446

[B409] Piasecki TM Fiore MC Baker TB (1998) Profiles in discouragement: two studies of variability in the time course of smoking withdrawal symptoms. J Abnorm Psychol 107:238–251. 9604553 10.1037//0021-843x.107.2.238

[B410] Piasecki TM Jorenby DE Smith SS Fiore MC Baker TB (2003) Smoking withdrawal dynamics: II. Improved tests of withdrawal-relapse relations. J Abnorm Psychol 112:14–27. 12653410

[B411] Piasecki TM Niaura R Shadel WG Abrams D Goldstein M Fiore MC Baker TB (2000) Smoking withdrawal dynamics in unaided quitters. J Abnorm Psychol 109:74–86. 10740938 10.1037//0021-843x.109.1.74

[B412] Picciotto MR Zoli M Rimondini R Léna C Marubio LM Pich EM Fuxe K Changeux JP (1998) Acetylcholine receptors containing the beta2 subunit are involved in the reinforcing properties of nicotine. Nature 391:173–177. 9428762 10.1038/34413

[B413] Pidoplichko VI DeBiasi M Williams JT Dani JA (1997) Nicotine activates and desensitizes midbrain dopamine neurons. Nature 390:401–404. 9389479 10.1038/37120

[B414] Pons S Fattore L Cossu G Tolu S Porcu E McIntosh JM Changeux JP Maskos U Fratta W (2008) Crucial role of alpha4 and alpha6 nicotinic acetylcholine receptor subunits from ventral tegmental area in systemic nicotine self-administration. J Neurosci 28:12318–12327. 19020025 10.1523/JNEUROSCI.3918-08.2008 PMC2819191

[B415] Poorthuis RB Bloem B Verhoog MB Mansvelder HD (2013) Layer-specific interference with cholinergic signaling in the prefrontal cortex by smoking concentrations of nicotine. J Neurosci 33:4843–4853. 23486955 10.1523/JNEUROSCI.5012-12.2013 PMC6618989

[B416] Portugal GS Kenney JW Gould TJ (2008) Beta2 subunit containing acetylcholine receptors mediate nicotine withdrawal deficits in the acquisition of contextual fear conditioning. Neurobiol Learn Mem 89:106–113. 17584502 10.1016/j.nlm.2007.05.002 PMC2276643

[B417] Pritchard WS Robinson JH Guy TD Davis RA Stiles MF (1996) Assessing the sensory role of nicotine in cigarette smoking. Psychopharmacology (Berl) 127:55–62. 8880944 10.1007/BF02805975

[B596] Qi J Zhang S Wang HL Barker DJ Miranda-Barrientos J Morales M (2016) VTA glutamatergic inputs to nucleus accumbens drive aversion by acting on GABAergic interneurons. Nat Neurosci 19:725–733. 27019014 10.1038/nn.4281 PMC4846550

[B418] Qin C Luo M (2009) Neurochemical phenotypes of the afferent and efferent projections of the mouse medial habenula. Neuroscience 161:827–837. 19362132 10.1016/j.neuroscience.2009.03.085

[B419] Qin C Sun Y Chen JD Foreman RD (2005) Gastric electrical stimulation modulates neuronal activity in nucleus tractus solitarii in rats. Auton Neurosci 119:1–8. 15893702 10.1016/j.autneu.2005.01.007

[B420] Quick MW Ceballos RM Kasten M McIntosh JM Lester RA (1999) Alpha3beta4 subunit-containing nicotinic receptors dominate function in rat medial habenula neurons. Neuropharmacology 38:769–783. 10465681 10.1016/s0028-3908(99)00024-6

[B421] Quik M Polonskaya Y Gillespie A Jakowec M Lloyd GK Langston JW (2000) Localization of nicotinic receptor subunit mRNAs in monkey brain by in situ hybridization. J Comp Neurol 425:58–69. 10940942 10.1002/1096-9861(20000911)425:1<58::aid-cne6>3.0.co;2-x

[B422] Quina LA Harris J Zeng H Turner EE (2017) Specific connections of the interpeduncular subnuclei reveal distinct components of the habenulopeduncular pathway. J Comp Neurol 525:2632–2656. 28387937 10.1002/cne.24221 PMC5873981

[B423] Quina LA Wang S Ng L Turner EE (2009) Brn3a and Nurr1 mediate a gene regulatory pathway for habenula development. J Neurosci 29:14309–14322. 19906978 10.1523/JNEUROSCI.2430-09.2009 PMC2802832

[B424] Ramirez-Latorre J Yu CR Qu X Perin F Karlin A Role L (1996) Functional contributions of alpha5 subunit to neuronal acetylcholine receptor channels. Nature 380:347–351. 8598930 10.1038/380347a0

[B425] Ramón y Cajal S (1953) Histologie du système nerveux de l’homme & des vertébrés, Consejo Superior de Investigaciones Científicas, Instituto Ramón y Cajal, Madrid.

[B426] Raybuck JD Gould TJ (2009) Nicotine withdrawal-induced deficits in trace fear conditioning in C57BL/6 mice--a role for high-affinity beta2 subunit-containing nicotinic acetylcholine receptors. Eur J Neurosci 29:377–387. 19200240 10.1111/j.1460-9568.2008.06580.x PMC2746945

[B427] Reavill C Walther B Stolerman IP Testa B (1990) Behavioural and pharmacokinetic studies on nicotine, cytisine and lobeline. Neuropharmacology 29:619–624. 2385332 10.1016/0028-3908(90)90022-j

[B428] Ren J Qin C Hu F Tan J Qiu L Zhao S Feng G Luo M (2011) Habenula “cholinergic” neurons co-release glutamate and acetylcholine and activate postsynaptic neurons via distinct transmission modes. Neuron 69:445–452. 21315256 10.1016/j.neuron.2010.12.038

[B429] Ren T Sagar SM (1992) Induction of c-fos immunostaining in the rat brain after the systemic administration of nicotine. Brain Res Bull 29:589–597. 1422856 10.1016/0361-9230(92)90127-j

[B430] Reperant C Pons S Dufour E Rollema H Gardier AM Maskos U (2010) Effect of the alpha4beta2* nicotinic acetylcholine receptor partial agonist varenicline on dopamine release in beta2 knock-out mice with selective re-expression of the beta2 subunit in the ventral tegmental area. Neuropharmacology 58:346–350. 19887076 10.1016/j.neuropharm.2009.10.007

[B431] Reuben M Boye S Clarke PB (2000) Nicotinic receptors modulating somatodendritic and terminal dopamine release differ pharmacologically. Eur J Pharmacol 393:39–49. 10770996 10.1016/s0014-2999(00)00004-2

[B432] Reus VI Obach RS Coe JW Faessel H Rollema H Watsky E Reeves K (2007) Varenicline: new treatment with efficacy in smoking cessation. Drugs Today (Barc) 43:65–75. 17353944 10.1358/dot.2007.43.2.1069956

[B433] Rice ME Cragg SJ (2004) Nicotine amplifies reward-related dopamine signals in striatum. Nat Neurosci 7:583–584. 15146188 10.1038/nn1244

[B434] Rinaman L Baker EA Hoffman GE Stricker EM Verbalis JG (1998) Medullary c-Fos activation in rats after ingestion of a satiating meal. Am J Physiol 275:R262–R268. 9688987 10.1152/ajpregu.1998.275.1.R262

[B435] Risner ME Goldberg SR (1983) A comparison of nicotine and cocaine self-administration in the dog: fixed-ratio and progressive-ratio schedules of intravenous drug infusion. J Pharmacol Exp Ther 224:319–326. 6822957

[B436] Rodd ZA Bell RL Kuc KA Zhang Y Murphy JM McBride WJ (2005) Intracranial self-administration of cocaine within the posterior ventral tegmental area of Wistar rats: evidence for involvement of serotonin-3 receptors and dopamine neurons. J Pharmacol Exp Ther 313:134–145. 15650115 10.1124/jpet.104.075952

[B437] Rodd-Henricks ZA McKinzie DL Crile RS Murphy JM McBride WJ (2000) Regional heterogeneity for the intracranial self-administration of ethanol within the ventral tegmental area of female Wistar rats. Psychopharmacology (Berl) 149:217–224. 10823401 10.1007/s002139900347

[B438] Rodrigo J Springall DR Uttenthal O Bentura ML Abadia-Molina F Riveros-Moreno V Martínez-Murillo R Polak JM Moncada S (1994) Localization of nitric oxide synthase in the adult rat brain. Philos Trans R Soc Lond B Biol Sci 345:175–221. 7526408 10.1098/rstb.1994.0096

[B439] Rollema H Chambers LK Coe JW Glowa J Hurst RS Lebel LA Lu Y Mansbach RS Mather RJ Rovetti CC, (2007) Pharmacological profile of the alpha4beta2 nicotinic acetylcholine receptor partial agonist varenicline, an effective smoking cessation aid. Neuropharmacology 52:985–994. 17157884 10.1016/j.neuropharm.2006.10.016

[B440] Rollema H Shrikhande A Ward KM Tingley FD 3rd Coe JW O’Neill BT Tseng E Wang EQ Mather RJ Hurst RS, (2010) Pre-clinical properties of the alpha4beta2 nicotinic acetylcholine receptor partial agonists varenicline, cytisine and dianicline translate to clinical efficacy for nicotine dependence. Br J Pharmacol 160:334–345. 20331614 10.1111/j.1476-5381.2010.00682.x PMC2874855

[B441] Root DH Mejias-Aponte CA Qi J Morales M (2014a) Role of glutamatergic projections from ventral tegmental area to lateral habenula in aversive conditioning. J Neurosci 34:13906–13910. 25319687 10.1523/JNEUROSCI.2029-14.2014 PMC4198536

[B442] Root DH Mejias-Aponte CA Zhang S Wang HL Hoffman AF Lupica CR Morales M (2014b) Single rodent mesohabenular axons release glutamate and GABA. Nat Neurosci 17:1543–1551. 25242304 10.1038/nn.3823 PMC4843828

[B443] Rose JE Behm FM Levin ED (1993) Role of nicotine dose and sensory cues in the regulation of smoke intake. Pharmacol Biochem Behav 44:891–900. 8469698 10.1016/0091-3057(93)90021-k

[B444] Rose JE Dehkordi O Manaye KF Millis RM Cianaki SA Jayam-Trouth A (2016) The sensory impact of nicotine on noradrenergic and dopaminergic neurons of the nicotine reward - addiction neurocircuitry. J Addict Res Ther 7:7. 10.4172/2155-6105.1000274 PMC4916769 27347434

[B445] Rose JE Tashkin DP Ertle A Zinser MC Lafer R (1985) Sensory blockade of smoking satisfaction. Pharmacol Biochem Behav 23:289–293. 4059314 10.1016/0091-3057(85)90572-6

[B446] Rossi S Singer S Shearman E Sershen H Lajtha A (2005) The effects of cholinergic and dopaminergic antagonists on nicotine-induced cerebral neurotransmitter changes. Neurochem Res 30:541–558. 16076024 10.1007/s11064-005-2689-x

[B447] Rostron BL Chang CM Pechacek TF (2014) Estimation of cigarette smoking-attributable morbidity in the United States. JAMA Intern Med 174:1922–1928. 25317719 10.1001/jamainternmed.2014.5219

[B448] Saccone SF Hinrichs AL Saccone NL Chase GA Konvicka K Madden PA Breslau N Johnson EO Hatsukami D Pomerleau O, (2007) Cholinergic nicotinic receptor genes implicated in a nicotine dependence association study targeting 348 candidate genes with 3713 SNPs. Hum Mol Genet 16:36–49. 17135278 10.1093/hmg/ddl438 PMC2270437

[B449] Sachs DP Leischow SJ (1991) Pharmacologic approaches to smoking cessation. Clin Chest Med 12:769–791. 1747993

[B450] Sakoori K Murphy NP (2009) Enhanced nicotine sensitivity in nociceptin/orphanin FQ receptor knockout mice. Neuropharmacology 56:896–904. 19371589 10.1016/j.neuropharm.2009.01.016

[B451] Salas R Cook KD Bassetto L De Biasi M (2004a) The alpha3 and beta4 nicotinic acetylcholine receptor subunits are necessary for nicotine-induced seizures and hypolocomotion in mice. Neuropharmacology 47:401–407. 15275829 10.1016/j.neuropharm.2004.05.002

[B452] Salas R Main A Gangitano D De Biasi M (2007) Decreased withdrawal symptoms but normal tolerance to nicotine in mice null for the alpha7 nicotinic acetylcholine receptor subunit. Neuropharmacology 53:863–869. 17920082 10.1016/j.neuropharm.2007.08.017 PMC2149846

[B453] Salas R Pieri F De Biasi M (2004b) Decreased signs of nicotine withdrawal in mice null for the beta4 nicotinic acetylcholine receptor subunit. J Neurosci 24:10035–10039. 15537871 10.1523/JNEUROSCI.1939-04.2004 PMC6730195

[B454] Salas R Sturm R Boulter J De Biasi M (2009) Nicotinic receptors in the habenulo-interpeduncular system are necessary for nicotine withdrawal in mice. J Neurosci 29:3014–3018. 19279237 10.1523/JNEUROSCI.4934-08.2009 PMC3862238

[B455] Salminen O Drapeau JA McIntosh JM Collins AC Marks MJ Grady SR (2007) Pharmacology of alpha-conotoxin MII-sensitive subtypes of nicotinic acetylcholine receptors isolated by breeding of null mutant mice. Mol Pharmacol 71:1563–1571. 17341654 10.1124/mol.106.031492

[B456] Salminen O Murphy KL McIntosh JM Drago J Marks MJ Collins AC Grady SR (2004) Subunit composition and pharmacology of two classes of striatal presynaptic nicotinic acetylcholine receptors mediating dopamine release in mice. Mol Pharmacol 65:1526–1535. 15155845 10.1124/mol.65.6.1526

[B457] Salminen O Seppä T Gäddnäs H Ahtee L (2000) Effect of acute nicotine on Fos protein expression in rat brain during chronic nicotine and its withdrawal. Pharmacol Biochem Behav 66:87–93. 10837847 10.1016/s0091-3057(00)00203-3

[B458] Sánchez-Catalán MJ Hipólito L Zornoza T Polache A Granero L (2009) Motor stimulant effects of ethanol and acetaldehyde injected into the posterior ventral tegmental area of rats: role of opioid receptors. Psychopharmacology (Berl) 204:641–653. 19238363 10.1007/s00213-009-1495-6

[B459] Sanjakdar SS Maldoon PP Marks MJ Brunzell DH Maskos U McIntosh JM Bowers MS Damaj MI (2015) Differential roles of α6β2* and α4β2* neuronal nicotinic receptors in nicotine- and cocaine-conditioned reward in mice. Neuropsychopharmacology 40:350–360. 25035086 10.1038/npp.2014.177 PMC4443947

[B460] Santoro B Chen S Luthi A Pavlidis P Shumyatsky GP Tibbs GR Siegelbaum SA (2000) Molecular and functional heterogeneity of hyperpolarization-activated pacemaker channels in the mouse CNS. J Neurosci 20:5264–5275. 10884310 10.1523/JNEUROSCI.20-14-05264.2000 PMC6772310

[B461] Sargent PB (1993) The diversity of neuronal nicotinic acetylcholine receptors. Annu Rev Neurosci 16:403–443. 7681637 10.1146/annurev.ne.16.030193.002155

[B462] Sartor CE Lessov-Schlaggar CN Scherrer JF Bucholz KK Madden PA Pergadia ML Grant JD Jacob T Xian H (2010) Initial response to cigarettes predicts rate of progression to regular smoking: findings from an offspring-of-twins design. Addict Behav 35:771–778. 20385446 10.1016/j.addbeh.2010.03.004 PMC2872050

[B463] Sastry BR (1978) Effects of substance P, acetylcholine and stimulation of habenula on rat interpeduncular neuronal activity. Brain Res 144:404–410. 638771 10.1016/0006-8993(78)90168-3

[B464] Schaefer GJ Michael RP (1986) Task-specific effects of nicotine in rats. Intracranial self-stimulation and locomotor activity. Neuropharmacology 25:125–131. 3703168 10.1016/0028-3908(86)90033-x

[B465] Schiffer H Atienza J Reichard H, (2020) The selective gpr139 agonist tak-041 reverses anhedonia and social interaction deficits in rodent models related to negative symptoms in schizophrenia. Schizophr Bull 46:S106–S107.

[B466] Schilström B Nomikos GG Nisell M Hertel P Svensson TH (1998) N-methyl-D-aspartate receptor antagonism in the ventral tegmental area diminishes the systemic nicotine-induced dopamine release in the nucleus accumbens. Neuroscience 82:781–789. 9483535 10.1016/s0306-4522(97)00243-1

[B467] Schneider NG Jarvik ME (1984) Time course of smoking withdrawal symptoms as a function of nicotine replacement. Psychopharmacology (Berl) 82:143–144. 6420825 10.1007/BF00426399

[B468] Scholze P Huck S (2020) The alpha5 nicotinic acetylcholine receptor subunit differentially modulates alpha4beta2(*) and alpha3beta4(*) receptors. Front Synaptic Neurosci 12:607959. 33343327 10.3389/fnsyn.2020.607959 PMC7744819

[B469] Schultz W (1986) Responses of midbrain dopamine neurons to behavioral trigger stimuli in the monkey. J Neurophysiol 56:1439–1461. 3794777 10.1152/jn.1986.56.5.1439

[B470] Schuster RM Pachas GN Stoeckel L Cather C Nadal M Mischoulon D Schoenfeld DA Zhang H Ulysse C Dodds EB, (2018) Phase IIb trial of an α7 nicotinic receptor partial agonist with and without nicotine patch for withdrawal-associated cognitive deficits and tobacco abstinence. J Clin Psychopharmacol 38:307–316. 29912798 10.1097/JCP.0000000000000919 PMC6019566

[B471] Schwartz GJ (2000) The role of gastrointestinal vagal afferents in the control of food intake: current prospects. Nutrition 16:866–873. 11054591 10.1016/s0899-9007(00)00464-0

[B472] Schwartz RD Lehmann J Kellar KJ (1984) Presynaptic nicotinic cholinergic receptors labeled by [3H]acetylcholine on catecholamine and serotonin axons in brain. J Neurochem 42:1495–1498. 6707650 10.1111/j.1471-4159.1984.tb02818.x

[B473] Sciaccaluga M Moriconi C Martinello K Catalano M Bermudez I Stitzel JA Maskos U Fucile S (2015) Crucial role of nicotinic α5 subunit variants for Ca2+ fluxes in ventral midbrain neurons. FASEB J 29:3389–3398. 25911614 10.1096/fj.14-268102 PMC4511205

[B474] Scott TR Yaxley S Sienkiewicz ZJ Rolls ET (1986) Gustatory responses in the nucleus tractus solitarius of the alert cynomolgus monkey. J Neurophysiol 55:182–200. 3950684 10.1152/jn.1986.55.1.182

[B475] Sellings LH Baharnouri G McQuade LE Clarke PB (2008) Rewarding and aversive effects of nicotine are segregated within the nucleus accumbens. Eur J Neurosci 28:342–352. 18702705 10.1111/j.1460-9568.2008.06341.x

[B476] Shao W Wang D Chiang YT Ip W Zhu L Xu F Columbus J Belsham DD Irwin DM Zhang H, (2013) The Wnt signaling pathway effector TCF7L2 controls gut and brain proglucagon gene expression and glucose homeostasis. Diabetes 62:789–800. 22966074 10.2337/db12-0365 PMC3581223

[B477] Sheffield EB Quick MW Lester RA (2000) Nicotinic acetylcholine receptor subunit mRNA expression and channel function in medial habenula neurons. Neuropharmacology 39:2591–2603. 11044729 10.1016/s0028-3908(00)00138-6

[B478] Sherafat Y Bautista M Fowler JP Chen E Ahmed A Fowler CD (2020) The interpeduncular-ventral hippocampus pathway mediates active stress coping and natural reward. eNeuro 7:7. 10.1523/ENEURO.0191-20.2020 PMC7688303 33139320

[B479] Sherva R Wilhelmsen K Pomerleau CS Chasse SA Rice JP Snedecor SM Bierut LJ Neuman RJ Pomerleau OF (2008) Association of a single nucleotide polymorphism in neuronal acetylcholine receptor subunit alpha 5 (CHRNA5) with smoking status and with ‘pleasurable buzz’ during early experimentation with smoking. Addiction 103:1544–1552. 18783506 10.1111/j.1360-0443.2008.02279.x PMC2582398

[B480] Shibata H Suzuki T (1984) Efferent projections of the interpeduncular complex in the rat, with special reference to its subnuclei: a retrograde horseradish peroxidase study. Brain Res 296:345–349. 6704742 10.1016/0006-8993(84)90071-4

[B481] Shiffman SM Jarvik ME (1976) Smoking withdrawal symptoms in two weeks of abstinence. Psychopharmacology (Berl) 50:35–39. 827760 10.1007/BF00634151

[B482] Shih PY Engle SE Oh G Deshpande P Puskar NL Lester HA Drenan RM (2014) Differential expression and function of nicotinic acetylcholine receptors in subdivisions of medial habenula. J Neurosci 34:9789–9802. 25031416 10.1523/JNEUROSCI.0476-14.2014 PMC4099552

[B483] Shih PY McIntosh JM Drenan RM (2015) Nicotine dependence reveals distinct responses from neurons and their resident nicotinic receptors in medial habenula. Mol Pharmacol 88:1035–1044. 26429939 10.1124/mol.115.101444 PMC4658593

[B484] Shippenberg TS Bals-Kubik R Herz A (1993) Examination of the neurochemical substrates mediating the motivational effects of opioids: role of the mesolimbic dopamine system and D-1 vs. D-2 dopamine receptors. J Pharmacol Exp Ther 265:53–59. 8386244

[B485] Shoaib M Bizarro L (2005) Deficits in a sustained attention task following nicotine withdrawal in rats. Psychopharmacology (Berl) 178:211–222. 15338107 10.1007/s00213-004-2004-6

[B486] Shoaib M Schindler CW Goldberg SR (1997) Nicotine self-administration in rats: strain and nicotine pre-exposure effects on acquisition. Psychopharmacology (Berl) 129:35–43. 9122361 10.1007/s002130050159

[B487] Shumake J Edwards E Gonzalez-Lima F (2003) Opposite metabolic changes in the habenula and ventral tegmental area of a genetic model of helpless behavior. Brain Res 963:274–281. 12560133 10.1016/s0006-8993(02)04048-9

[B488] Simons CT Boucher Y Carstens MI Carstens E (2006) Nicotine suppression of gustatory responses of neurons in the nucleus of the solitary tract. J Neurophysiol 96:1877–1886. 16837661 10.1152/jn.00345.2006

[B489] Singer G Wallace M (1984) Effects of 6-OHDA lesions in the nucleus accumbens on the acquisition of self injection of heroin under schedule and non schedule conditions in rats. Pharmacol Biochem Behav 20:807–809. 6429675 10.1016/0091-3057(84)90204-1

[B490] Slimak MA Ables JL Frahm S Antolin-Fontes B Santos-Torres J Moretti M Gotti C Ibañez-Tallon I (2014) Habenular expression of rare missense variants of the β4 nicotinic receptor subunit alters nicotine consumption. Front Hum Neurosci 8:12. 24478678 10.3389/fnhum.2014.00012 PMC3902282

[B491] Smaha LA Kaelber WW (1973) Efferent fiber projections of the habenula and the interpeduncular nucleus. An experimental study in the opossum and cat. Exp Brain Res 16:291–308. 4686612 10.1007/BF00233332

[B492] Soria-Gómez E Busquets-Garcia A Hu F Mehidi A Cannich A Roux L Louit I Alonso L Wiesner T Georges F, (2015) Habenular CB1 receptors control the expression of aversive memories. Neuron 88:306–313. 26412490 10.1016/j.neuron.2015.08.035

[B493] Sotomayor-Zárate R Gysling K Busto UE Cassels BK Tampier L Quintanilla ME (2013) Varenicline and cytisine: two nicotinic acetylcholine receptor ligands reduce ethanol intake in University of Chile bibulous rats. Psychopharmacology (Berl) 227:287–298. 23344555 10.1007/s00213-013-2974-3

[B494] Spealman RD (1983) Maintenance of behavior by postponement of scheduled injections of nicotine in squirrel monkeys. J Pharmacol Exp Ther 227:154–159. 6684684

[B495] Spealman RD Goldberg SR (1978) Drug self-administration by laboratory animals: control by schedules of reinforcement. Annu Rev Pharmacol Toxicol 18:313–339. 348062 10.1146/annurev.pa.18.040178.001525

[B496] Spealman RD Goldberg SR (1982) Maintenance of schedule-controlled behavior by intravenous injections of nicotine in squirrel monkeys. J Pharmacol Exp Ther 223:402–408. 7131295

[B497] Sperlágh B Kittel A Lajtha A Vizi ES (1995) ATP acts as fast neurotransmitter in rat habenula: neurochemical and enzymecytochemical evidence. Neuroscience 66:915–920. 7651618 10.1016/0306-4522(94)00588-v

[B498] Sperlágh B Maglóczky Z Vizi ES Freund TF (1998) The triangular septal nucleus as the major source of ATP release in the rat habenula: a combined neurochemical and morphological study. Neuroscience 86:1195–1207. 9697126 10.1016/s0306-4522(98)00026-8

[B499] Spina L Fenu S Longoni R Rivas E Di Chiara G (2006) Nicotine-conditioned single-trial place preference: selective role of nucleus accumbens shell dopamine D1 receptors in acquisition. Psychopharmacology (Berl) 184:447–455. 16341849 10.1007/s00213-005-0211-4

[B500] Staley JK Krishnan-Sarin S Cosgrove KP Krantzler E Frohlich E Perry E Dubin JA Estok K Brenner E Baldwin RM, (2006) Human tobacco smokers in early abstinence have higher levels of beta2* nicotinic acetylcholine receptors than nonsmokers. J Neurosci 26:8707–8714. 16928859 10.1523/JNEUROSCI.0546-06.2006 PMC6674379

[B501] Stamatakis AM Jennings JH Ung RL Blair GA Weinberg RJ Neve RL Boyce F Mattis J Ramakrishnan C Deisseroth K, (2013) A unique population of ventral tegmental area neurons inhibits the lateral habenula to promote reward. Neuron 80:1039–1053. 24267654 10.1016/j.neuron.2013.08.023 PMC3873746

[B502] Statistics NCH (2018) National Health Interview Survey, Centers for Disease Control and Prevention, Atlanta, GA.

[B503] Steensland P Simms JA Holgate J Richards JK Bartlett SE (2007) Varenicline, an alpha4beta2 nicotinic acetylcholine receptor partial agonist, selectively decreases ethanol consumption and seeking. Proc Natl Acad Sci USA 104:12518–12523. 17626178 10.1073/pnas.0705368104 PMC1914040

[B504] Steidl S O’Sullivan S Pilat D Bubula N Brown J Vezina P (2017a) Operant responding for optogenetic excitation of LDTg inputs to the VTA requires D1 and D2 dopamine receptor activation in the NAcc. Behav Brain Res 333:161–170. 28666837 10.1016/j.bbr.2017.06.045 PMC5564287

[B505] Steidl S Veverka K (2015) Optogenetic excitation of LDTg axons in the VTA reinforces operant responding in rats. Brain Res 1614:86–93. 25911581 10.1016/j.brainres.2015.04.021

[B506] Steidl S Wang H Ordonez M Zhang S Morales M (2017b) Optogenetic excitation in the ventral tegmental area of glutamatergic or cholinergic inputs from the laterodorsal tegmental area drives reward. Eur J Neurosci 45:559–571. 27740714 10.1111/ejn.13436

[B507] Stephenson DT Coskran TM Kelly MP Kleiman RJ Morton D O’Neill SM Schmidt CJ Weinberg RJ Menniti FS (2012) The distribution of phosphodiesterase 2A in the rat brain. Neuroscience 226:145–155. 23000621 10.1016/j.neuroscience.2012.09.011 PMC4409981

[B508] Stephenson DT Coskran TM Wilhelms MB Adamowicz WO O’Donnell MM Muravnick KB Menniti FS Kleiman RJ Morton D (2009) Immunohistochemical localization of phosphodiesterase 2A in multiple mammalian species. J Histochem Cytochem 57:933–949. 19506089 10.1369/jhc.2009.953471 PMC2746727

[B509] Stevens VL Bierut LJ Talbot JT Wang JC Sun J Hinrichs AL Thun MJ Goate A Calle EE (2008) Nicotinic receptor gene variants influence susceptibility to heavy smoking. Cancer Epidemiol Biomarkers Prev 17:3517–3525. 19029397 10.1158/1055-9965.EPI-08-0585 PMC2614129

[B510] Stoker AK Marks MJ Markou A (2015) Null mutation of the β2 nicotinic acetylcholine receptor subunit attenuates nicotine withdrawal-induced anhedonia in mice. Eur J Pharmacol 753:146–150. 25107281 10.1016/j.ejphar.2014.05.062 PMC4318782

[B511] Stoker AK Olivier B Markou A (2012) Role of α7- and β4-containing nicotinic acetylcholine receptors in the affective and somatic aspects of nicotine withdrawal: studies in knockout mice. Behav Genet 42:423–436. 22009521 10.1007/s10519-011-9511-0 PMC3304011

[B512] Stolerman IP Fink R Jarvik ME (1973) Acute and chronic tolerance to nicotine measured by activity in rats. Psychopharmacology (Berl) 30:329–342. 10.1007/BF00429192 4722204

[B513] Stolerman IP Jarvis MJ (1995) The scientific case that nicotine is addictive. Psychopharmacology (Berl) 117:2–10, discussion 14–20. 7724697 10.1007/BF02245088

[B514] Stoveken HM Zucca S Masuho I Grill B Martemyanov KA (2020) The orphan receptor GPR139 signals via G_q/11_ to oppose opioid effects. J Biol Chem 295:10822–10830. 32576659 10.1074/jbc.AC120.014770 PMC7397111

[B515] Subramaniyan M Dani JA (2015) Dopaminergic and cholinergic learning mechanisms in nicotine addiction. Ann N Y Acad Sci 1349:46–63. 26301866 10.1111/nyas.12871 PMC4564314

[B516] Sung YJ Winkler TW de Las Fuentes L Bentley AR Brown MR Kraja AT Schwander K Ntalla I Guo X Franceschini N, ; CHARGE Neurology Working Group; COGENT-Kidney Consortium; GIANT Consortium; Lifelines Cohort Study (2018) A large-scale multi-ancestry genome-wide study accounting for smoking behavior identifies multiple significant loci for blood pressure. Am J Hum Genet 102:375–400. 29455858 10.1016/j.ajhg.2018.01.015 PMC5985266

[B517] Szőnyi A Sos KE Nyilas R Schlingloff D Domonkos A Takács VT Pósfai B Hegedüs P Priestley JB Gundlach AL, (2019) Brainstem nucleus incertus controls contextual memory formation. Science 364:364. 10.1126/science.aaw0445 PMC7210779 31123108

[B518] Tan H Bishop SF Lauzon NM Sun N Laviolette SR (2009) Chronic nicotine exposure switches the functional role of mesolimbic dopamine transmission in the processing of nicotine’s rewarding and aversive effects. Neuropharmacology 56:741–751. 19133278 10.1016/j.neuropharm.2008.12.008

[B519] Tapia L Kuryatov A Lindstrom J (2007) Ca2+ permeability of the (alpha4)3(beta2)2 stoichiometry greatly exceeds that of (alpha4)2(beta2)3 human acetylcholine receptors. Mol Pharmacol 71:769–776. 17132685 10.1124/mol.106.030445

[B520] Tapper AR McKinney SL Nashmi R Schwarz J Deshpande P Labarca C Whiteaker P Marks MJ Collins AC Lester HA (2004) Nicotine activation of alpha4* receptors: sufficient for reward, tolerance, and sensitization. Science 306:1029–1032. 15528443 10.1126/science.1099420

[B521] Thiruchselvam T Malik S Le Foll B (2017) A review of positron emission tomography studies exploring the dopaminergic system in substance use with a focus on tobacco as a co-variate. Am J Drug Alcohol Abuse 43:197–214. 27901585 10.1080/00952990.2016.1257633 PMC5584784

[B522] Thompson R (1960) Interpeduncular nucleus and avoidance conditioning in the rat. Science 132:1551–1553. 13776611 10.1126/science.132.3439.1551

[B523] Thorgeirsson TE Geller F Sulem P Rafnar T Wiste A Magnusson KP Manolescu A Thorleifsson G Stefansson H Ingason A, (2008) A variant associated with nicotine dependence, lung cancer and peripheral arterial disease. Nature 452:638–642. 18385739 10.1038/nature06846 PMC4539558

[B524] Thorgeirsson TE Gudbjartsson DF Surakka I Vink JM Amin N Geller F Sulem P Rafnar T Esko T Walter S, ; ENGAGE Consortium (2010) Sequence variants at CHRNB3-CHRNA6 and CYP2A6 affect smoking behavior. Nat Genet 42:448–453. 20418888 10.1038/ng.573 PMC3080600

[B525] Threlfell S Lalic T Platt NJ Jennings KA Deisseroth K Cragg SJ (2012) Striatal dopamine release is triggered by synchronized activity in cholinergic interneurons. Neuron 75:58–64. 22794260 10.1016/j.neuron.2012.04.038

[B526] Timmermann DB Sandager-Nielsen K Dyhring T Smith M Jacobsen AM Nielsen EØ Grunnet M Christensen JK Peters D Kohlhaas K, (2012) Augmentation of cognitive function by NS9283, a stoichiometry-dependent positive allosteric modulator of α2- and α4-containing nicotinic acetylcholine receptors. Br J Pharmacol 167:164–182. 22506660 10.1111/j.1476-5381.2012.01989.x PMC3448921

[B527] Tolu S Eddine R Marti F David V Graupner M Pons S Baudonnat M Husson M Besson M Reperant C, (2013) Co-activation of VTA DA and GABA neurons mediates nicotine reinforcement. Mol Psychiatry 18:382–393. 22751493 10.1038/mp.2012.83

[B528] Trigo JM Zimmer A Maldonado R (2009) Nicotine anxiogenic and rewarding effects are decreased in mice lacking beta-endorphin. Neuropharmacology 56:1147–1153. 19376143 10.1016/j.neuropharm.2009.03.013 PMC2857754

[B529] Tuesta LM Chen Z Duncan A Fowler CD Ishikawa M Lee BR Liu XA Lu Q Cameron M Hayes MR, (2017) GLP-1 acts on habenular avoidance circuits to control nicotine intake. Nat Neurosci 20:708–716. 28368384 10.1038/nn.4540 PMC5541856

[B530] Tuesta LM Fowler CD Kenny PJ (2011) Recent advances in understanding nicotinic receptor signaling mechanisms that regulate drug self-administration behavior. Biochem Pharmacol 82:984–995. 21740894 10.1016/j.bcp.2011.06.026 PMC3163076

[B531] van Bloemendaal L Ten Kulve JS la Fleur SE Ijzerman RG Diamant M (2014) Effects of glucagon-like peptide 1 on appetite and body weight: focus on the CNS. J Endocrinol 221:T1–T16. 24323912 10.1530/JOE-13-0414

[B532] Vazquez-Roque MI Camilleri M Vella A Carlson P Laugen J Zinsmeister AR (2011) Association of TCF7L2 allelic variations with gastric function, satiation, and GLP-1 levels. Clin Transl Sci 4:183–187. 21707949 10.1111/j.1752-8062.2011.00284.x PMC3125688

[B533] Viswanath H Carter AQ Baldwin PR Molfese DL Salas R (2014) The medial habenula: still neglected. Front Hum Neurosci 7:931. 24478666 10.3389/fnhum.2013.00931 PMC3894476

[B534] Wada E Wada K Boulter J Deneris E Heinemann S Patrick J Swanson LW (1989) Distribution of alpha 2, alpha 3, alpha 4, and beta 2 neuronal nicotinic receptor subunit mRNAs in the central nervous system: a hybridization histochemical study in the rat. J Comp Neurol 284:314–335. 2754038 10.1002/cne.902840212

[B535] Wada K Ballivet M Boulter J Connolly J Wada E Deneris ES Swanson LW Heinemann S Patrick J (1988) Functional expression of a new pharmacological subtype of brain nicotinic acetylcholine receptor. Science 240:330–334. 2832952 10.1126/science.2832952

[B536] Wageman CR Marks MJ Grady SR (2014) Effectiveness of nicotinic agonists as desensitizers at presynaptic α4β2- and α4α5β2-nicotinic acetylcholine receptors. Nicotine Tob Res 16:297–305. 24052501 10.1093/ntr/ntt146 PMC3920335

[B537] Wagner F Bernard R Derst C French L Veh RW (2016) Microarray analysis of transcripts with elevated expressions in the rat medial or lateral habenula suggest fast GABAergic excitation in the medial habenula and habenular involvement in the regulation of feeding and energy balance. Brain Struct Funct 221:4663–4689. 26888156 10.1007/s00429-016-1195-z

[B538] Wall TR Henderson BJ Voren G Wageman CR Deshpande P Cohen BN Grady SR Marks MJ Yohannes D Kenny PJ, (2017) TC299423, a novel agonist for nicotinic acetylcholine receptors. Front Pharmacol 8:641. 29033834 10.3389/fphar.2017.00641 PMC5626944

[B539] Walters CL Brown S Changeux JP Martin B Damaj MI (2006) The beta2 but not alpha7 subunit of the nicotinic acetylcholine receptor is required for nicotine-conditioned place preference in mice. Psychopharmacology (Berl) 184:339–344. 16416156 10.1007/s00213-005-0295-x

[B540] Walters CL Cleck JN Kuo YC Blendy JA (2005) Mu-opioid receptor and CREB activation are required for nicotine reward. Neuron 46:933–943. 15953421 10.1016/j.neuron.2005.05.005

[B541] Wang D Stoveken HM Zucca S Dao M Orlandi C Song C Masuho I Johnston C Opperman KJ Giles AC, (2019) Genetic behavioral screen identifies an orphan anti-opioid system. Science 365:1267–1273. 31416932 10.1126/science.aau2078 PMC7074901

[B542] Wang F Gerzanich V Wells GB Anand R Peng X Keyser K Lindstrom J (1996) Assembly of human neuronal nicotinic receptor alpha5 subunits with alpha3, beta2, and beta4 subunits. J Biol Chem 271:17656–17665. 8663494 10.1074/jbc.271.30.17656

[B543] Wang F Nelson ME Kuryatov A Olale F Cooper J Keyser K Lindstrom J (1998) Chronic nicotine treatment up-regulates human alpha3 beta2 but not alpha3 beta4 acetylcholine receptors stably transfected in human embryonic kidney cells. J Biol Chem 273:28721–28732. 9786868 10.1074/jbc.273.44.28721

[B544] Wang J Blasio A Chapman HL Doebelin C Liaw V Kuryatov A Giovanetti SM Lindstrom J Lin L Cameron MD, (2020) Promoting activity of (α4)_3_(β2)_2_ nicotinic cholinergic receptors reduces ethanol consumption. Neuropsychopharmacology 45:301–308. 31394567 10.1038/s41386-019-0475-8 PMC6901472

[B545] Wang J Kuryatov A Sriram A Jin Z Kamenecka TM Kenny PJ Lindstrom J (2015) An accessory agonist binding site promotes activation of α4β2* nicotinic acetylcholine receptors. J Biol Chem 290:13907–13918. 25869137 10.1074/jbc.M115.646786 PMC4447965

[B546] Wang JC Cruchaga C Saccone NL Bertelsen S Liu P Budde JP Duan W Fox L Grucza RA Kern J, ; COGEND collaborators and GELCC collaborators (2009) Risk for nicotine dependence and lung cancer is conferred by mRNA expression levels and amino acid change in CHRNA5. Hum Mol Genet 18:3125–3135. 19443489 10.1093/hmg/ddp231 PMC2714722

[B547] Wang S D van der Vaart A Xu Q Seneviratne C Pomerleau OF Pomerleau CS Payne TJ Ma JZ Li MD (2014) Significant associations of CHRNA2 and CHRNA6 with nicotine dependence in European American and African American populations. Hum Genet 133:575–586. 24253422 10.1007/s00439-013-1398-9 PMC3988215

[B548] Ware JJ van den Bree MB Munafò MR (2011) Association of the CHRNA5-A3-B4 gene cluster with heaviness of smoking: a meta-analysis. Nicotine Tob Res 13:1167–1175. 22071378 10.1093/ntr/ntr118 PMC3223575

[B549] Watkins SS Epping-Jordan MP Koob GF Markou A (1999) Blockade of nicotine self-administration with nicotinic antagonists in rats. Pharmacol Biochem Behav 62:743–751. 10208381 10.1016/s0091-3057(98)00226-3

[B550] Watkins SS Stinus L Koob GF Markou A (2000) Reward and somatic changes during precipitated nicotine withdrawal in rats: centrally and peripherally mediated effects. J Pharmacol Exp Ther 292:1053–1064. 10688623

[B551] Weiss RB Baker TB Cannon DS von Niederhausern A Dunn DM Matsunami N Singh NA Baird L Coon H McMahon WM, (2008) A candidate gene approach identifies the CHRNA5-A3-B4 region as a risk factor for age-dependent nicotine addiction. PLoS Genet 4:e1000125. 18618000 10.1371/journal.pgen.1000125 PMC2442220

[B552] Wen L Yang Z Cui W Li MD (2016) Crucial roles of the CHRNB3-CHRNA6 gene cluster on chromosome 8 in nicotine dependence: update and subjects for future research. Transl Psychiatry 6:e843. 27327258 10.1038/tp.2016.103 PMC4931601

[B553] West RJ Russell MA Jarvis MJ Feyerabend C (1984) Does switching to an ultra-low nicotine cigarette induce nicotine withdrawal effects? Psychopharmacology (Berl) 84:120–123. 6436879 10.1007/BF00432039

[B554] Whiteaker P McIntosh JM Luo S Collins AC Marks MJ (2000) 125I-alpha-conotoxin MII identifies a novel nicotinic acetylcholine receptor population in mouse brain. Mol Pharmacol 57:913–925. 10779374

[B555] WHO (2008) WHO Report on the Global Tobacco Epidemic, 2008: the MPOWER package, World Health Organization, Geneva.

[B556] Williams JM Gandhi KK Lu SE Steinberg ML Benowitz NL (2013) Rapid smoking may not be aversive in schizophrenia. Nicotine Tob Res 15:262–266. 22318691 10.1093/ntr/ntr314 PMC3524052

[B557] Willing AE Berthoud HR (1997) Gastric distension-induced c-fos expression in catecholaminergic neurons of rat dorsal vagal complex. Am J Physiol 272:R59–R67. 9038991 10.1152/ajpregu.1997.272.1.R59

[B558] Winzer-Serhan UH Leslie FM (1997) Codistribution of nicotinic acetylcholine receptor subunit alpha3 and beta4 mRNAs during rat brain development. J Comp Neurol 386:540–554. 9378850 10.1002/(sici)1096-9861(19971006)386:4<540::aid-cne2>3.0.co;2-2

[B559] Wise RA Bauco P Carlezon WA Jr Trojniar W (1992) Self-stimulation and drug reward mechanisms. Ann N Y Acad Sci 654:192–198. 1632583 10.1111/j.1749-6632.1992.tb25967.x

[B560] Wolfman SL Gill DF Bogdanic F Long K Al-Hasani R McCall JG Bruchas MR McGehee DS (2018) Nicotine aversion is mediated by GABAergic interpeduncular nucleus inputs to laterodorsal tegmentum. Nat Commun 9:2710. 30006624 10.1038/s41467-018-04654-2 PMC6045623

[B597] Wonnacott S (1997) Presynaptic nicotinic ACh receptors. Trends Neurosci 20:92–98. 9023878 10.1016/s0166-2236(96)10073-4

[B561] Xiao C Cho JR Zhou C Treweek JB Chan K McKinney SL Yang B Gradinaru V (2016) Cholinergic mesopontine signals govern locomotion and reward through dissociable midbrain pathways. Neuron 90:333–347. 27100197 10.1016/j.neuron.2016.03.028 PMC4840478

[B562] Xie W Kathuria H Galiatsatos P Blaha MJ Hamburg NM Robertson RM Bhatnagar A Benjamin EJ Stokes AC (2020) Association of electronic cigarette use with incident respiratory conditions among US adults from 2013 to 2018. JAMA Netw Open 3:e2020816. 33180127 10.1001/jamanetworkopen.2020.20816 PMC7662143

[B563] Xu C Sun Y Cai X You T Zhao H Li Y Zhao H (2018) Medial habenula-interpeduncular nucleus circuit contributes to anhedonia-like behavior in a rat model of depression. Front Behav Neurosci 12:238. 30356828 10.3389/fnbeh.2018.00238 PMC6189744

[B564] Yamaguchi T Danjo T Pastan I Hikida T Nakanishi S (2013) Distinct roles of segregated transmission of the septo-habenular pathway in anxiety and fear. Neuron 78:537–544. 23602500 10.1016/j.neuron.2013.02.035 PMC3654012

[B565] Yan Y Beckley NA Kim VJ Drenan RM (2019) Differential nicotinic modulation of glutamatergic and GABAergic VTA microcircuits. eNeuro 6:6. 10.1523/ENEURO.0298-19.2019 PMC6893235 31744841

[B566] Yan Y Peng C Arvin MC Jin XT Kim VJ Ramsey MD Wang Y Banala S Wokosin DL McIntosh JM, (2018) Nicotinic cholinergic receptors in VTA glutamate neurons modulate excitatory transmission. Cell Rep 23:2236–2244. 29791835 10.1016/j.celrep.2018.04.062 PMC5999341

[B567] Yang K Buhlman L Khan GM Nichols RA Jin G McIntosh JM Whiteaker P Lukas RJ Wu J (2011) Functional nicotinic acetylcholine receptors containing α6 subunits are on GABAergic neuronal boutons adherent to ventral tegmental area dopamine neurons. J Neurosci 31:2537–2548. 21325521 10.1523/JNEUROSCI.3003-10.2011 PMC3081713

[B568] Yang K Hu J Lucero L Liu Q Zheng C Zhen X Jin G Lukas RJ Wu J (2009) Distinctive nicotinic acetylcholine receptor functional phenotypes of rat ventral tegmental area dopaminergic neurons. J Physiol 587:345–361. 19047205 10.1113/jphysiol.2008.162743 PMC2670049

[B569] Yi F Brubaker PL Jin T (2005) TCF-4 mediates cell type-specific regulation of proglucagon gene expression by beta-catenin and glycogen synthase kinase-3beta. J Biol Chem 280:1457–1464. 15525634 10.1074/jbc.M411487200

[B570] Yildirim E Connor DA Gould TJ (2015) ABT-089, but not ABT-107, ameliorates nicotine withdrawal-induced cognitive deficits in C57BL6/J mice. Behav Pharmacol 26:241–248. 25426579 10.1097/FBP.0000000000000111 PMC4459497

[B571] Yoshimura RF Hogenkamp DJ Li WY Tran MB Belluzzi JD Whittemore ER Leslie FM Gee KW (2007) Negative allosteric modulation of nicotinic acetylcholine receptors blocks nicotine self-administration in rats. J Pharmacol Exp Ther 323:907–915. 17873105 10.1124/jpet.107.128751

[B572] Zangen A Ikemoto S Zadina JE Wise RA (2002) Rewarding and psychomotor stimulant effects of endomorphin-1: anteroposterior differences within the ventral tegmental area and lack of effect in nucleus accumbens. J Neurosci 22:7225–7233. 12177217 10.1523/JNEUROSCI.22-16-07225.2002 PMC6757872

[B573] Zhang H Sulzer D (2004) Frequency-dependent modulation of dopamine release by nicotine. Nat Neurosci 7:581–582. 15146187 10.1038/nn1243

[B574] Zhang J Tan L Ren Y Liang J Lin R Feng Q Zhou J Hu F Ren J Wei C, (2016) Presynaptic excitation via GABAB receptors in habenula cholinergic neurons regulates fear memory expression. Cell 166:716–728. 27426949 10.1016/j.cell.2016.06.026

[B575] Zhao-Shea R DeGroot SR Liu L Vallaster M Pang X Su Q Gao G Rando OJ Martin GE George O, (2015) Increased CRF signalling in a ventral tegmental area-interpeduncular nucleus-medial habenula circuit induces anxiety during nicotine withdrawal. Nat Commun 6:6770. 25898242 10.1038/ncomms7770 PMC4405813

[B576] Zhao-Shea R Liu L Pang X Gardner PD Tapper AR (2013) Activation of GABAergic neurons in the interpeduncular nucleus triggers physical nicotine withdrawal symptoms. Curr Biol 23:2327–2335. 24239118 10.1016/j.cub.2013.09.041 PMC3855889

[B577] Zhao-Shea R Liu L Soll LG Improgo MR Meyers EE McIntosh JM Grady SR Marks MJ Gardner PD Tapper AR (2011) Nicotine-mediated activation of dopaminergic neurons in distinct regions of the ventral tegmental area. Neuropsychopharmacology 36:1021–1032. 21289604 10.1038/npp.2010.240 PMC3077271

[B578] Zhou FM Liang Y Dani JA (2001) Endogenous nicotinic cholinergic activity regulates dopamine release in the striatum. Nat Neurosci 4:1224–1229. 11713470 10.1038/nn769

[B579] Zoli M Léna C Picciotto MR Changeux JP (1998) Identification of four classes of brain nicotinic receptors using beta2 mutant mice. J Neurosci 18:4461–4472. 9614223 10.1523/JNEUROSCI.18-12-04461.1998 PMC6792706

[B580] Zoli M Moretti M Zanardi A McIntosh JM Clementi F Gotti C (2002) Identification of the nicotinic receptor subtypes expressed on dopaminergic terminals in the rat striatum. J Neurosci 22:8785–8789. 12388584 10.1523/JNEUROSCI.22-20-08785.2002 PMC6757689

[B581] Zuo W Xiao C Gao M Hopf FW Krnjević K McIntosh JM Fu R Wu J Bekker A Ye JH (2016) Nicotine regulates activity of lateral habenula neurons via presynaptic and postsynaptic mechanisms. Sci Rep 6:32937. 27596561 10.1038/srep32937 PMC5011770

[B582] Zwart R Vijverberg HP (1998) Four pharmacologically distinct subtypes of alpha4beta2 nicotinic acetylcholine receptor expressed in Xenopus laevis oocytes. Mol Pharmacol 54:1124–1131. 9855643

